# Lewis Acid-Catalyzed Domino Inverse Electron-Demand Diels–Alder/Thermal Ring Expansion Reaction for the Synthesis of Arene-Annulated Eight-Membered Nitrogen Heterocycles

**DOI:** 10.1021/acs.orglett.5c01150

**Published:** 2025-05-01

**Authors:** Michel Große, Christopher M. Leonhardt, Patrick A. R. Campbell, Hermann A. Wegner

**Affiliations:** † Institute of Organic Chemistry, Justus Liebig University Giessen, 35392 Giessen, Germany; ‡ Center for Materials Research (LaMa), Justus Liebig University Giessen, 35392 Giessen, Germany

## Abstract

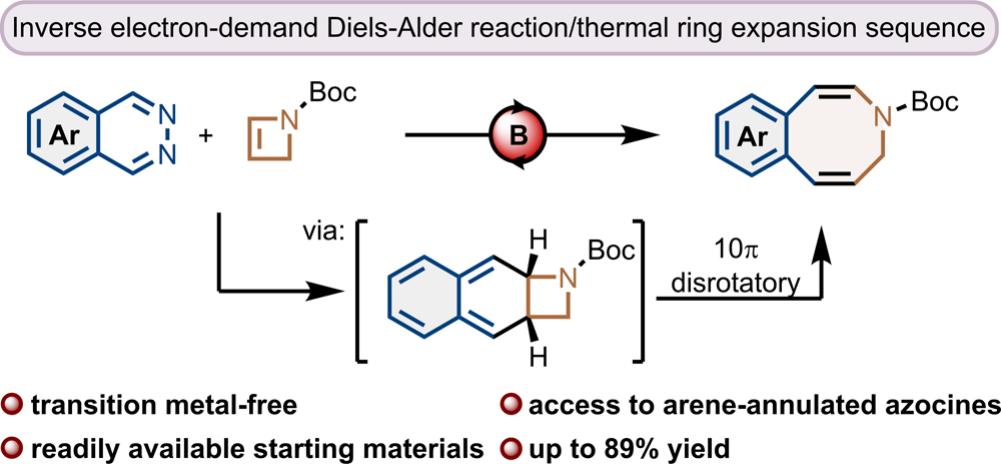

A domino inverse electron-demand Diels–Alder reaction/thermal ring expansion sequence was developed to enable the one-step synthesis of arene-annulated eight-membered nitrogen heterocycles from readily available aromatic 1,2-diazines. A boron-based, bidentate Lewis acid catalyst facilitates the initial cycloaddition of Boc-protected 2-azetine with various electron-poor and electron-rich phthalazines. The subsequent electrocyclic ring expansion furnishes azocines fused to differently substituted aromatics, a structural motif that holds vast potential for further derivatization.

Eight-membered nitrogen-containing heterocycles are widely found in biologically active natural products^1^ as well as medicinally relevant synthetic compounds.^2−5^ They are commonly regarded as privileged scaffolds for drug discovery owing to their distinctive structural features.^6^ In comparison to smaller rings, eight-membered carbo- and heterocycles offer a balance between conformational flexibility and rigidity which can lead to improved binding properties to biological targets by either allowing effective folding into enzymatic pockets^7^ or by rigidifying active conformations.^8^ In particular, benzannulated azocines are the core structural motif of a variety of synthetic compounds and natural products showing promising biological activity (Figure 1).^9−13^

**Figure 1:**
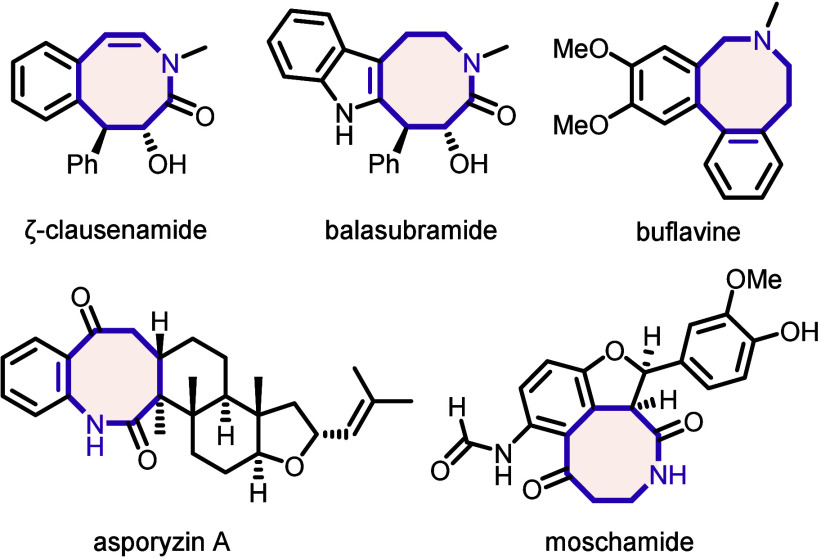
Representative examples of arene-annulated azocine alkaloids.

Consequently, extensive efforts have been made to synthetically access these benzo-fused scaffolds as well as structurally related eight-membered ring systems,^6,14−16^ primarily via ring expansion strategies^17−19^ or transition metal-promoted cyclizations.^20−23^ However, medium-sized heterocycles are still strongly underrepresented in screening libraries and drug approvals,^24^ most probably due to the persisting synthetic challenges caused by unfavorable entropic and enthalpic factors.^25,26^ Therefore, new general strategies, especially for the synthesis of benzannulated azocines, are highly desirable. In the light of sustainability and the shortage of resources, these strategies should be based on simple, readily available starting materials and work in the absence of transition metals.

In the past, we established the bidentate Lewis acid **BDLA** as an effective catalyst to promote inverse electron-demand Diels–Alder (IEDDA) reactions of phthalazines (**1**) and various dienophiles (Scheme 1a).^27−35^ These reactions usually proceed via a reactive *o*-quinodimethane (*o*-QDM) intermediate **2** which is formed after the initial IEDDA-cycloreversion sequence.^36^ This reactive intermediate can be utilized to initiate different domino processes, depending on the reaction conditions and the nature of the dienophile. Recently, we have shown that benzannulated, medium-sized carbocycles can be accessed through a photoinduced ring opening of the *o*-QDM intermediate.^33^ We wanted to further expand this methodology to the synthesis arene-annulated, eight-membered nitrogen heterocycles. We envisaged that the use of highly strained 2-azetine **3** as dienophile would afford *o*-QDM intermediate **4**, which would then react under thermal conditions to the desired benzannulated azocine **5** through a 10π disrotatory electrocylic ring expansion (Scheme 1b). Prior reports indicate that analogous 6π azacycles thermodynamically favor the formation of the bridged four-membered structure.^37^ However, we hypothesized that in azocine **5**, the formation of an annulated aromatic ring would significantly shift the equilibrium toward the ring-expanded product.

**Scheme 1:**
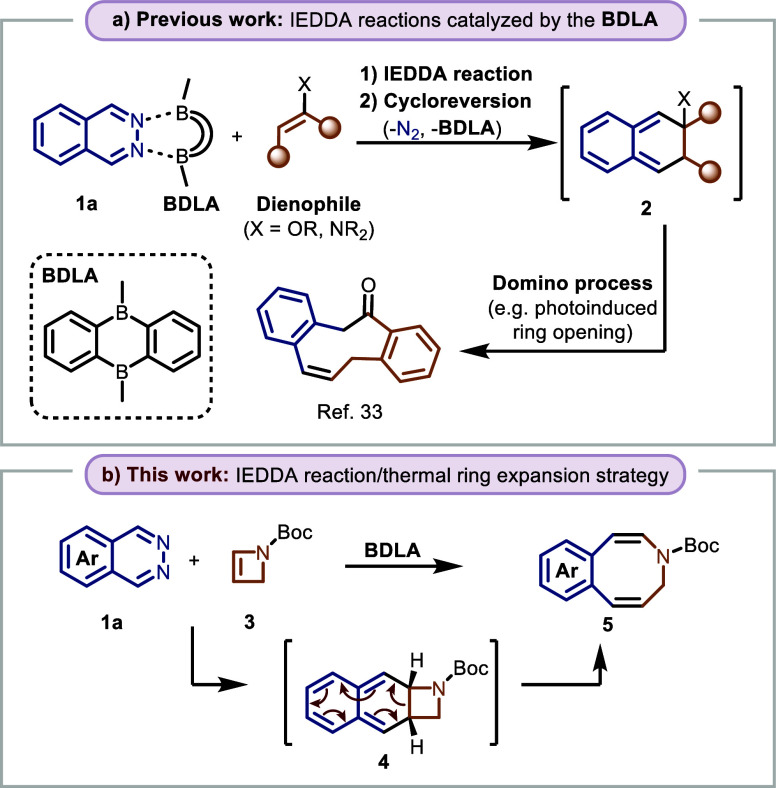
BDLA-Catalyzed IEDDA Reactions for the Construction of Medium-Sized Rings

We decided to utilize Boc-protected azetine **3** as it can be easily prepared from commercial *tert*-butyl 3-hydroxyazetidine-1-carboxylate over two steps and should allow easy functionalization of the final azocine at the nitrogen atom. We commenced our study by treating very reactive, electron-poor difluorophthalazine **1b** with azetine **3** (1.2 equiv) in the presence and absence of the **BDLA** (5 mol %) at 70 °C (Table 1, Entries 1 and 2). Without the catalyst, no visible consumption of phthalazine **1b** was observed by ^1^H NMR spectroscopic analysis and no traces of the desired product were detected. To our delight, in the presence of the **BDLA** catalyst, all of the staring material was consumed and the desired azocine **5b** could be isolated in 25% yield.

**Table 1:**
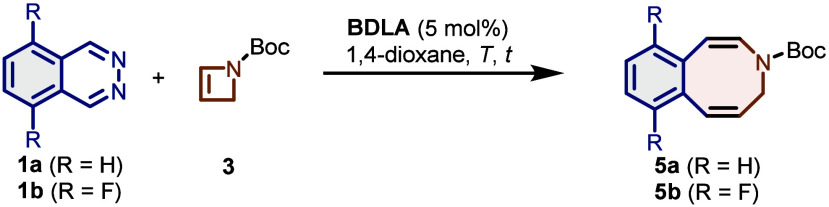
Optimization of the Reaction Conditions^a^

Entry	R	*T* (°C)	*t* (h)	Addition of **3**	Yield (%)
1^b^	F	70	20	at once	0
2	F	70	20	at once	25
3	F	70	16	over 10 h	75
4	F	80	22	over 20 h	87
5	H	70	16	over 10 h	12
6	H	90	22	over 20 h	55
7^c^	H	110	22	over 20 h	73

a Reaction conditions unless noted otherwise: phthalazine **1a** or **1b** (0.25 mmol, 1.0 equiv) and **BDLA** (13 μmol, 5.0 mol %) in 1,4-dioxane (4 mL), azetine **3** (0.31 mmol, 1.2 equiv) in 1,4-dioxane (1 mL).

b Reaction was performed without the **BDLA** catalyst.

c Reaction was performed on a 1.0 mmol scale: phthalazine **1a** (1.0 mmol, 1.0 equiv) and **BDLA** (30 μmol, 3.0 mol %) in diglyme (16 mL), and azetine **3** (1.2 mmol, 1.2 equiv) in diglyme (4 mL) yielded azocine **5a** (188 mg, 73%).

A substantial amount of different, chromatographically inseparable byproducts was obtained as well. Judging by high resolution mass spectrometry, we hypothesized that these byproducts were a mixture of regio- and stereoisomers formed by a follow-up Diels–Alder reaction of *o*-QDM intermediate **4** with another equivalent of azetine **3**. To prove this hypothesis, we treated phthalazine **1b** with an excess of azetine **3**, which suppressed the formation of azocine **5b** and resulted almost exclusively in a mixture of double Diels–Alder adducts, the structure of which was further proven by X-ray crystallography of *meso* compound **6** after partial purification via preparative HPLC (Scheme 2 and the Supporting Information (SI)).

**Scheme 2:**
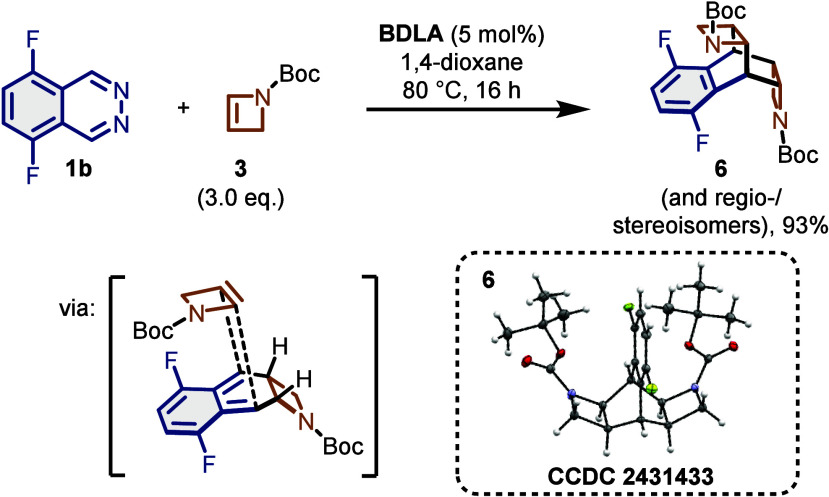
Synthesis and Crystal Structure of Double Diels–Alder Adducts

With an explanation for the byproduct formation in hand, we further optimized the conditions for the IEDDA reaction/thermal ring expansion sequence by adding azetine **3** slowly via syringe pump to a mixture of phthalazine **1b** and **BDLA** catalyst to prevent an excess of the dienophile (Table 1, Entries 3 and 4). With a longer addition time of 20 h, the isolated yield of azocine **5b** could be improved to 87%. Changing the diene to electron-neutral, unsubstituted phthalazine (**1a**) led to a significant decrease in product formation, and the temperature had to be increased to 110 °C to furnish azocine **1a** in a good yield of 73% (Table 1, Entries 5 to 7).

With optimized reaction conditions in hand, we set out to explore the scope of this transformation by testing different phthalazines and pyridazino-aromatics (**1c**–**m**), most of which can be readily synthesized from commercially available aldehydes by a convenient one-pot procedure previously developed in our laboratory (Scheme 3).^38^ As expected, all tested diazines carrying electron-withdrawing groups (**1b**–**j**) underwent the IEDDA reaction smoothly and afforded the desired azocines after ring expansion in good to very good yields up to 89% (Scheme 3). In that way, synthetically valuable functional groups such as esters (**5j**), nitro groups (**5d**) or different halogens (**5c**,**f**–**h**) were introduced at the fused aromatic ring. Furthermore, pyridine-annulated azocine **5e** was obtained in a good yield of 71% from pyridopyridazine **1e**. In the case of monohalogenated phthalazines **1f**–**h** and trifluoromethylated phthalazine **1i**, a slight increase in temperature to 90 °C was required to achieve good yields over 70%. Similarly, benzophthalazine **1k** furnished naphthoazocine **5k** in 71% yield at an elevated reaction temperature of 110 °C. Even electron-rich methyl- (**1l**) and methoxyphthalazine (**1m**) were reactive in this transformation. However, higher temperatures of 125 and 140 °C, respectively, were required for the IEDDA reaction to take place. This led to an increased formation of side products and the desired azocines **5l**,**m** were isolated in a yield of around 20%.

**Scheme 3:**
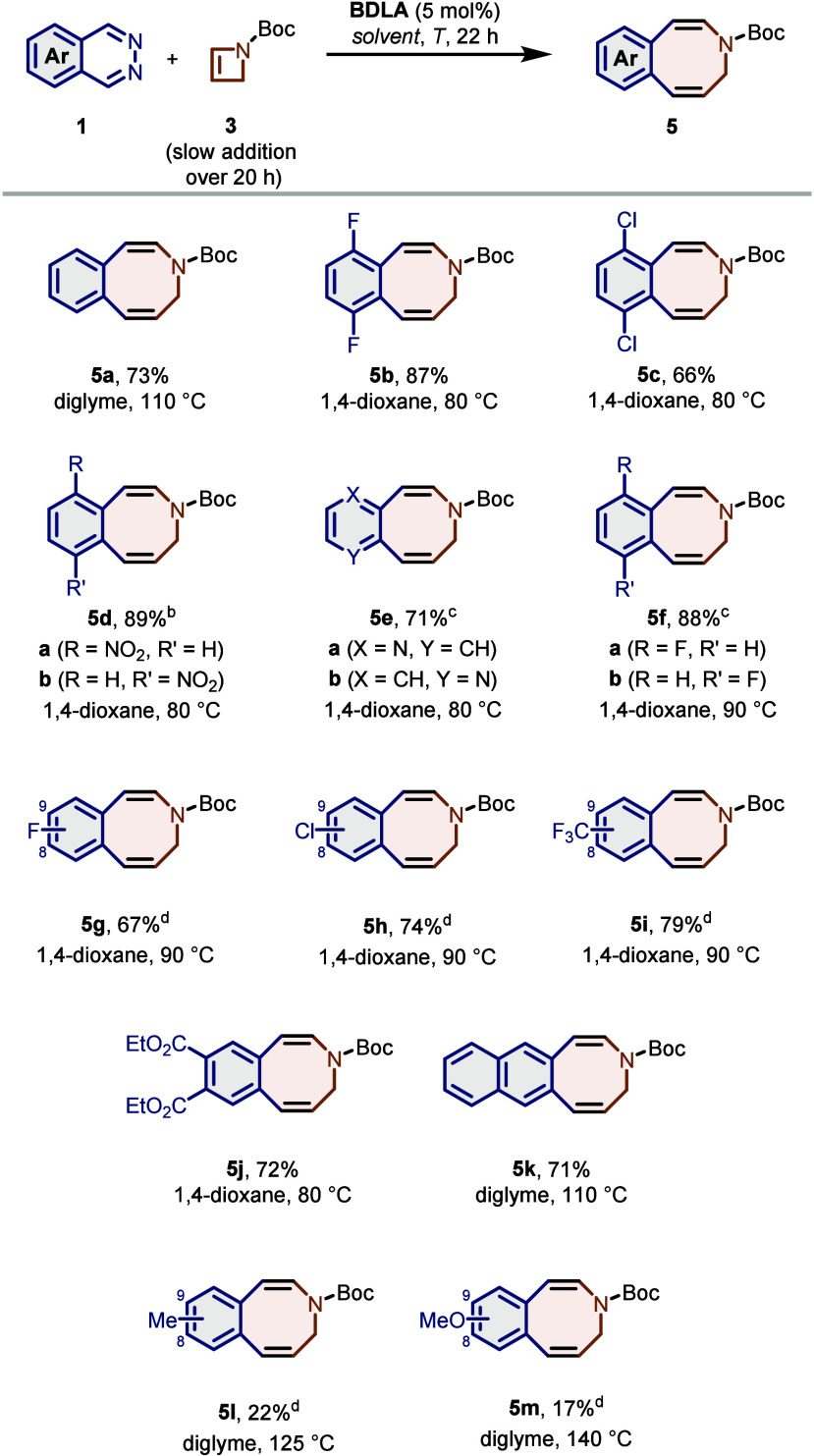
Scope of 1,2-Diazines in the IEDDA/Thermal Ring Expansion Reaction Reaction conditions: phthalazine **1a**–**m** (0.25 mmol, 1.0 equiv) and **BDLA** (13 μmol, 5.0 mol %) in 1,4-dioxane or diglyme (4 mL), azetine **3** (0.31 mmol, 1.2 equiv) in 1,4-dioxane or diglyme (1 mL). Constitutional isomers were separated (**a**, 42%; **b**, 47%). Isolated yield as a mixture of C7/C10-constitutional isomers. Isolated yield as a mixture of C8/C9-constitutional isomers.

For all asymmetrically substituted phthalazines, mixtures of regioisomers were obtained in this transformation. In the case of nitrobenzazocine **5d**, both isomers could be separated via column chromatography and were obtained in a ratio of 47:53. Most of the other nonsymmetrically substituted phthalazines yielded a similar ratio of isomers. Only in the case of pyridopyridazine **1e**, the strong polarization of the substrate led to the formation of the major isomer in a 4-fold excess (see the SI). As a final structural proof, we attempted to analyze the ring expansion products via X-ray crystallography. However, all azocines **5a**-**m** were obtained as oils or amorphous solids, and every attempt to produce single crystals failed. Unexpectedly, we observed the formation of a crystalline dimerization product of azocine **5a** during NMR measurements in nonstabilized CDCl_3_. After separation of the formed stereoisomers via preparative chiral HPLC, single crystals suitable for X-ray diffraction were obtained. The product was found to be hemiaminal ether **7** formed in the presence of water and catalytic amounts of acid (Figure 2). In that way, we were finally able to confirm the formation of the azocine ring in our IEDDA reaction/thermal ring expansion sequence.

**Figure 2:**
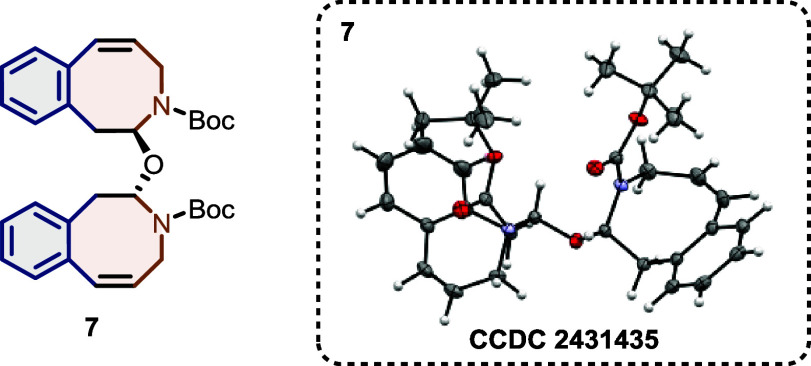
Structure of the hemiaminal ether **7** formed from azocine **5a** in acidic CDCl_3_ in the presence of water.

In summary, we have developed a novel Lewis acid-catalyzed domino IEDDA/thermal ring expansion reaction that provides rapid access to arene-annulated, eight-membered nitrogen heterocycles. The key to successfully optimizing the reaction was the very slow addition of the azetine dienophile, which suppressed the undesired follow-up Diels–Alder reaction of the highly reactive *o*-QDM intermediate and ensured its conversion via a 10π disrotatory electrocylic ring expansion. Various, readily available phthalazines and pyridazino-aromatics can be used as dienes in this transformation, giving rise to the azocine core structure fused to diversely functionalized aromatics. The electronic nature of the phthalazine was found to be a crucial parameter for this transformation, with electron-poor substrates providing the medium-sized nitrogen heterocylces in high yields up to 89%, whereas electron-neutral and -rich phthalazines required higher temperatures and showed diminished yields. The final proof of the eight-membered ring structure was provided by X-ray crystallography of a hemiaminal ether degradation product. The presented methodology holds great potential to synthetically address different, biologically active azocine natural products as well as their derivatives in order to tap into the full potential of eight-membered nitrogen heterocycles in medicinal chemistry and drug discovery.

## Data Availability

The data underlying this study are available in the published article and its Supporting Information.

## Supporting Information Available

Experimental details, analytical data, NMR spectra, and crystallographic details (PDF)
