# Supplementary material for: Lewis Acid-Catalyzed Domino Inverse Electron-Demand Diels–Alder/Thermal Ring Expansion Reaction for the Synthesis of Arene-Annulated Eight-Membered Nitrogen Heterocycles
Source: Org Lett. 2025 May 1;27(19):4893–7. doi: 10.1021/acs.orglett.5c01150 (PMC12090217; doi:10.1021/acs.orglett.5c01150)
Supplement: Supplementary file 1 — ol5c01150_si_001.pdf [file ol5c01150_si_001.pdf]

Supporting Information

Lewis Acid-Catalyzed Domino Inverse Electron-Demand Diels-Alder/Thermal Ring Expansion Reaction for the Synthesis of Arene-Annulated Eight-Membered Nitrogen Heterocycles

Michel Große[†,‡], Christopher M. Leonhardt[†,‡], Patrick A. R. Campbell[†,‡] and Hermann A. Wegner[†,‡,*]

[†]Institute of Organic Chemistry, Justus Liebig University Giessen, Heinrich-Buff-Ring 17, 35392 Giessen, Germany

[‡]Center for Materials Research (LaMa), Justus Liebig University Giessen, Heinrich-Buff-Ring 16, 35392 Giessen, Germany

*hermann.a.wegner@org.chemie.uni-giessen.de

Table of Contents

1 Experimental procedures and characterization data 3

1.1 General experimental 3

1.2 Synthesis of phthalazines 4

1.3 Synthesis of *tert*-butyl azete-1(2*H*)-carboxylate (**3**) 7

1.4 General procedure for the IEDDA reaction/thermal ring expansion sequence (**GP1**) 8

1.4.1 Synthesis of *tert*-butyl (1*Z*,5*Z*)-benzo[*d*]azocine-3(4*H*)-carboxylate (**5a**). 9

1.4.2 Synthesis of *tert*-butyl (1*Z*,5*Z*)-7,10-difluorobenzo[*d*]azocine-3(4*H*)-carboxylate (**5b**) 10

1.4.3 Synthesis of *tert*-butyl (1*Z*,5*Z*)-7,10-dichlorobenzo[*d*]azocine-3(4*H*)-carboxylate (**5c**) 11

1.4.4 Synthesis of *tert*-butyl (1*Z*,5*Z*)-10-nitrobenzo[*d*]azocine-3(4*H*)-carboxylate (**5da**) and *tert*-butyl (1*Z*,5*Z*)-7-nitrobenzo[*d*]azocine-3(4*H*)-carboxylate (**5db**) 12

1.4.5 Synthesis of *tert*-butyl (5*Z*,9*Z*)-pyrido[2,3-*d*]azocine-8(7*H*)-carboxylate (**5ea**) and *tert*-butyl (5*Z*,9*Z*)-pyrido[3,2-*d*]azocine-7(8*H*)-carboxylate (**5eb**)........ 13

1.4.6 Synthesis of *tert*-butyl (1*Z*,5*Z*)-10-fluorobenzo[*d*]azocine-3(4*H*)-carboxylate (**5fa**) and *tert*-butyl (1*Z*,5*Z*)-7-fluorobenzo[*d*]azocine-3(4*H*)-carboxylate (**5fb**) 14

1.4.7 Synthesis of *tert*-butyl (1*Z*,5*Z*)-9-fluorobenzo[*d*]azocine-3(4*H*)-carboxylate and *tert*-butyl (1*Z*,5*Z*)-8-fluorobenzo[*d*]azocine-3(4*H*)-carboxylate (**5g**) 15

1.4.8 Synthesis of *tert*-butyl (1*Z*,5*Z*)-8-chlorobenzo[*d*]azocine-3(4*H*)-carboxylate and *tert*-butyl (1*Z*,5*Z*)-9-chlorobenzo[*d*]azocine-3(4*H*)-carboxylate (**5h**) 16

1.4.9 Synthesis of *tert*-butyl (1*Z*,5*Z*)-8-(trifluoromethyl)benzo[*d*]azo-cine-3(4*H*)-carboxylate and *tert*-butyl (1*Z*,5*Z*)-9-(trifluoromethyl)-benzo[*d*]azocine-3(4*H*)-carboxylate (**5i**) 17

1.4.10 Synthesis of 3-(*tert*-butyl) 8,9-diethyl (1*Z*,5*Z*)-benzo[*d*]azo-cine-3,8,9(4*H*)-tricarboxylate (**5j**) 18

1.4.11 Synthesis of *tert*-butyl (1*Z*,5*Z*)-naphtho[2,3-*d*]azocine-3(4*H*)-carboxylate (**5k**) 19

1.4.12 Synthesis of *tert*-butyl (1*Z*,5*Z*)-9-methylbenzo[*d*]azocine-3(4*H*)-carboxylate and *tert*-butyl (1*Z*,5*Z*)-8-methylbenzo[*d*]azocine-3(4*H*)-carboxylate (**5l**) 20

1.4.13 Synthesis of *tert*-butyl (1*Z*,5*Z*)-9-methoxybenzo[*d*]azo-cine-3(4*H*)-carboxylate and *tert*-butyl (1*Z*,5*Z*)-8-methoxy-benzo[*d*]azocine-3(4*H*)-carboxylate (**5m**) 21

1.4.14 Synthesis of di-*tert*-butyl (2a*R*,3*s*,8*s*,8a*S*,9*S*,10*R*)-4,7-difluoro-2a,3,8,8a-tetrahydro-8,3-[2,3]epazetonaphtho[2,3-*b*]azete-1,11(2*H*)-dicarboxylate (**6**) and regio-/stereoisomers 22

1.4.15 Synthesis of di-*tert*-butyl 2,2'-oxy(2*S*,2'*S*,5*Z*,5'*Z*)-bis(1,4-dihydrobenzo[*d*]azocine-3(2*H*)-carboxylate) (**7**) 23

1.5 Structure elucidation and purification of side products 24

1.5.1 Double Diels-Alder adducts..... 24

1.5.2 Hemiaminal ether **7** 27

2 NMR spectra 28

3 XRD Analysis 55

3.1 General crystallographic experimental details 55

3.2 Crystallographic details of the double Diels-Alder adduct **6** 56

3.3 Crystallographic details of the hemiaminal ether **7** 64

4 References 75

1 Experimental procedures and characterization data

1.1 General experimental

Chemicals were purchased from Sigma-Aldrich, Acros Organics, Alfa Aesar, chemPUR, abcr GmbH, TCI Europe or BLDpharm. Deuterated solvents were purchased from Deutero GmbH or Sigma-Aldrich. Technical grade solvents used during work-up and purification were distilled prior to use. The Bidentate Lewis acid **BDLA** was synthesized according to the literature,[1] stored and handled in a nitrogen-filled glove box. Sensitive reactions were performed in dry glassware under nitrogen atmosphere using Schlenk techniques or in a nitrogen-filled MBRAUN UNIlab glove box. An oil bath was used for all reactions requiring heating.

NMR spectra were measured on a Bruker Avance II 200 MHz, Avance II 400 MHz or Avance III 400 MHz HD spectrometer at 25 °C if not stated otherwise. Chemical shifts (δ) are reported in parts per million (ppm) relative to residual solvent signals. Coupling constants (*J*) are reported in Hertz (Hz). Multiplicities are abbreviated as s (singlet), d (doublet), t (triplet), q (quartet) and m (multiplet). Structural assignments were made with additional information from gCOSY, gHSQC, and gHMBC experiments. Regioisomeric ratios were determined from appropriate integrals in the respective ^{1}H NMR spectra.

Flash chromatography was carried out with Silica 60 (0.04 – 0.063 mm) from Marcherey-Nagel GmbH & Co. KG. Automated flash chromatography was performed on an Advion Interchim puriFlash XS 520 Plus system using PF-30SIHP or PF-15SIHP columns. Thin layer chromatography was performed on Polygram®SIL G/UV254 from Macherey Nagel GmbH & Co. KG. Spots were visualized under UV-light and with basic $KMnO_4$ stain.

High-resolution mass spectra were recorded on a Bruker Impact II spectrometer featuring a quadrupole time-of-flight (Q-TOF) mass analyzer. Samples were dissolved in methanol.

Melting points were measured on a M5000 melting point meter from A. KRÜSS Optronic GmbH, Germany.

Analytical reverse phase HPLC measurements were performed on a Dionex Ultimate 3000 system equipped with an LPG-3400A pump, a VWD-3100 detector and a Shodex RI-101 refractive index detector. Preparative reverse phase HPLC separations were carried out with a Knauer Azura system equipped with a P2.1L pump, a UVD 2.1L detector and an ASM 2.1L module. Analytical chiral HPLC measurements were performed on a Dionex system equipped with a P680 pump and a UVD 170U detector. Preparative chiral HPLC separations were carried out with a Knauer Azura system equipped with a P6.1L pump, a DAD 2.1L detector and an ASM 2.2L module.

1.2 Synthesis of phthalazines

Phthalazines **1b**-**i**,**k**-**m** were synthesized according to literature.[2–5] Phthalazine **1j** was synthesized using the following route.

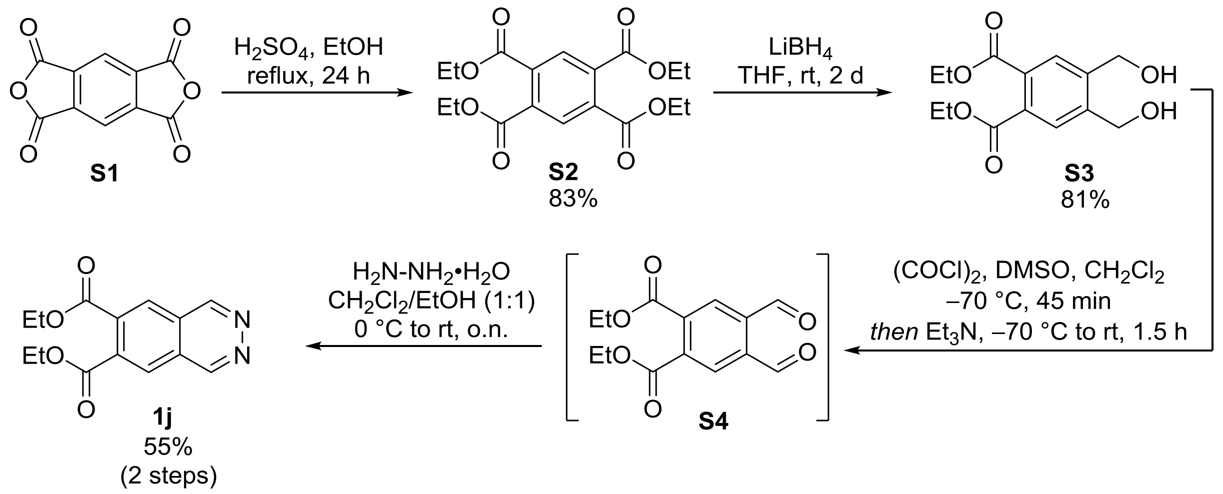

Scheme S1. Synthesis scheme of diethyl phthalazine-6,7-dicarboxylate (**1j**).

Synthesis of tetraethyl benzene-1,2,4,5-tetracarboxylate (S2**)**

The synthesis of tetraethyl benzene-1,2,4,5-tetracarboxylate (**S2**) was adapted from literature with slight modifications.[6]

Pyromellitic dianhydride (**S1**) (5.02 g, 22.3 mmol, 1.00 eq.) was suspended in absolute ethanol (50 mL) and refluxed until it was completely dissolved. H_2SO_4 (3.0 mL, 54 mmol, 2.4 eq.) was added dropwise and the mixture was refluxed for 20 h. Afterwards, more H_2SO_4 (2.0 mL, 36 mmol, 1.6 eq.) was added and it was reflux for further 4 h. Subsequently, 25 mL of ethanol were distilled off under atmospheric pressure and the remaining solution was cooled to rt and poured into ice-cold aq. NaOH solution (2 N, 40 mL). The aqueous layer was extracted with diethyl ether (40 mL). Afterwards, the aqueous layer was basified with aq. NaOH solution (2 N) and extracted with diethyl ether (2 x 30 mL). The combined organic extracts were washed with aq. NaOH solution (1 N, 20 mL) and brine, dried (Na_2SO_4) filtered and concentrated *in vacuo* to give the desired ester **S2** as a colorless oil (6.75 g, 18.4 mmol, 83%) which solidified upon storage at rt over two weeks. It was used in the next step without further purification.

^{1}H NMR (200 MHz, $CDCl_3$): δ = 8.04 (s, 2H), 4.39 (q, J = 7.1 Hz, 8H), 1.38 (t, J = 7.1 Hz, 12H) ppm.

Analytical data corresponds to literature.[6]

Synthesis of diethyl 4,5-bis(hydroxymethyl)benzene-1,2-dicarboxylate (S3**)**

The synthesis of diethyl 4,5-bis(hydroxymethyl)benzene-1,2-dicarboxylate (**S3**) was adapted from literature with slight modifications.[7]

$LiBH_4$ (207 mg, 9.01 mmol, 2.20 eq.) was added to a solution of ester **S2** (1.5 g, 4.1 mmol, 1.0 eq.) in anhydrous THF (17 mL) under nitrogen atmosphere and the mixture was stirred at rt for 2 d. Afterwards, aq. HCl (1 N, 2.7 mL) was added carefully. The resulting precipitate was removed by filtration and washed with CH_2Cl_2. The filtrate was diluted with water (15 mL) and extracted with CH_2Cl_2 (3 x 15 mL). The combined organic extracts were washed with brine, dried ($MgSO_4$), filtered and concentrated *in vacuo*. Purification via flash chromatography [30 g silica, Cy/EA (3:7)] yielded the desired diol **S3** as a colorless oil (933 mg, 3.31 mmol, 81%).

^{1}H NMR (200 MHz, DMSO-d$_6$): δ = 7.72 (s, 2H), 5.50 (t, *J* = 5.5 Hz, 2H), 4.55 (d, *J* = 5.5 Hz, 4H), 4.25 (q, *J* = 7.1 Hz, 4H), 1.26 (t, *J* = 7.1 Hz, 6H) ppm.

Analytical data corresponds to literature.[7]

Synthesis of diethyl phthalazine-6,7-dicarboxylate (1j**)**

A solution of anhydrous DMSO (1.0 mL, 14 mmol, 4.4 eq.) in anhydrous CH_2Cl_2 (4 mL) was added dropwise to a solution of oxalyl chloride (0.63 mL, 7.2 mmol, 2.2 eq.) in anhydrous CH_2Cl_2 (15 mL) at −70 °C under nitrogen atmosphere. After stirring for 15 min at the same temperature, a solution of diol **S3** (0.92 g, 3.3 mmol, 1.0 eq.) in anhydrous THF (4 mL) was added dropwise. The mixture was stirred for further 45 min at −70 °C before anhydrous Et_3N (5.7 mL, 39 mmol, 12 eq.) was added. The resulting suspension was slowly warmed to rt over 1.5 h. Afterwards, water (20 mL) was added and the organic layer was separated. The aqueous layer was extracted with CH_2Cl_2 (2 x 25 mL). The combined organic layers were washed with brine, dried ($MgSO_4$), filtered and concentrated *in vacuo* to give the crude dialdehyde **S4** as yellow oil that was directly used in the next step without further purification.

Dialdehyde **S4** was dissolved in ethanol (10 mL) and CH_2Cl_2 (17 mL) and a solution of hydrazine monohydrate (0.170 mL, 3.42 mmol, 1.05 eq.) in ethanol (7 mL) was added dropwise at 0 °C. The mixture was slowly warmed to rt overnight. Afterwards, the solvent was removed *in vacuo* and the crude product was purified via flash chromatography [50 g silica, Cy/EA (2:8 to 1:9), dry loading (acetone/CH_2Cl_2)]. Phthalazine **1j** was obtained as a yellow solid (489 mg, 1.78 mmol, 55%).

^{1}H NMR (400 MHz, $CDCl_3$): δ = 9.69 (s, 2H), 8.38 (s, 2H), 4.46 (q, *J* = 7.1 Hz, 4H), 1.43 (t, *J* = 7.1 Hz, 6H) ppm.

$^{13}C\{^{1}H\}$ NMR (101 MHz, $CDCl_3$): δ = 166.0 (2C), 151.0 (2C), 136.1 (2C), 128.1 (2C), 126.7 (2C), 62.7 (2C), 14.2 (2C) ppm.

HRMS (ESI): *m/z* calculated for $C_{14}H_{14}N_2O_4$+Na: 297.0846 $[M+Na]^+$, found: 297.0850.

R_f: 0.34 (EtOAc).

Melting Point: 87-88 °C.

1.3 Synthesis of *tert*-butyl azete-1(2*H*)-carboxylate (3)

The synthesis of *tert*-butyl azete-1(2*H*)-carboxylate (**3**) was adopted from the literature with slight modifications:[8,9]

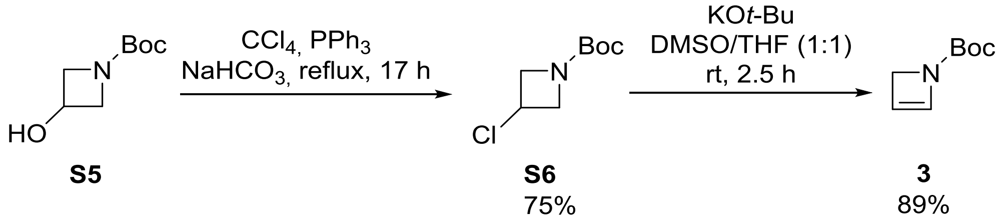

Scheme S2. Synthesis route of *tert*-butyl azete-1(2*H*)-carboxylate (**3**).

Synthesis of *tert*-butyl 3-chloroazetidine-1-carboxylate (S6)

To a solution of alcohol **S5** (4.07 g, 23.0 mmol, 1.00 eq.) in CCl_4 (25 mL) were added PPh_3 (6.70 g, 25.3 mmol, 1.10 eq.) and $NaHCO_3$ (15 mg, 0.18 mmol, 0.78 mol%) and the mixture was stirred under reflux for 17 h. Afterwards, the mixture was cooled to rt and concentrated *in vacuo*. The residue was suspended in pentane (25 mL) and filtered. The filtered cake was washed with pentane (25 mL) and the combined filtrates were concentrated *in vacuo*. The crude product was purified via flash chromatography [150 g silica gel, pentane/Et_2O (100:0 to 80:20)] to obtain the desired chloroazetidine **S6** as a colorless oil (3.30 g, 17.2 mmol, 75%).

^{1}H NMR (200 MHz, $CDCl_3$): δ = 4.61 – 4.44 (m, 1H), 4.44 – 4.31 (m, 2H), 4.10 – 3.97 (m, 2H), 1.44 (s, 9H) ppm.

Analytical data corresponds to literature.[8]

Synthesis of *tert*-butyl azete-1(2*H*)-carboxylate (3)

A solution of KO*t*-Bu (578 mg, 5.00 mmol, 1.47 eq.) in anhydrous DMSO (25 mL) was added dropwise to a solution of chlorazetidine **S6** (652 mg, 3.40 mmol, 1.00 eq.) in anhydrous THF (25 mL) at rt under nitrogen atmosphere. After stirring for 2.5 h at rt, water (20 mL) was added and the mixture was extracted with Et_2O (3 x 30 mL). The combined organic layers were washed with water (3 x 40 mL) and brine (40 mL), dried (Na_2SO_4), filtered and concentrated *in vacuo* to obtain the azetine **3** as a colorless to pale yellow oil (472 mg, 3.04 mmol, 89%) that was used in the next step without further purification.

The azetine **3** was stored at −25 °C in a nitrogen filled glove box.

^{1}H NMR (200 MHz, CD_2Cl_2): δ = 6.57 (dt, *J* = 1.7, 0.8 Hz, 1H), 5.54 (dt, *J* = 1.7, 0.8 Hz, 1H), 4.36 (t, *J* = 0.8 Hz, 2H), 1.44 (s, 9H) ppm.

Analytical data corresponds to literature.[9]

1.4 General procedure for the IEDDA reaction/thermal ring expansion sequence (GP1)

In a nitrogen filled glove box, (substituted) phthalazine (**1**) (0.25 mmol, 1.0 eq.) and **BDLA** catalyst (2.6 mg, 13 µmol, 5.0 mol%) were suspended in anhydrous and degassed 1,4-dioxane or diglyme (4 mL) in a round bottom flask. In a separate flask, azetine **3** (48 mg, 0.31 mmol, 1.2 eq.) was dissolved in anhydrous and degassed 1,4-dioxane or diglyme (1 mL). Both flasks were sealed with a septum and taken out of the glove box. The following reaction steps were performed in the fume hood under Schlenk conditions. The azetine solution was transferred to a 1 mL syringe and added to the phthalazine/**BDLA** mixture with a syringe pump over 20 h at the indicated temperature. After the complete addition, the mixture was stirred for further 2 h at the same temperature. Afterwards, the mixture was concentrated *in vacuo*. If the reaction was performed in diglyme, the residue was diluted with cyclohexane (10 mL), washed with water (4 x 1 mL) and brine (2 mL), dried (Na_2SO_4), filtered and concentrated *in vacuo*. The crude products were purified via automated flash chromatography using the conditions given below.

1.4.1 Synthesis of *tert*-butyl (1*Z*,5*Z*)-benzo[*d*]azocine-3(4*H*)-carboxylate (5a)

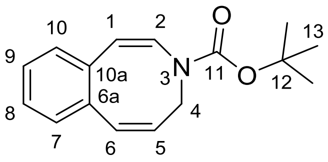

According to **GP1**, azetine **3** (186 mg, 1.20 mmol, 1.20 eq.) in diglyme (4 mL) was added to a mixture of phthalazine (**1a**) (133 mg, 1.00 mmol, 1.0 eq.) and **BDLA** catalyst (6.1 mg, 30 µmol, 3.0 mol%) in diglyme (16 mL) at 110 °C. Purification via automated flash chromatography [12 g silica gel, cyclohexane/EtOAc (100:0 to 99:1)] yielded azocine **5a** as a colorless oil (188 mg, 0.731 mmol, 73%).

^{1}H NMR (400 MHz, CD_3CN): δ = 7.25 (td, *J* = 7.5, 1.5 Hz, 1H, H-9), 7.18 (td, *J* = 7.5, 1.5 Hz, 1H, H-8), 7.15 – 7.10 (m, 1H, H-10), 7.08 – 7.00 (m, 1H, H-7), 6.94 (d, *J* = 10.4 Hz, 1H, H-6), 6.87 (d, *J* = 11.4 Hz, 1H, H-2), 6.10 (dt, *J* = 10.4, 8.4 Hz, 1H, H-5), 5.38 (s, br, 1H, H-1), 4.31 (d, *J* = 8.4 Hz, 2H, H-4), 1.48 (s, 9H, H-13) ppm.

^{13}C{^{1}H} NMR (101 MHz, CD_3CN): δ = 153.7 (br, C-11), 137.7 (br, C-6), 136.6 (C-10a), 135.2 (C-6a), 132.2 (C-10), 130.2 (C-7), 129.2 (C-9), 128.6 (br, C-5), 127.4 (C-8), 127.3 (C-2), 107.7 (br, C-1), 82.3 (C-12), 42.7 and 41.6 (br, C-4), 28.3 (3C, C-13) ppm. Due to conformational changes, C-4 shows two broad signals with low intensity (compare Figure S9 and Figure S10).

HRMS (ESI): *m/z* calculated for $C_{16}H_{19}NO_2$+Na: 280.1308 $[M+Na]^+$, found: 280.1308.

R_f: 0.39 (Cy/EtOAc, 95:5).

1.4.2 Synthesis of *tert*-butyl (1*Z*,5*Z*)-7,10-difluorobenzo[*d*]azocine-3(4*H*)-carboxylate (5b)

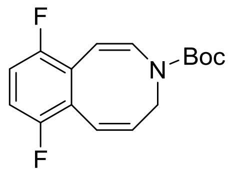

According to **GP1**, azetine **3** (48 mg, 0.31 mmol, 1.2 eq.) was added to a mixture of phthalazine **1b** (42 mg, 0.25 mmol, 1.0 eq.) and **BDLA** catalyst (2.6 mg, 13 µmol, 5.0 mol%) in 1,4-dioxane at 80 °C. Purification via automated flash chromatography [12 g silica gel, cyclohexane/EtOAc (100:0 to 90:10), dry loading on celite with CH_2Cl_2] yielded azocine **5b** as a colorless to pale yellow oil (63 mg, 0.22 mmol, 87%).

^{1}H NMR (400 MHz, CD_3CN): δ = 7.13 – 7.02 (m, 2H), 6.96 (td, *J* = 9.0, 4.4 Hz, 1H), 6.88 (dd, *J* = 10.4, 3.0 Hz, 1H), 6.25 (dt, *J* = 10.4, 8.4 Hz, 1H), 5.24 (d, *J* = 11.5 Hz, 1H), 4.41 (d, *J* = 8.4 Hz, 2H), 1.48 (s, 9H) ppm.

^{13}C{^{1}H} NMR (101 MHz, CD_3CN): δ = 158.7 (dd, *J* = 79.0, 2.3 Hz), 156.3 (dd, br, *J* = 78.2, 2.4 Hz), 153.5, 131.4, 130.4 (d, *J* = 2.1 Hz), 130.1 (br), 126.6 (dd, *J* = 14.9, 3.4 Hz), 125.1 (dd, *J* = 18.2, 2.1 Hz), 116.7 (dd, *J* = 26.5, 9.5 Hz), 115.0 (dd, *J* = 25.2, 9.5 Hz), 98.7, 82.8, 41.8 (br), 28.3 (3C) ppm.

^{19}F{^{1}H} NMR (377 MHz, CD_3CN): δ = -118.27 (br), -120.64 (d, *J* = 16.4 Hz) ppm.

HRMS (ESI): *m/z* calculated for $C_{16}H_{17}F_2NO_2$+Na: 316.1120 $[M+Na]^+$, found: 316.1121.

R_f: 0.22 (Cy/EtOAc, 97:3).

1.4.3 Synthesis of *tert*-butyl (1*Z*,5*Z*)-7,10-dichlorobenzo[*d*]azocine-3(4*H*)-carboxylate (5c)

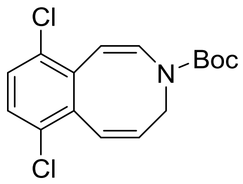

According to **GP1**, azetine **3** (48 mg, 0.31 mmol, 1.2 eq.) was added to a mixture of phthalazine **1c** (50 mg, 0.25 mmol, 1.0 eq.) and **BDLA** catalyst (2.6 mg, 13 µmol, 5.0 mol%) in 1,4-dioxane at 80 °C. Purification via automated flash chromatography [12 g silica gel, cyclohexane/EtOAc (100:0 to 99:1), dry loading on celite with CH_2Cl_2] yielded azocine **5c** as a colorless to pale yellow oil (54 mg, 0.17 mmol, 66%).

^{1}H NMR (400 MHz, CD_3CN): δ = 7.40 (d, *J* = 8.6 Hz, 1H), 7.28 (d, *J* = 8.6 Hz, 1H), 7.09 – 6.88 (m, 2H), 6.15 (td, *J* = 10.3, 7.7 Hz, 1H), 5.29 (d, *J* = 11.4 Hz, 1H), 4.55 (s, br, 1H), 4.06 (s, br, 1H), 1.48 (s, 9H) ppm.

^{13}C{^{1}H} NMR (101 MHz, CD_3CN): δ = 137.1, 135.9, 134.8 (br), 134.1, 132.8, 131.1, 129.4, 129.3 (br), 129.2, 105.3 (br), 82.8, 28.3 (3C) ppm. Due to peak broadening caused by conformational changes, two signals cannot be detected (compare Figure S9 and Figure S10).

HRMS (ESI): *m/z* calculated for $C_{16}H_{17}Cl_2NO_2$+Na: 348.0529 $[M+Na]^+$, found: 348.0529.

R_f: 0.44 (Cy/EtOAc, 95:5).

1.4.4 Synthesis of *tert*-butyl (1*Z*,5*Z*)-10-nitrobenzo[*d*]azocine-3(4*H*)-carboxylate (5da) and *tert*-butyl (1*Z*,5*Z*)-7-nitrobenzo[*d*]azocine-3(4*H*)-carboxylate (5db)

According to **GP1**, azetine **3** (48 mg, 0.31 mmol, 1.2 eq.) was added to a mixture of phthalazine **1d** (44 mg, 0.25 mmol, 1.0 eq.) and **BDLA** catalyst (2.6 mg, 13 µmol, 5.0 mol%) in 1,4-dioxane at 80 °C. Purification via automated flash chromatography [12 g silica gel, cyclohexane/EtOAc (99:1 to 89:11), dry loading on celite with CH_2Cl_2] yielded azocine **5db** as a yellow oil (36 mg, 0.12 mmol, 47%) and azocine **5da** as a yellow oil (32 mg, 0.11 mmol, 42%).

Tert-butyl (1*Z*,5*Z*)-10-nitrobenzo[*d*]azocine-3(4*H*)-carboxylate (**5da**)

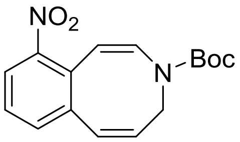

^{1}H NMR (400 MHz, CD_2Cl_2): δ = 7.66 (dd, *J* = 7.9, 1.4 Hz, 1H), 7.31 (t, *J* = 7.9 Hz, 1H), 7.25 (d, *J* = 7.9 Hz, 1H), 7.13 – 6.88 (m, 2H), 6.17 (dt, *J* = 10.3, 8.4 Hz, 1H), 5.26 (d, *J* = 11.2 Hz, 1H), 4.37 (s, br, 2H), 1.49 (s, 9H) ppm.

^{13}C{^{1}H} NMR (101 MHz, CD_2Cl_2): δ = 152.9 (br), 151.6, 138.3, 135.2, 132.9, 129.8 (br), 129.7 (br), 129.4, 127.2, 123.7, 100.8 (br), 82.4, 40.9 (br), 28.3 (3C) ppm.

HRMS (ESI): *m/z* calculated for $C_{16}H_{18}N_2O_4$+Na: 325.1159 [M+Na]$^+$, found: 325.1156.

R$_f$: 0.26 (Cy/EtOAc, 9:1).

The position of the nitro group was assigned via NOESY (see Figure S15).

Tert-butyl (1*Z*,5*Z*)-7-nitrobenzo[*d*]azocine-3(4*H*)-carboxylate (**5db**)

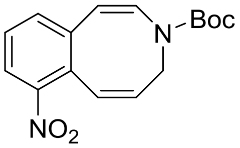

^{1}H NMR (400 MHz, CD_2Cl_2): δ = 7.78 (dd, *J* = 7.4, 2.0 Hz, 1H), 7.45 – 7.34 (m, 2H), 7.11 (d, *J* = 10.6 Hz, 1H), 6.92 (s, br, 1H), 6.17 (dt, *J* = 10.6, 8.4 Hz, 1H), 5.39 (s, br, 1H), 4.36 (s, br, 2H), 1.49 (s, 9H) ppm.

^{13}C NMR{^{1}H} (101 MHz, CD_2Cl_2): δ = 152.9 (br), 149.6, 140.1, 136.0, 132.4 (br), 129.7, 129.1, 128.7, 128.6, 122.8, 105.5 (br), 82.3, 42.5 and 41.4 (br, 1C), 28.3 (3C) ppm.

HRMS (ESI): *m/z* calculated for $C_{16}H_{18}N_2O_4$+Na: 325.1159 [M+Na]$^+$, found: 325.1155.

R$_f$: 0.33 (Cy/EtOAc, 9:1).

1.4.5 Synthesis of *tert*-butyl (5*Z*,9*Z*)-pyrido[2,3-*d*]azocine-8(7*H*)-carboxylate (5ea) and *tert*-butyl (5*Z*,9*Z*)-pyrido[3,2-*d*]azocine-7(8*H*)-carboxylate (5eb)

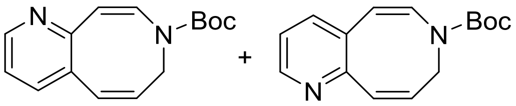

According to **GP1**, azetine **3** (48 mg, 0.31 mmol, 1.2 eq.) was added to a mixture of pyridazine **1e** (33 mg, 0.25 mmol, 1.0 eq.) and **BDLA** catalyst (2.6 mg, 13 µmol, 5.0 mol%) in 1,4-dioxane at 80 °C. Purification via automated flash chromatography [12 g silica gel, cyclohexane/EtOAc (88:12 to 25:75), dry loading on celite with CH_2Cl_2] yielded a mixture of azocines **5ea** and **5eb** as a yellow oil (46 mg, 0.18 mmol, 71%, regioisomeric ratio: 81:19).

^{1}H NMR (400 MHz, CD_3CN): δ = 8.45 (dd, *J* = 4.7, 1.8 Hz, 1H, minor isomer), 8.38 (dd, *J* = 4.7, 1.7 Hz, 1H, major isomer), 7.46 (dd, *J* = 7.9, 1.7 Hz, 1H, major isomer), 7.39 (dd, *J* = 7.8, 1.7 Hz, 1H, minor isomer), 7.20 (dd, *J* = 7.9, 4.6 Hz, 1H, major isomer), 7.13 (dd, *J* = 7.7, 4.7 Hz, 1H, minor isomer), 7.05 – 6.76 (m, 4H, major and minor isomer), 6.28 – 6.11 (m, 2H, major and minor isomer), 5.45 (s, br, 1H, minor isomer), 5.29 (s, br, 1H, major isomer), 4.38 – 4.30 (m, 4H, major and minor isomer), 1.49 and 1.48 (two overlapping s, 18H, major and minor isomer) ppm.

^{13}C{^{1}H} NMR (101 MHz, CD_3CN): δ = 154.4, 153.5, 150.1, 148.1, 139.6, 138.2 (br), 137.8, 135.4 (br), 132.6, 130.9, 129.5 (br), 128.5, 128.2, 123.7, 121.9, 105.1 (br), 82.6 (br), 41.7 (br), 28.3 ppm.

NMR spectra could not be assigned unambiguously.

HRMS (ESI): *m/z* calculated for $C_{15}H_{18}N_2O_2$+Na: 281.1260 [M+Na]$^+$, found: 281.1262.

R_f: 0.35 (Cy/EtOAc, 1:1).

1.4.6 Synthesis of *tert*-butyl (1*Z*,5*Z*)-10-fluorobenzo[*d*]azocine-3(4*H*)-carboxylate (5fa) and *tert*-butyl (1*Z*,5*Z*)-7-fluorobenzo[*d*]azocine-3(4*H*)-carboxylate (5fb)

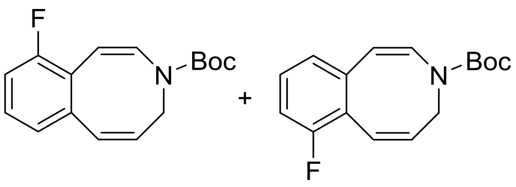

According to **GP1**, azetine **3** (48 mg, 0.31 mmol, 1.2 eq.) was added to a mixture of phthalazine **1f** (37 mg, 0.25 mmol, 1.00 eq.) and **BDLA** catalyst (2.6 mg, 13 µmol, 5.0 mol%) in 1,4-dioxane at 90 °C. Purification via automated flash chromatography [12 g silica gel, cyclohexane/EtOAc (99:1 to 88:12), dry loading on celite with CH_2Cl_2] yielded a mixture of azocines **5fa** and **5fb** as a colorless oil (60 mg, 0.22 mmol, 88%, regioisomeric ratio: 51:49).

^{1}H NMR (400 MHz, CD_3CN): δ = 7.32 – 7.16 (m, 2H), 7.08 – 6.98 (m, 2H), 6.99 – 6.70 (m, 6H), 6.21 (dt, *J* = 10.4, 8.3 Hz, 1H), 6.13 (dt, *J* = 10.4, 8.4 Hz, 1H), 5.38 (s, br, 1H), 5.29 (s, br, 1H), 4.36 and 4.35 (two overlapping d, *J* = 8.4 Hz, 4H), 1.482 and 1.480 (two overlapping s, 18H) ppm.

^{13}C{^{1}H} NMR (101 MHz, CD_3CN): δ = 163.3, 162.6, 160.8, 160.2, 153.6 (br), 139.6, 139.5, 138.1, 138.1, 136.6 (br), 130.9, 130.8, 130.8, 130.5 (br), 129.6, 129.4 (br), 128.9, 128.8, 128.2, 127.9, 127.9, 125.9, 125.8, 124.7, 124.6, 123.3, 123.1, 115.5, 115.3, 113.9, 113.7, 106.5 (br), 99.5 (br), 82.5, 41.7 (br), 28.3 ppm.

^{19}F{^{1}H} NMR (377 MHz, CD_3CN): δ = -113.47, -115.85 ppm.

NMR spectra could not be assigned unambiguously.

HRMS (ESI): *m/z* calculated for $C_{16}H_{18}FNO_2$+Na: 298.1214 [M+Na]$^+$, found: 298.1215.

R_f: 0.44 (Cy/EtOAc, 9:1).

1.4.7 Synthesis of *tert*-butyl (1*Z*,5*Z*)-9-fluorobenzo[*d*]azocine-3(4*H*)-carboxylate and *tert*-butyl (1*Z*,5*Z*)-8-fluorobenzo[*d*]azocine-3(4*H*)-carboxylate (5g)

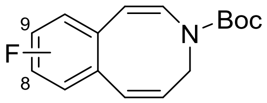

According to **GP1**, azetine **3** (48 mg, 0.31 mmol, 1.2 eq.) was added to a mixture of phthalazine **1g** (37 mg, 0.25 mmol, 1.00 eq.) and **BDLA** catalyst (2.6 mg, 13 µmol, 5.0 mol%) in 1,4-dioxane at 90 °C. Purification via automated flash chromatography [12 g silica gel, cyclohexane/EtOAc (100:0 to 95:5), dry loading on celite with CH_2Cl_2] yielded a mixture of azocines **5g** as a colorless oil (42 mg, 0.17 mmol, 67%, regioisomeric ratio: 52:48).

^{1}H NMR (400 MHz, CD_3CN): δ = 7.18 – 7.09 (m, 1H), 7.07 – 6.97 (m, 2H), 6.97 – 6.76 (m, 7H), 6.23 – 6.00 (m, 2H), 5.32 (s, br, 2H), 4.31 (d, *J* = 8.4 Hz, 4H), 1.48 and 1.47 (two overlapping s, 18H) ppm.

^{13}C{^{1}H} NMR (101 MHz, CD_3CN): δ = 164.9, 163.6, 162.5, 161.2, 153.6 (br), 139.2, 139.2, 137.6, 137.5, 136.7 (br), 134.1, 134.0, 133.0, 132.9, 132.1, 132.0, 131.6, 131.6, 129.7 (br), 128.8 (br), 128.3, 127.3, 118.0, 116.4, 116.2, 116.1, 115.9, 114.3, 114.1, 106.6 (br), 82.5 (br), 82.4 (br), 42.6 (br), 41.6 (br), 28.3, 28.3 ppm.

^{19}F{^{1}H} NMR (377 MHz, CD_3CN): δ = -117.17, -118.83 ppm.

NMR spectra could not be assigned unambiguously.

HRMS (ESI): *m/z* calculated for $C_{16}H_{18}FNO_2$+Na: 298.1214 [M+Na]$^+$, found: 298.1216.

R_f: 0.37 (Cy/EtOAc, 95:5).

1.4.8 Synthesis of *tert*-butyl (1*Z*,5*Z*)-8-chlorobenzo[*d*]azocine-3(4*H*)-carboxylate and *tert*-butyl (1*Z*,5*Z*)-9-chlorobenzo[*d*]azocine-3(4*H*)-carboxylate (5h)

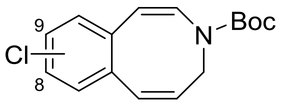

According to **GP1**, azetine **3** (48 mg, 0.31 mmol, 1.2 eq.) was added to a mixture of phthalazine **1h** (41 mg, 0.25 mmol, 1.0 eq.) and **BDLA** catalyst (2.6 mg, 13 µmol, 5.0 mol%) in 1,4-dioxane at 90 °C. Purification via automated flash chromatography [12 g silica gel, cyclohexane/EtOAc (99:1 to 93:7), dry loading on celite with CH_2Cl_2] yielded a mixture of azocines **5h** as a colorless to pale yellow oil (54 mg, 0.18 mmol, 74%, regioisomeric ratio: 53:47).

^{1}H NMR (400 MHz, CD_3CN): δ = 7.24 (dd, *J* = 8.4, 2.4 Hz, 1H), 7.21 – 7.13 (m, 2H), 7.09 (d, *J* = 8.4 Hz, 1H), 7.07 (d, *J* = 2.3 Hz, 1H), 7.01 (d, *J* = 8.3 Hz, 1H), 6.95 – 6.84 (m, 4H), 6.20 – 6.06 (m, 2H), 5.31 (s, 2H), 4.31 (d, *J* = 8.4 Hz, 4H), 1.48 and 1.47 (two overlapping s, 18H) ppm.

^{13}C{^{1}H} NMR (101 MHz, CD_3CN): δ = 153.6 (br), 138.8, 137.3, 136.6 (br), 136.5 (br), 135.6, 134.4, 134.1, 133.8, 132.6, 131.7, 131.6, 129.8 (br), 129.7, 129.3 (br), 129.0, 128.5, 128.0, 127.2, 106.4 (br), 82.5, 42.6 (br), 41.5 (br), 28.3 ppm.

NMR spectra could not be assigned unambiguously.

HRMS (ESI): *m/z* calculated for $C_{16}H_{18}ClNO_2$+Na: 314.0918 [M+Na]$^+$, found: 314.0918.

R_f: 0.34 (Cy/EtOAc, 95:5).

1.4.9 Synthesis of *tert*-butyl (1*Z*,5*Z*)-8-(trifluoromethyl)benzo[*d*]azocine-3(4*H*)-carboxylate and *tert*-butyl (1*Z*,5*Z*)-9-(trifluoromethyl)-benzo[*d*]azocine-3(4*H*)-carboxylate (5i)

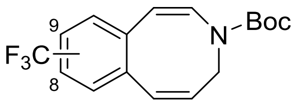

According to **GP1**, azetine **3** (48 mg, 0.31 mmol, 1.2 eq.) was added to a mixture of phthalazine **1i** (50 mg, 0.25 mmol, 1.0 eq.) and **BDLA** catalyst (2.6 mg, 13 µmol, 5.0 mol%) in 1,4-dioxane at 90 °C. Purification via automated flash chromatography [12 g silica gel, cyclohexane/EtOAc (98:2 to 96:4), dry loading on celite with CH_2Cl_2] yielded a mixture of azocines **5i** as a colorless oil (65 mg, 0.20 mmol, 79%, regioisomeric ratio: 54:46) that solidified upon storage at 2 °C.

^{1}H NMR (400 MHz, CD_3CN): δ = 7.53 (dd, *J* = 8.3, 2.1 Hz, 1H), 7.46 (dd, *J* = 8.3, 2.0 Hz, 1H), 7.43 (s, 1H), 7.35 (s, 1H), 7.29 (d, *J* = 8.2 Hz, 1H), 7.21 (d, *J* = 8.2 Hz, 1H), 7.05 – 6.87 (m, 4H), 6.29 – 6.28 (m, 2H), 5.39 (d, *J* = 11.6 Hz, 2H), 4.33 (d, *J* = 8.4 Hz, 4H), 1.48 (s, 18H) ppm.

^{13}C{^{1}H} NMR (101 MHz, CD_3CN): δ = 153.6 (br), 141.0, 139.4, 137.8, 136.5 (br), 136.2, 132.9, 131.0, 130.7, 130.4, 130.1 (br), 130.0 (br), 129.0, 128.8 (br), 128.7, 128.5, 127.0 (br), 126.8, 126.7, 125.6 (br), 124.1, 124.0, 123.8 (br), 106.3 (br), 82.7, 41.6 (br), 28.3 ppm.

^{19}F{^{1}H} NMR (377 MHz, CD_3CN): δ = -63.11, -63.27 ppm.

NMR spectra could not be assigned unambiguously.

HRMS (ESI): *m/z* calculated for $C_{17}H_{18}F_3NO_2$+Na: 348.1182 [M+Na]$^+$, found: 348.1181.

R_f: 0.44 (Cy/EtOAc, 9:1).

Melting Point: 77-78 °C.

1.4.10 Synthesis of 3-(*tert*-butyl) 8,9-diethyl (1*Z*,5*Z*)-benzo[*d*]azo-cine-3,8,9(4*H*)-tricarboxylate (5j)

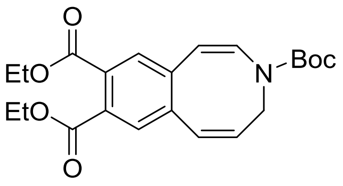

According to **GP1**, azetine **3** (33 mg, 0.21 mmol, 1.2 eq.) was added to a mixture of phthalazine **1j** (48 mg, 0.18 mmol, 1.0 eq.) and **BDLA** catalyst (1.8 mg, 9.0 µmol, 5.0 mol%) in 1,4-dioxane at 80 °C. Purification via automated flash chromatography [12 g silica gel, cyclohexane/EtOAc (96:4 to 74:26), dry loading on celite with CH_2Cl_2] yielded azocine **5j** as a colorless to pale yellow oil (50 mg, 0.13 mmol, 72%).

^{1}H NMR (400 MHz, CD_3CN): δ = 7.41 (s, 1H), 7.39 (s, 1H), 7.08 – 6.88 (m, 2H), 6.22 (dt, *J* = 10.4, 8.4 Hz, 1H), 5.36 (d, *J* = 11.4 Hz, 1H), 4.34 (d, *J* = 8.4 Hz, 2H), 4.32 – 4.24 (m, 4H), 1.48 (s, 9H), 1.37 – 1.22 (m, 6H) ppm.

^{13}C{^{1}H} NMR (101 MHz, CD_3CN): δ = 168.2, 167.8, 153.5 (br), 140.2, 138.1, 136.2 (br), 133.2, 132.4, 130.8, 130.6 (br), 130.5, 129.4, 105.9 (br), 82.8, 62.5, 62.4, 41.5 (br), 28.3 (3C), 14.4 (2C) ppm.

HRMS (ESI): *m/z* calculated for $C_{22}H_{27}NO_6$+Na: 424.1730 $[M+Na]^+$, found: 424.1730.

R_f: 0.34 (Cy/EtOAc, 8:2).

1.4.11 Synthesis of *tert*-butyl (1*Z*,5*Z*)-naphtho[2,3-*d*]azocine-3(4*H*)-carboxylate (5k)

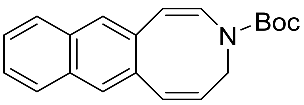

According to **GP1**, azetine **3** (47 mg, 0.31 mmol, 1.2 eq.) was added to a mixture of phthalazine **1k** (45 mg, 0.25 mmol, 1.0 eq.) and **BDLA** catalyst (2.6 mg, 13 µmol, 5.0 mol%) in diglyme at 110 °C. Purification via flash chromatography [12 g silica gel, cyclohexane/EtOAc (100:0 to 97.5:2.5)] followed by a second flash chromatography [8 g silica gel, cyclohexane/EtOAc (100:0 to 97:3)] yielded azocine **5k** as a colorless oil (54 mg, 0.18 mmol, 71%) that solidified slowly upon storage at room temperature.

^{1}H NMR (400 MHz, CD_2Cl_2): δ = 7.78 – 7.68 (m, 2H), 7.60 (s, 1H), 7.53 (s, 1H), 7.46 – 7.35 (m, 2H), 7.10 (d, *J* = 10.3 Hz, 1H), 6.92 (b, 1H), 6.14 – 6.02 (m, 1H), 5.57 (s, br, 1H), 4.34 (d, *J* = 8.5 Hz, 2H), 1.51 (s, 9H) ppm.

^{13}C{^{1}H} NMR (101 MHz, CD_2Cl_2): δ = 136.4 (br), 135.0, 133.9, 133.7, 132.3, 130.1 (br), 128.5, 127.7, 127.5, 127.1, 126.6, 126.5, 126.2, 106.5 (br), 81.9 (br), 41.1 (br), 28.3 (3C) ppm.

Due to peak broadening caused by conformational changes, one signal can't be detected (compare Figure S9 and Figure S10).

HRMS (ESI): *m/z* calculated for $C_{20}H_{21}NO_2$+Na: 330.1464 $[M+Na]^+$, found: 330.1457.

R_f: 0.32 (Cy/EtOAc, 95:5).

Melting Point: 157-158 °C.

1.4.12 Synthesis of *tert*-butyl (1*Z*,5*Z*)-9-methylbenzo[*d*]azocine-3(4*H*)-carboxylate and *tert*-butyl (1*Z*,5*Z*)-8-methylbenzo[*d*]azocine-3(4*H*)-carboxylate (5l)

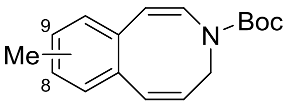

According to **GP1**, azetine **3** (48 mg, 0.31 mmol, 1.2 eq.) was added to a mixture of phthalazine **1l** (36 mg, 0.25 mmol, 1.00 eq.) and **BDLA** catalyst (2.6 mg, 13 µmol, 5.0 mol%) in diglyme at 125 °C. Purification via automated flash chromatography [12 g silica gel, cyclohexane/EtOAc (98:2 to 97:3), dry loading on celite with CH_2Cl_2] yielded a mixture of azocines **5l** as a colorless oil (15 mg, 0.055 mmol, 22%, regioisomeric ratio: 53:47).

^{1}H NMR (400 MHz, CD_3CN): δ = 7.07 (dd, *J* = 8.0, 1.2 Hz, 1H), 7.05 – 6.97 (m, 2H), 6.98 – 6.74 (m, 7H), 6.22 – 5.95 (m, 2H), 5.34 (s, br, 2H), 4.30 and 4.29 (two overlapping d, *J* = 8.4 Hz, 4H), 2.28 and 2.27 (two overlapping s, 6H), 1.48 and 1.47 (two overlapping s, 18H) ppm.

^{13}C NMR{^{1}H} (101 MHz, CD_3CN): δ = 139.0, 137.7 (br), 137.2, 136.4, 135.1, 133.7, 132.8, 132.4, 132.3, 130.7, 130.2, 129.9, 128.6 (br), 128.2, 127.3, 126.9, 107.8 (br), 82.2 (br), 42.8 (br), 41.6 (br), 28.3, 21.0, 20.8 ppm.

NMR spectra could not be assigned unambiguously. Due to peak broadening caused by conformational changes, some ^{13}C signals can't be detected (compare Figure S9 and Figure S10).

HRMS (ESI): *m/z* calculated for $C_{17}H_{21}NO_2$+Na: 294.1464 $[M+Na]^+$, found: 294.1464.

R_f: 0.41 (Cy/EtOAc, 9:1).

1.4.13 Synthesis of *tert*-butyl (1*Z*,5*Z*)-9-methoxybenzo[*d*]azocine-3(4*H*)-carboxylate and *tert*-butyl (1*Z*,5*Z*)-8-methoxybenzo[*d*]azocine-3(4*H*)-carboxylate (5m)

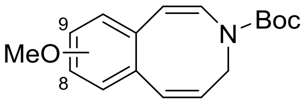

According to **GP1**, azetine **3** (48 mg, 0.31 mmol, 1.2 eq.) was added to a mixture of phthalazine **1m** (40 mg, 0.25 mmol, 1.00 eq.) and **BDLA** catalyst (2.6 mg, 13 µmol, 5.0 mol%) in diglyme at 140 °C. Purification via automated flash chromatography [12 g silica gel, cyclohexane/EtOAc (98:2 to 94:6), dry loading on celite with CH_2Cl_2] yielded a mixture of azocines **5m** as a colorless oil (12 mg, 0.042 mmol, 17%, regioisomeric ratio: 53:47).

^{1}H NMR (400 MHz, CD_3CN): δ = 7.05 (d, *J* = 8.6 Hz, 1H), 6.97 (d, *J* = 8.4 Hz, 1H), 6.92 – 6.73 (m, 6H), 6.69 (d, *J* = 2.7 Hz, 1H), 6.61 (d, *J* = 2.8 Hz, 1H), 6.21 – 5.96 (m, 2H), 5.33 (s, br, 2H), 4.31 and 4.30 (two overlapping d, *J* = 8.3 Hz, 4H), 3.76 (s, 3H), 3.75 (s, 3H), 1.48 and 1.47 (two overlapping s, 18H) ppm.

^{13}C{^{1}H} NMR (101 MHz, CD_3CN): δ = 160.7, 159.4, 138.0, 137.5 (br), 136.6, 133.6, 131.5, 128.9, 127.8, 127.6, 126.3, 117.0, 115.1, 115.0 (br), 113.3, 107.5 (br), 82.2 (br), 55.9, 55.9, 28.3 ppm.

NMR spectra could not be assigned unambiguously. Due to peak broadening caused by conformational changes, some ^{13}C signals can't be detected (compare Figure S9 and Figure S10).

HRMS (ESI): *m/z* calculated for $C_{17}H_{21}NO_3$+Na: 310.1414 $[M+Na]^+$, found: 310.1415.

R_f: 0.51 (Cy/EtOAc, 8:2).

1.4.14 Synthesis of di-*tert*-butyl (2a*R*,3*s*,8*s*,8a*S*,9*S*,10*R*)-4,7-difluoro-2a,3,8,8a-tetrahydro-8,3-[2,3]epazetonaphtho[2,3-*b*]azete-1,11(2*H*)-dicarboxylate (6) and regio-/stereoisomers

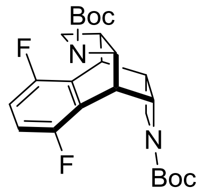

6 (and regio-/stereoisomers)

In a nitrogen filled glove box, phthalazine **1b** (17 mg, 0.10 mmol, 1.0 eq.) and **BDLA** catalyst (1 mg, 5 µmol, 5 mol%) were suspended in anhydrous and degassed 1,4-dioxane (1 mL) in a 4 mL-screw cap vial. Azetine **3** (48 mg, 0.31 mmol, 3.0 eq.) was added, the vial was sealed and taken out of the glove box. The mixture was stirred at 80 °C overnight. Afterwards, the solvent was removed *in vacuo* and the residue was purified via automated flash chromatography [12 g silica gel, cyclohexane/EtOAc (94:6 to 27:73), dry loading on celite with CH_2Cl_2]. A mixture of double Diels-Alder adducts containing the *meso* compound **6** was obtained as a highly viscous, pale yellow oil (43 mg, 0.096 mmol, 93%).

^{1}H NMR (400 MHz, CD_3CN, mixture of regio- and stereoisomers): δ = 7.28 – 6.82 (m, 2H), 4.63 – 3.51 (m, 6H), 2.85 – 2.75 (m, 2H), 2.75 – 2.49 (m, 2H), 1.63 – 0.98 (m, 18H) ppm.

^{13}C{^{1}H} NMR (101 MHz, CD_3CN, mixture of regio- and stereoisomers): δ = 159.3 – 158.4 (m), 156.7 – 156.2 (m), 155.4, 155.1 – 154.5 (m), 127.6, 127.5, 127.4, 127.3, 127.1 – 126.5 (m), 126.4 – 125.8 (m), 115.9 – 114.0 (m), 79.8, 79.6, 62.0, 61.5, 60.5, 60.4, 59.9, 53.4, 52.9, 51.6, 51.0, 35.6, 35.3, 34.2, 33.8, 32.6, 30.7, 30.4, 28.4, 28.2, 27.9 ppm.

^{19}F{^{1}H} NMR (377 MHz, CD_3CN, mixture of regio- and stereoisomers): δ = -128.32 (d, *J* = 21.1 Hz), -128.82 (d, *J* = 21.4 Hz), -128.93 (d, *J* = 21.1 Hz), -129.85 to -130.01 (m), -130.16 to -130.37 (m), -130.67 (d, *J* = 21.1 Hz), -131.02 (d, *J* = 21.1 Hz), -131.37 (d, *J* = 21.4 Hz) ppm.

NMR spectra could not be assigned unambiguously.

HRMS (ESI): *m/z* calculated for $C_{24}H_{30}F_2N_2O_4$+Na: 471.2066 $[M+Na]^+$, found: 471.2069.

R_f: 0.40 (Cy/EtOAc, 1:1).

The obtained mixture of isomers was further purified via preparative HPLC and the structure of the *meso* compound **6** was further verified via X-ray crystallography. (See sections 1.5 and 3).

1.4.15 Synthesis of di-*tert*-butyl 2,2'-oxy(2*S*,2'*S*,5*Z*,5'*Z*)-bis(1,4-dihydrobenzo[*d*]azocine-3(2*H*)-carboxylate) (7)

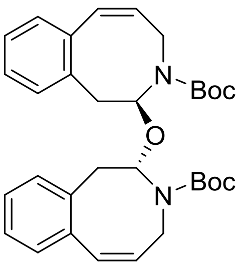

The hemiaminal ether **7** was formed quantitatively as a mixture of stereoisomers from an NMR sample of azocine **5a** in unstabilized $CDCl_3$ by keeping the solution at rt for several days. Evaporation of the solvent yielded a white solid. All attempts to obtain single crystals suitable for X-ray diffraction measurements from the racemic mixture were unsuccessful. Separation of the enantiomers via preparative chiral HPLC (see section 1.5) yielded **7** as a white crystalline solid. The enantioenriched product showed higher crystallinity compared to the racemic mixture, allowing the structure of hemiaminal ether **7** to be further confirmed via single crystal X-ray crystallography (See section 3).

^{1}H NMR (400 MHz, CD_2Cl_2): δ = 7.25 – 7.08 (m, 8H), 6.45 – 6.36 (m, 2H), 5.76 – 5.62 (m, 2H), 5.45 – 5.25 (m, 2H, overlying with residual solvent signal), 4.42 – 4.18 (m, 2H), 4.12 – 3.95 (m, 2H), 3.19 – 3.02 (m, 2H), 2.91 – 2.78 (m, 2H), 1.23 – 0.84 (m, 18H) ppm.

^{13}C{^{1}H} NMR (101 MHz, CD_2Cl_2): δ = 154.3, 154.0, 137.4, 136.8, 134.8, 134.6, 134.5, 131.0, 130.9, 130.8, 130.7, 130.5, 130.4, 130.2, 130.2, 130.0, 129.9, 129.9, 127.5, 127.4, 127.4, 127.3, 127.2, 127.1, 127.0, 126.8, 126.7, 82.8, 81.8, 80.9, 80.0, 79.9, 79.6, 42.0, 41.6, 41.3, 41.0, 39.9, 39.4, 39.1, 28.5, 28.3, 27.9, 27.6 ppm.

NMR spectra could not be assigned unambiguously.

HRMS (ESI): *m/z* calculated for $C_{32}H_{40}N_2O_5$+Na: 555.2829 $[M+Na]^+$, found: 555.2833.

R_f: 0.36 (Cy/EtOAc, 8:2).

Melting Point: 185-186 °C (partial decomposition).

The structure of the hemiaminal ether **7** was further verified via X-ray crystallography. (See section 3).

1.5 Structure elucidation and purification of side products

1.5.1 Double Diels-Alder adducts

To further elucidate the structure of the side products formed in the IEDDA/thermal ring expansion sequence, the mixture of double Diels-Alder adducts (**6** and isomers) obtained from the reaction of phthalazine **1b** with an excess of azetine **3** (see section 1.4.14) was analyzed via HPLC (Figure S1) and the two peaks were separated via preparative HPLC (Eurospher II C18 column 250 x 8 mm, 70% MeCN, 30% H_2O, 4 mL/min).

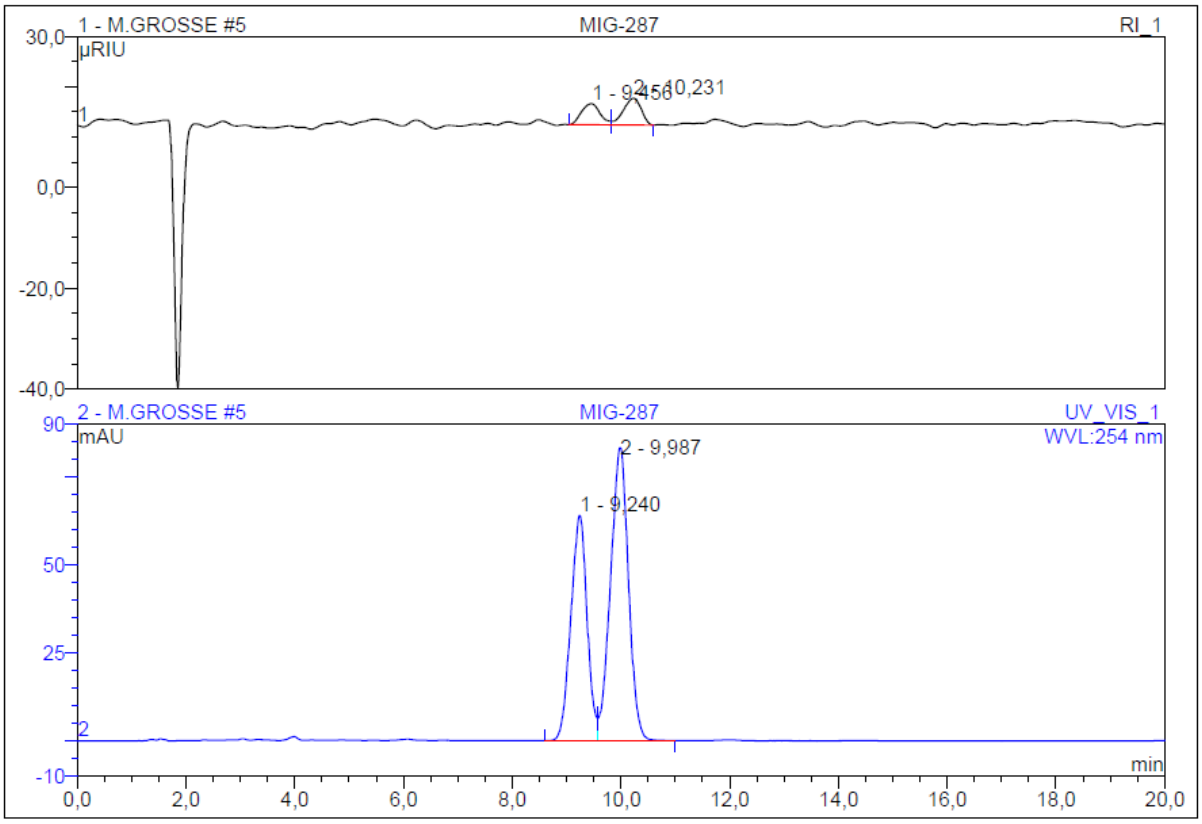

No.	Ret.Time min	Peak Name	Height µRIU	Area µRIU*min	Rel.Area %	Amount n.a.	Type
1	9,46	n.a.	4,184	1,671	45,53	n.a.	BM
2	10,23	n.a.	5,287	1,999	54,47	n.a.	MB
Total:			9,471	3,669	100,00	0,000	

No.	Ret.Time min	Peak Name	Height mAU	Area mAU*min	Rel.Area %	Amount n.a.	Type
1	9,24	n.a.	63,888	22,173	41,04	n.a.	BM
2	9,99	n.a.	83,120	31,851	58,96	n.a.	MB
Total:			147,007	54,024	100,00	0,000	

Figure S1. HPLC chromatogram (Eurospher II C18 column 250 x 4 mm, 70% MeCN, 30% H_2O, 1 mL/min) of the double Diels-Alder adducts (**6** and isomers).

The comparison of the NMR spectra of peaks 1 and 2 with the original product mixture are shown in Figure S2 and Figure S3. As can be seen from the ^{19}F NMR spectra (Figure S3), a mixture of products was obtained for both separated peaks. The compound mixture of peak 1 was obtained as a white, crystalline solid while the compound mixture of peak 2 was obtained as a white, waxy solid. We hypothesized that peak 1 corresponds to the isomers shown in Figure S4-A, as their nitrogen atoms are located on the same side of the molecule, resulting in a higher dipole moment and, consequently, increased polarity. Peak 2 was assigned to the isomers shown in Figure S4-B, as their nitrogen atoms are located on opposite sides of the molecule, resulting in a lower dipole moment and decreased polarity. This is supported by the ^{19}F NMR spectroscopic analysis: the spectrum that corresponds to peak 2 (Figure S3-C) shows two singlets (overlapping with two doublets), which can be assigned to the Diels-Alder adducts **S10** and **S11**, as both contain two equivalent fluorine atoms. In contrast, all other Diels-Alder adducts contain two non-equivalent fluorine atoms resulting in two doublets per isomer.

Single crystals suitable for X-ray diffraction measurements were obtained by vapor diffusion of *n*-pentane into a solution of the compound mixture of peak 1 in dichloromethane. The results of the X-ray diffraction measurements are shown in section 3.

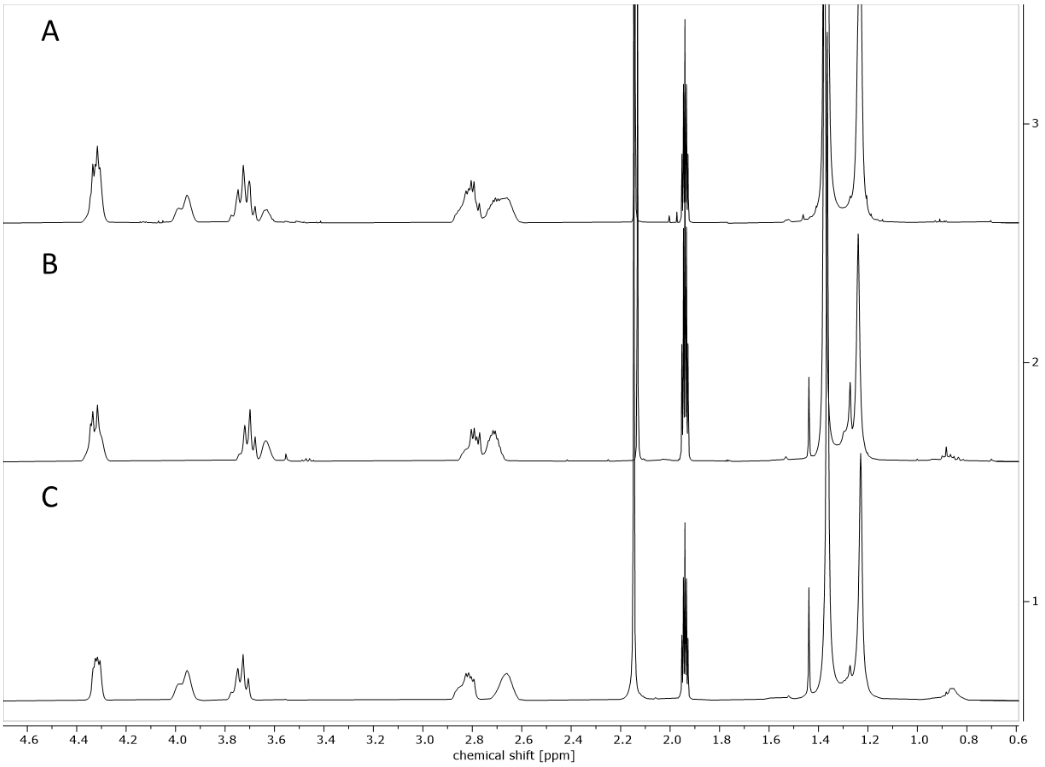

Figure S2. Excerpts of the ^1H NMR spectra (400 MHz, CD_3CN) of the double Diels-Alder adducts (**6** and isomers) before (A) and after (peak 1: B; peak 2: C) separation via preparative HPLC.

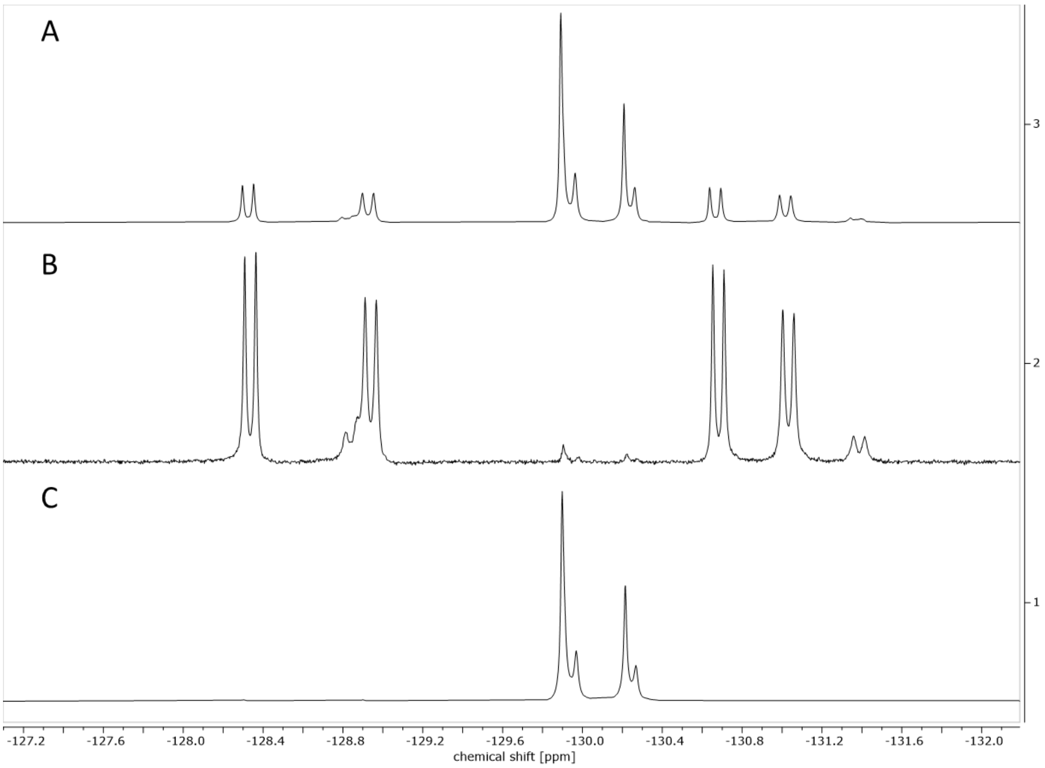

Figure S3. Excerpts of the $^{19}F\{^{1}H\}$ NMR spectra (377 MHz, CD_3CN) of the double Diels-Alder adducts (**6** and isomers) before (A) and after (peak 1: B; peak 2: C) separation via preparative HPLC.

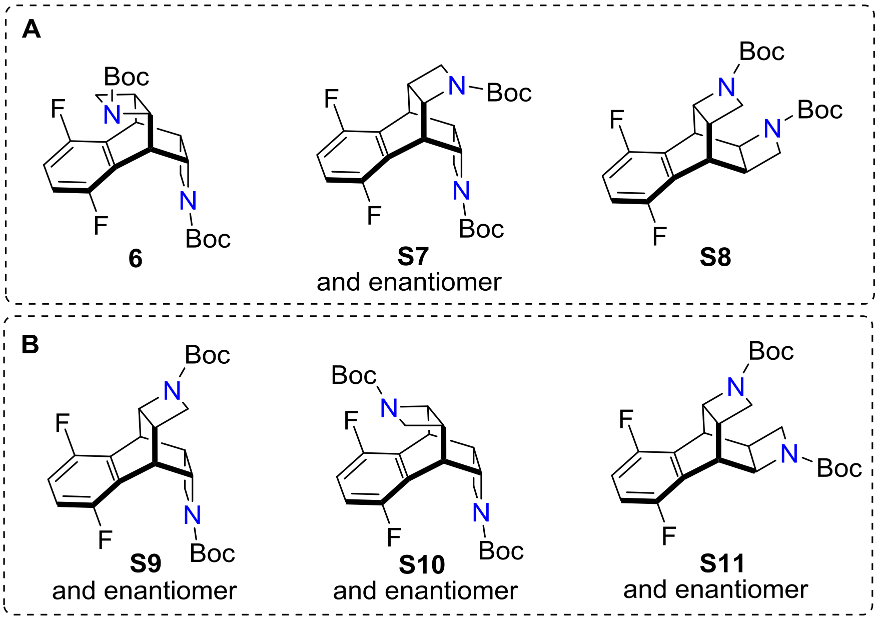

Figure S4. Structures of all possible isomers formed in the Diels-Alder reaction between *o*-QDM **4** and azetine **3**, grouped according to relative positions of the nitrogen atoms (A: nitrogen atoms on same side; B: nitrogen atoms on opposite sides).

1.5.2 Hemiaminal ether 7

To further elucidate the structure of the hemiaminal ether **7** formed from azocine **5a** in unstabilized $CDCl_3$ (see section 1.4.15), the obtained white solid was analyzed via chiral HPLC (Figure S5) and the two main peaks were separated via preparative chiral HPLC (CHIRAL ART Cellulose-SC column 250 x 10 mm, 85% *n*-hexane, 15% EtOAc, 4 mL/min). Two white, crystalline products were obtained.

Single crystals suitable for X-ray diffraction measurements were obtained by dissolving the product of peak 1 in EtOAc under heating and slowly evaporating the solvent. The results of the X-ray diffraction measurements are shown in section 3.

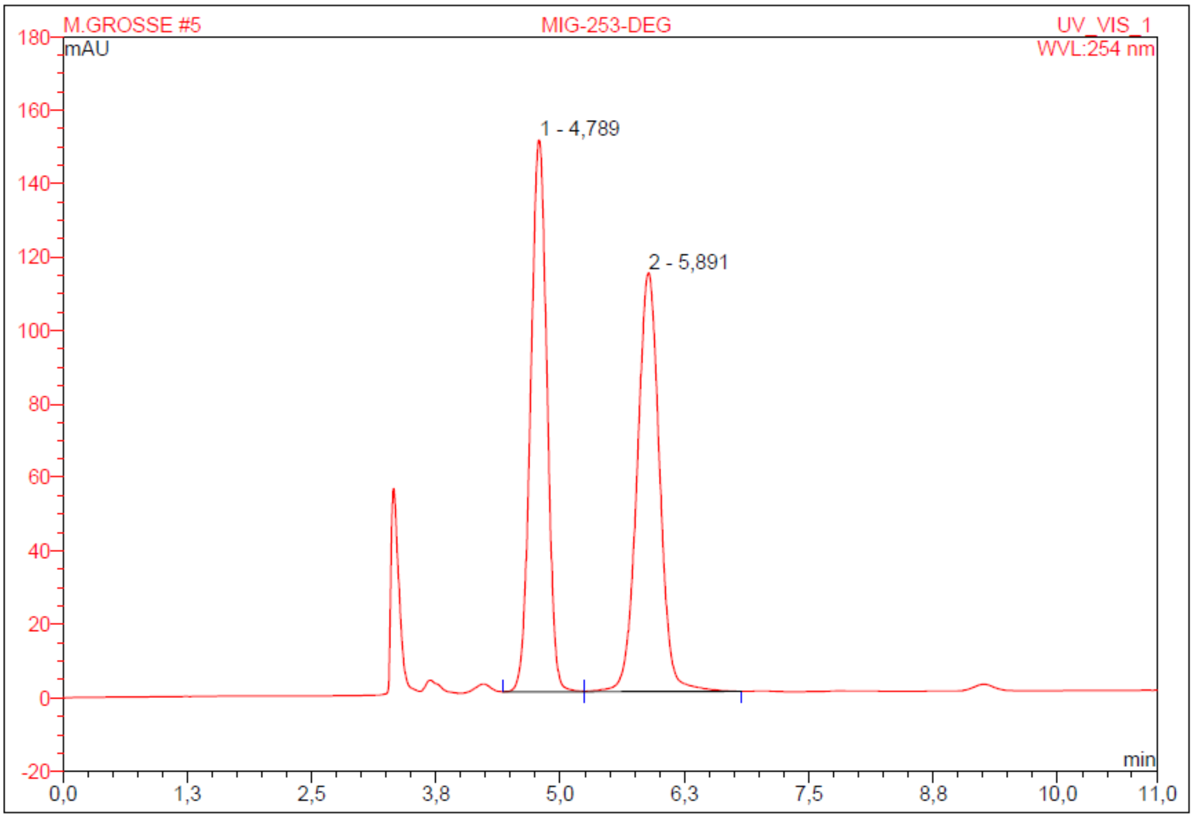

No.	Ret.Time min	Peak Name	Height mAU	Area mAU*min	Rel.Area %	Amount n.a.	Type
1	4,79	n.a.	150,181	28,673	48,97	n.a.	BM
2	5,89	n.a.	113,997	29,878	51,03	n.a.	MB
Total:			264,179	58,551	100,00	0,000	

Figure S5. Chiral HPLC chromatogram (CHIRALPAK IC column 250 x 4.6 mm, 85% *n*-hexane, 15% EtOAc, 1 mL/min) of the hemiaminal ether **7**.

2 NMR spectra

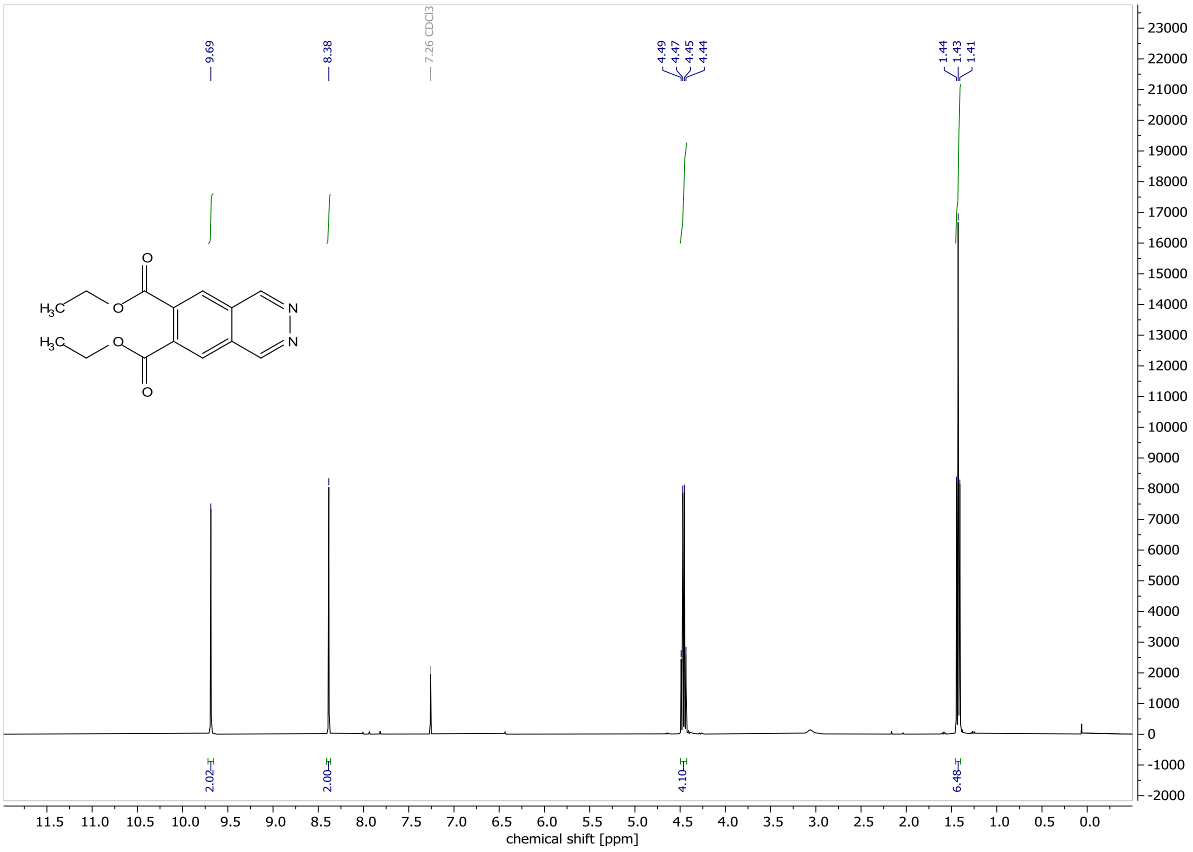

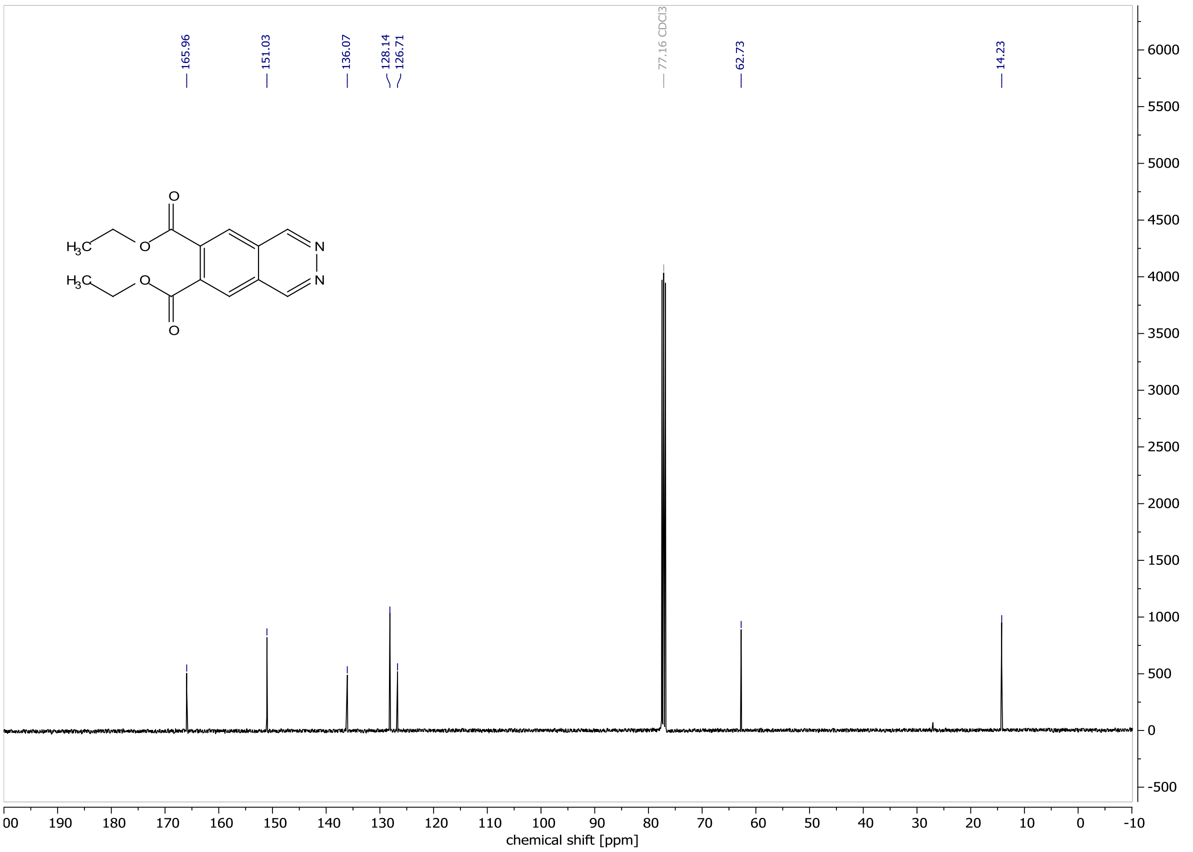

Figure S6. ^{1}H (400 MHz) (top) and ^{13}C{^{1}H} (101 MHz) (bottom) NMR spectra ($CDCl_3$) of diethyl phthalazine-6,7-dicarboxylate (**1j**).

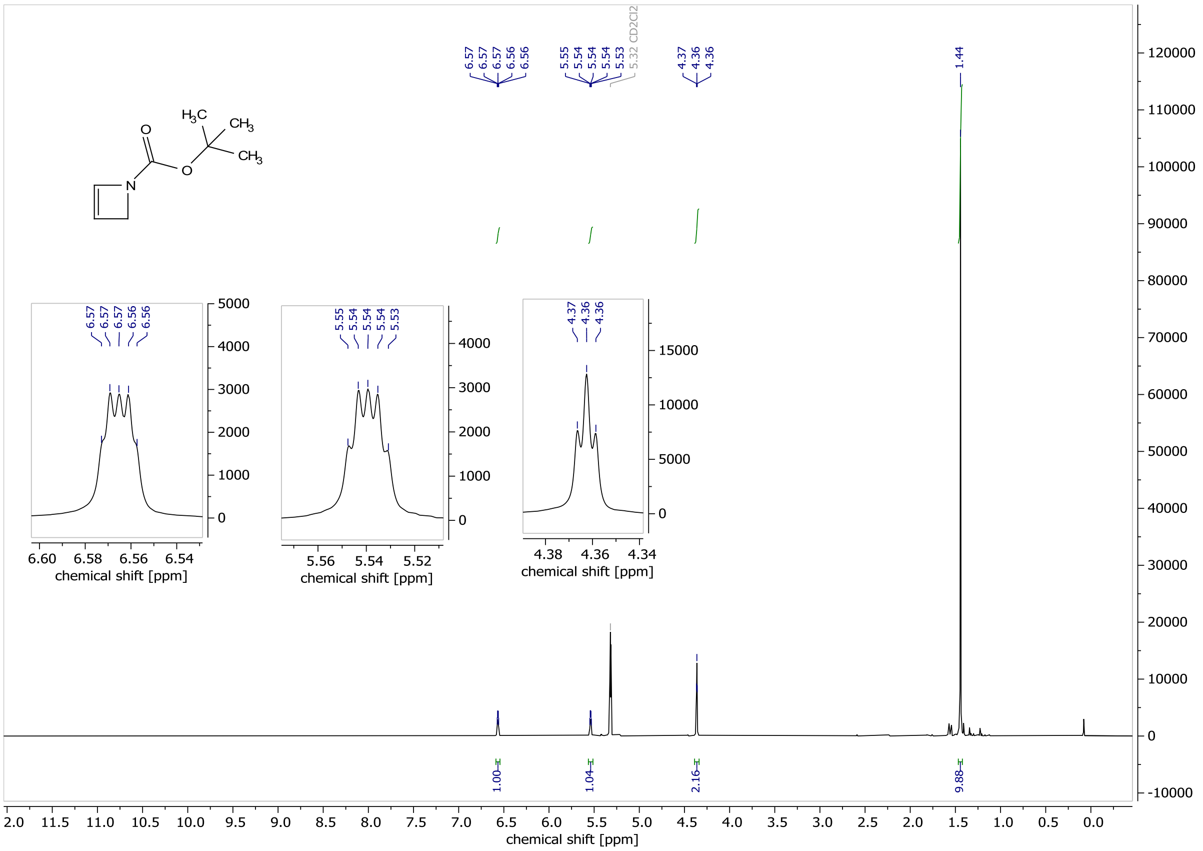

Figure S7. ^{1}H (200 MHz) NMR spectrum (CD_2Cl_2) of *tert*-butyl azete-1(2*H*)-carboxylate (**3**).

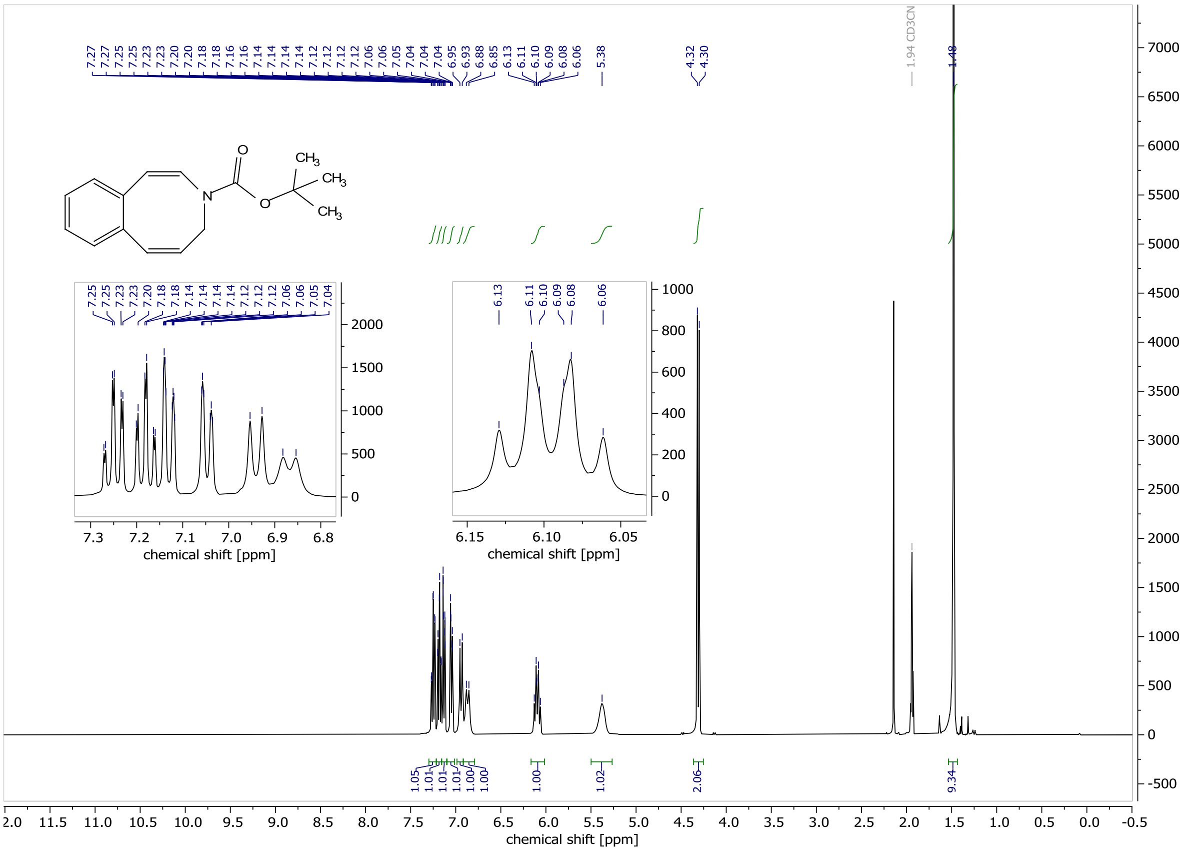

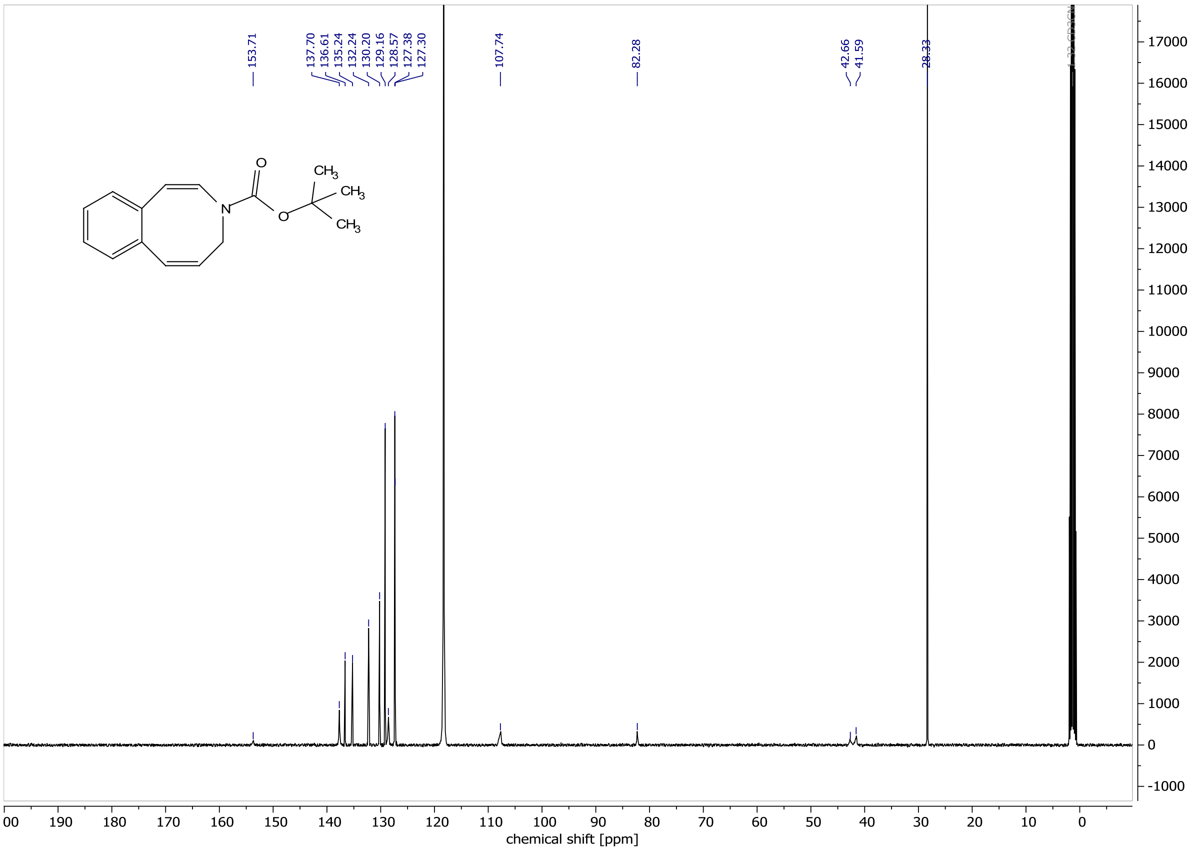

Figure S8. ^{1}H (400 MHz) (top) and $^{13}C\{^{1}H\}$ (101 MHz) (bottom) NMR spectra (CD_3CN) of *tert*-butyl (1*Z*,5*Z*)-benzo[*d*]azocine-3(4*H*)-carboxylate (**5a**).

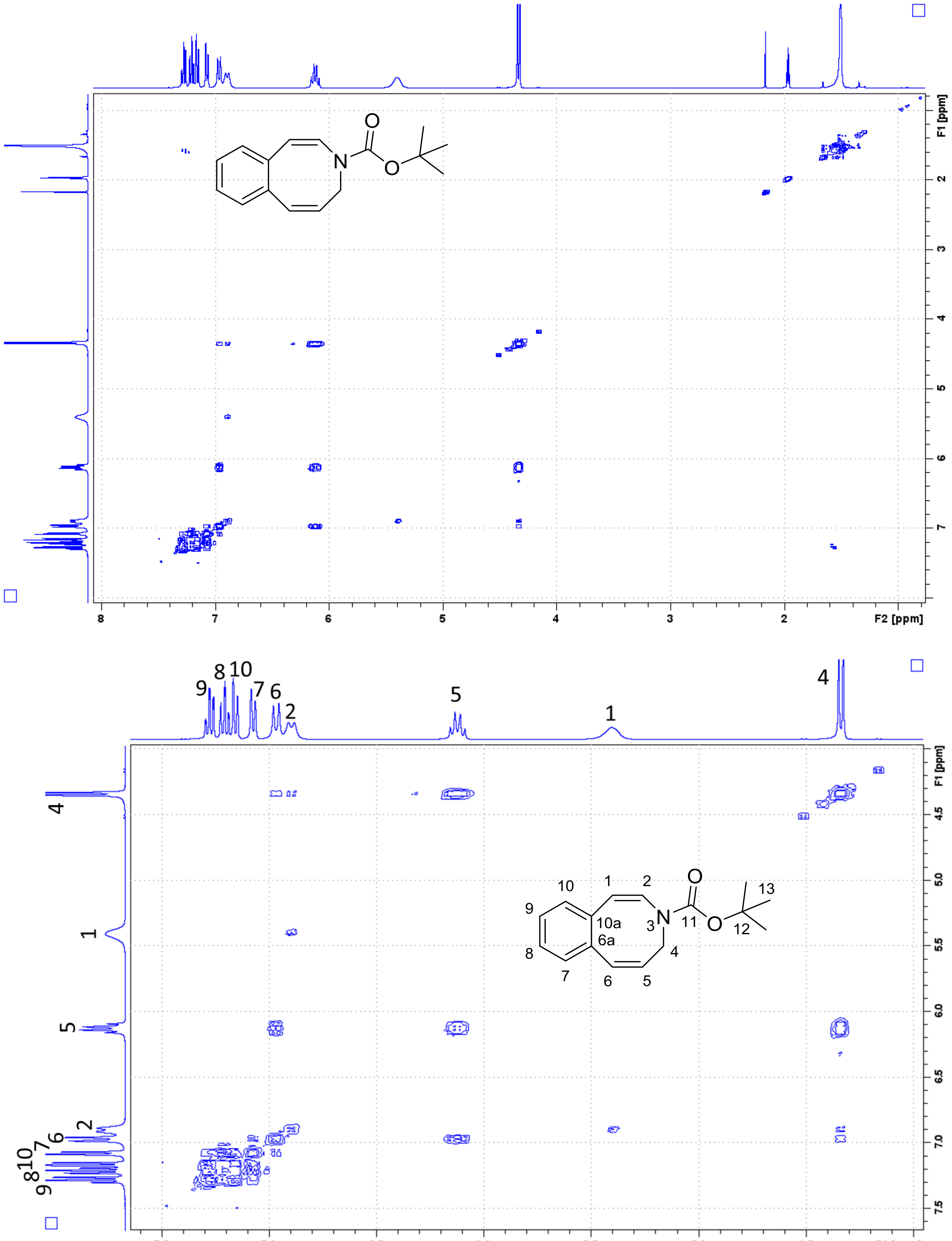

Figure S9. ^{1}H-^{1}H COSY (400 MHz, CD_3CN) NMR spectrum (top: full, bottom: excerpt with peak assignment) of *tert*-butyl (1*Z*,5*Z*)-benzo[*d*]azocine-3(4*H*)-carboxylate (**5a**).

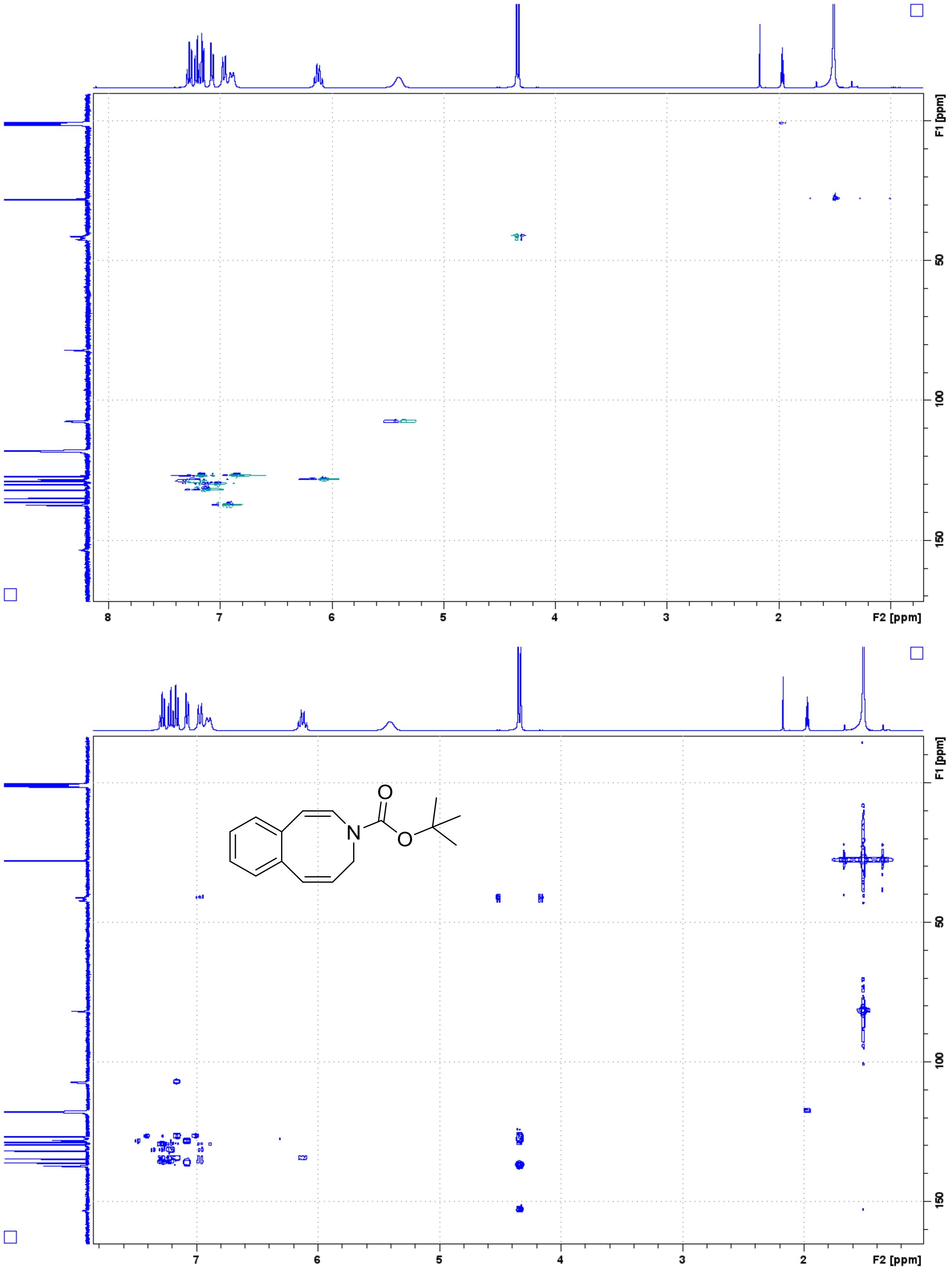

Figure S10. ^{1}H-^{13}C HSQC (top) and ^{1}H-^{13}C HMBC (bottom) NMR spectra (CD_3CN) of *tert*-butyl (1*Z*,5*Z*)-benzo[*d*]azocine-3(4*H*)-carboxylate (**5a**).

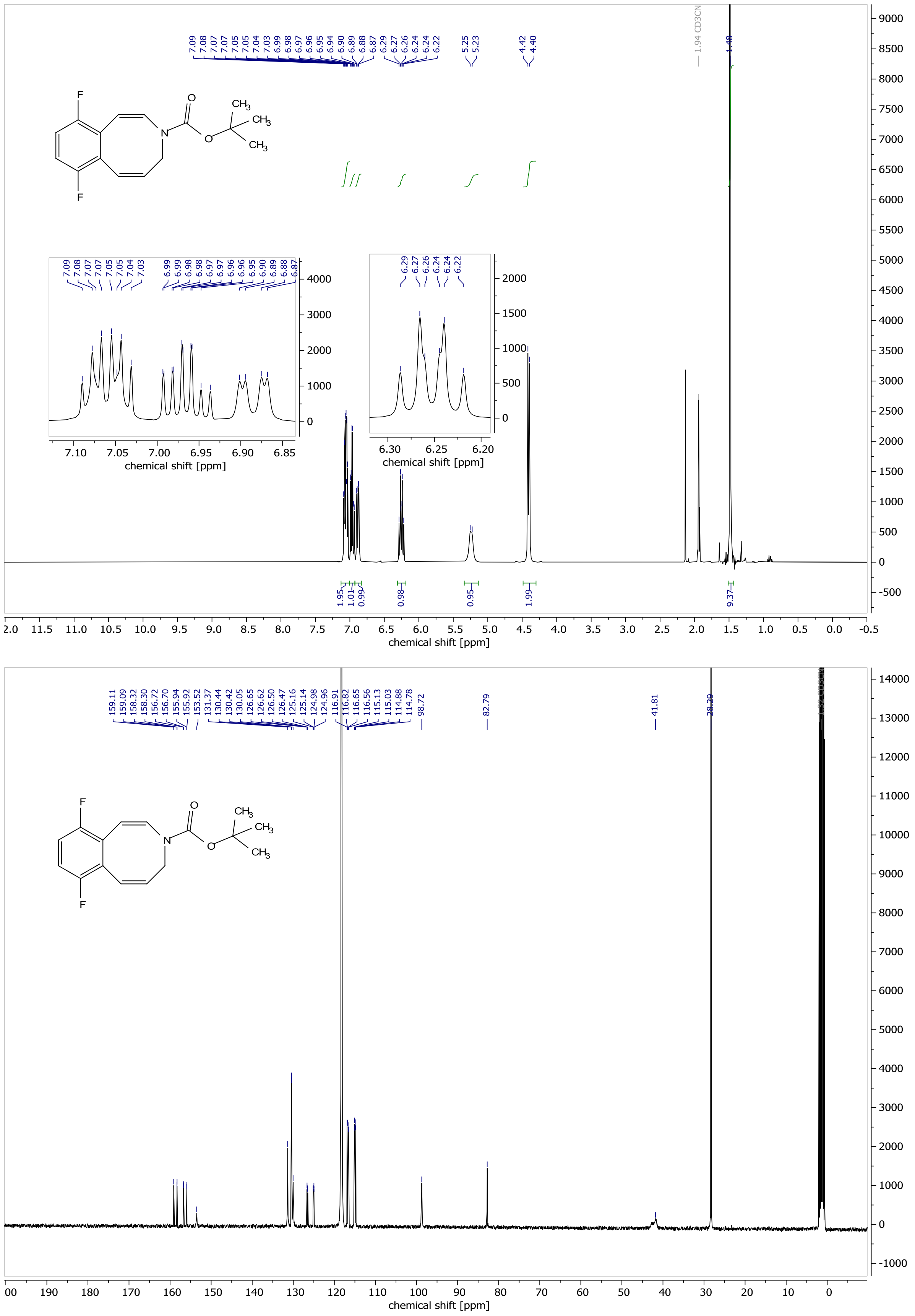

Figure S11. ^{1}H (400 MHz) (top) and $^{13}C\{^{1}H\}$ (101 MHz) (bottom) NMR spectra (CD_3CN) of *tert*-butyl (1*Z*,5*Z*)-7,10-difluorobenzo[*d*]azocine-3(4*H*)-carboxylate (**5b**).

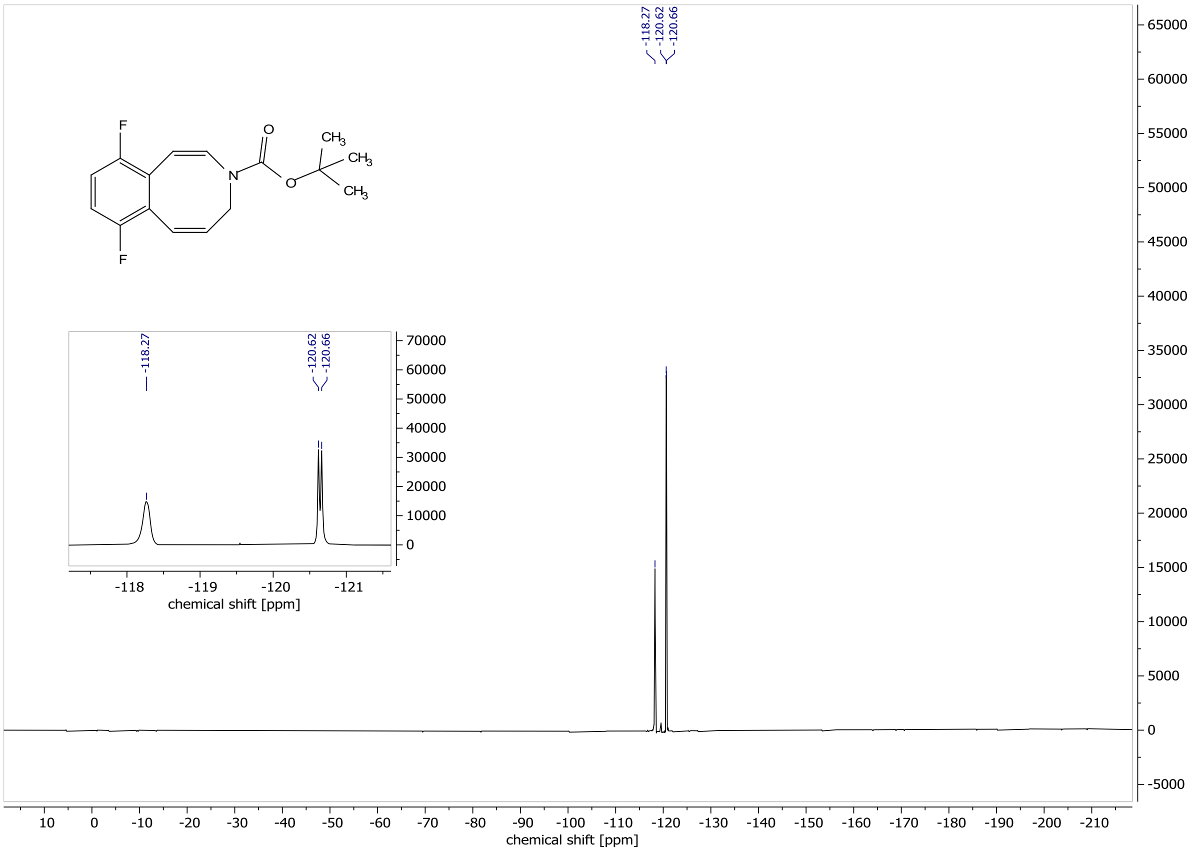

Figure S12. $^{19}F\{^1H\}$ (377 MHz) NMR spectrum (CD_3CN) of *tert*-butyl (1*Z*,5*Z*)-7,10-difluorobenzo[*d*]azocine-3(4*H*)-carboxylate (**5b**).

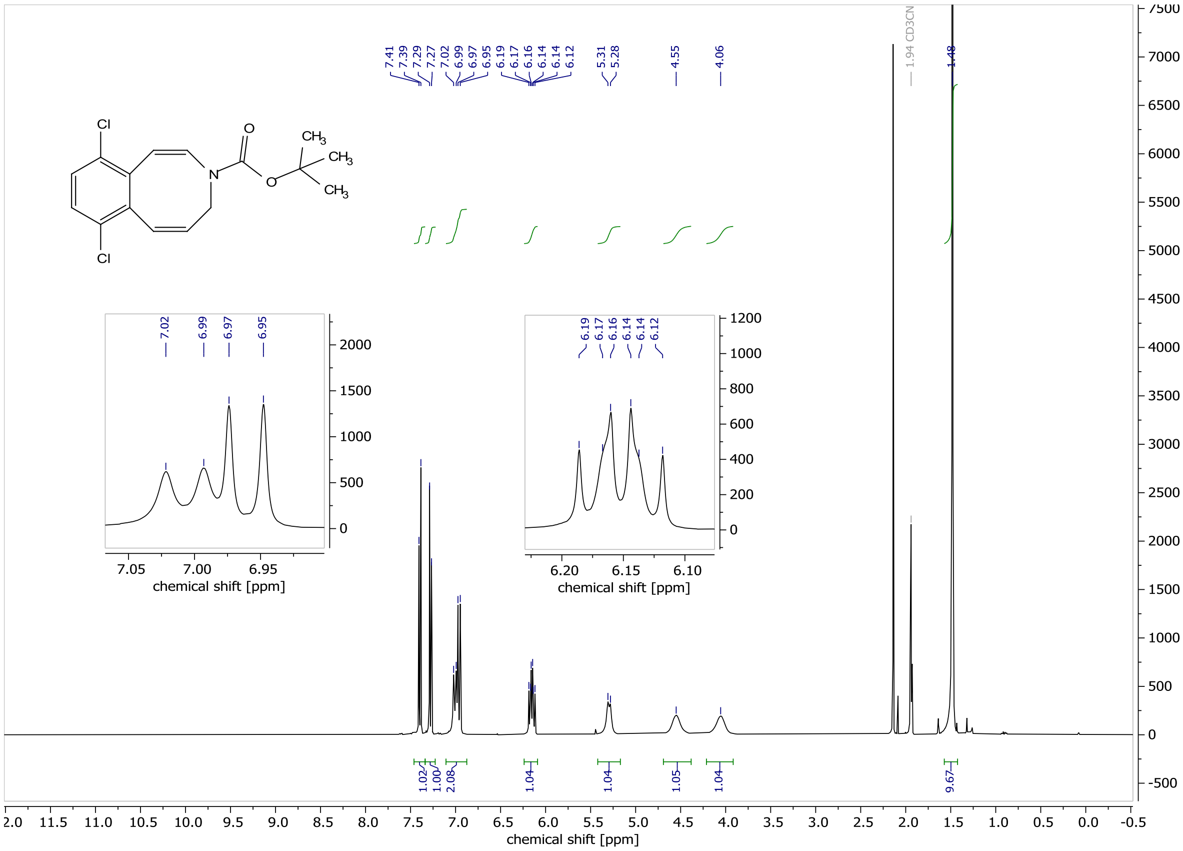

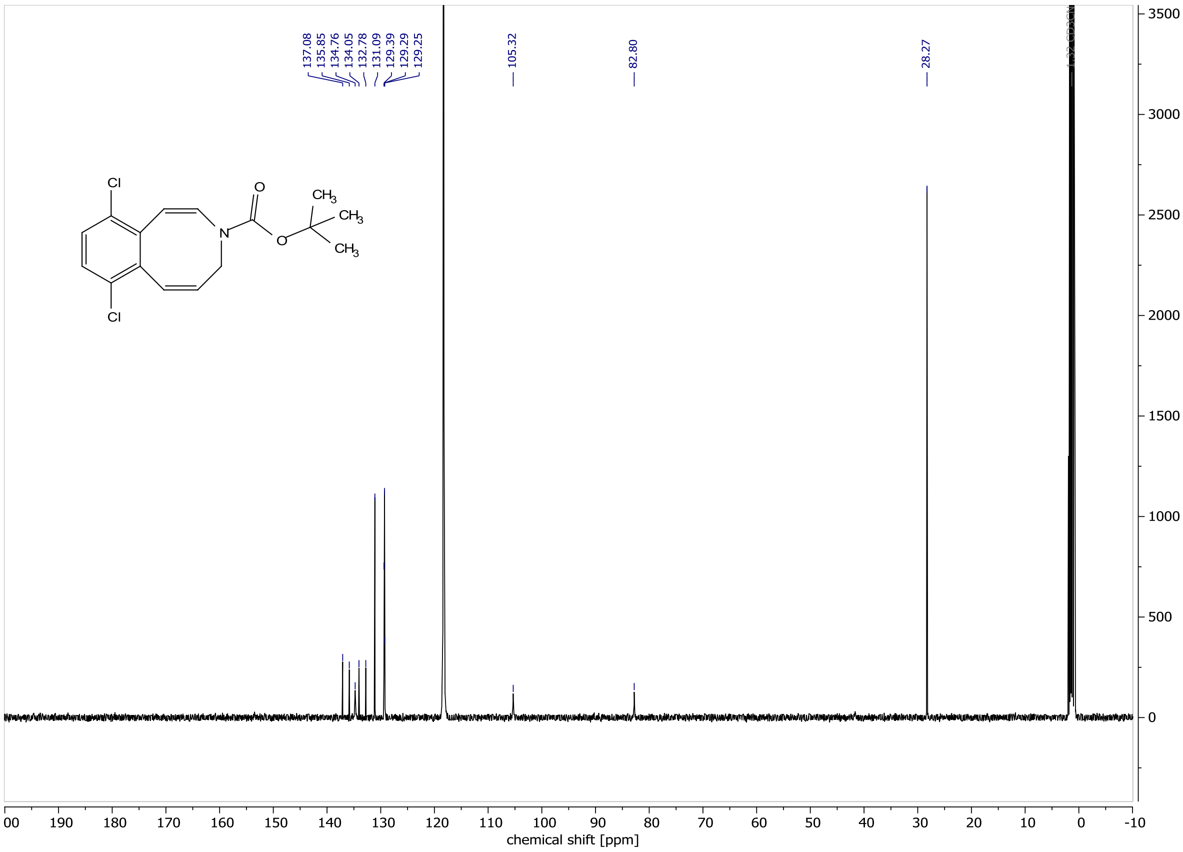

Figure S13. ^{1}H (400 MHz) (top) and ^{13}C{^{1}H} (101 MHz) (bottom) NMR spectra (CD_3CN) of *tert*-butyl (1*Z*,5*Z*)-7,10-dichlorobenzo[*d*]azocine-3(4*H*)-carboxylate (**5c**).

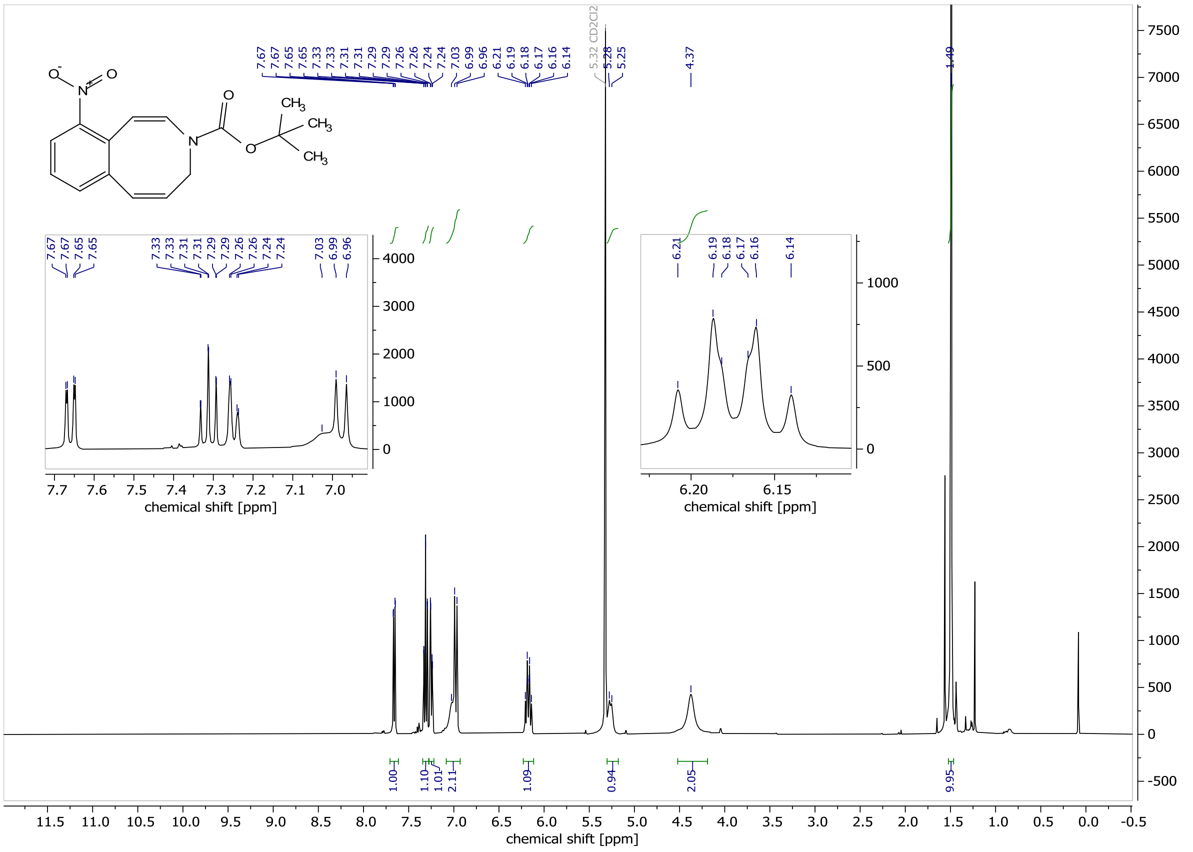

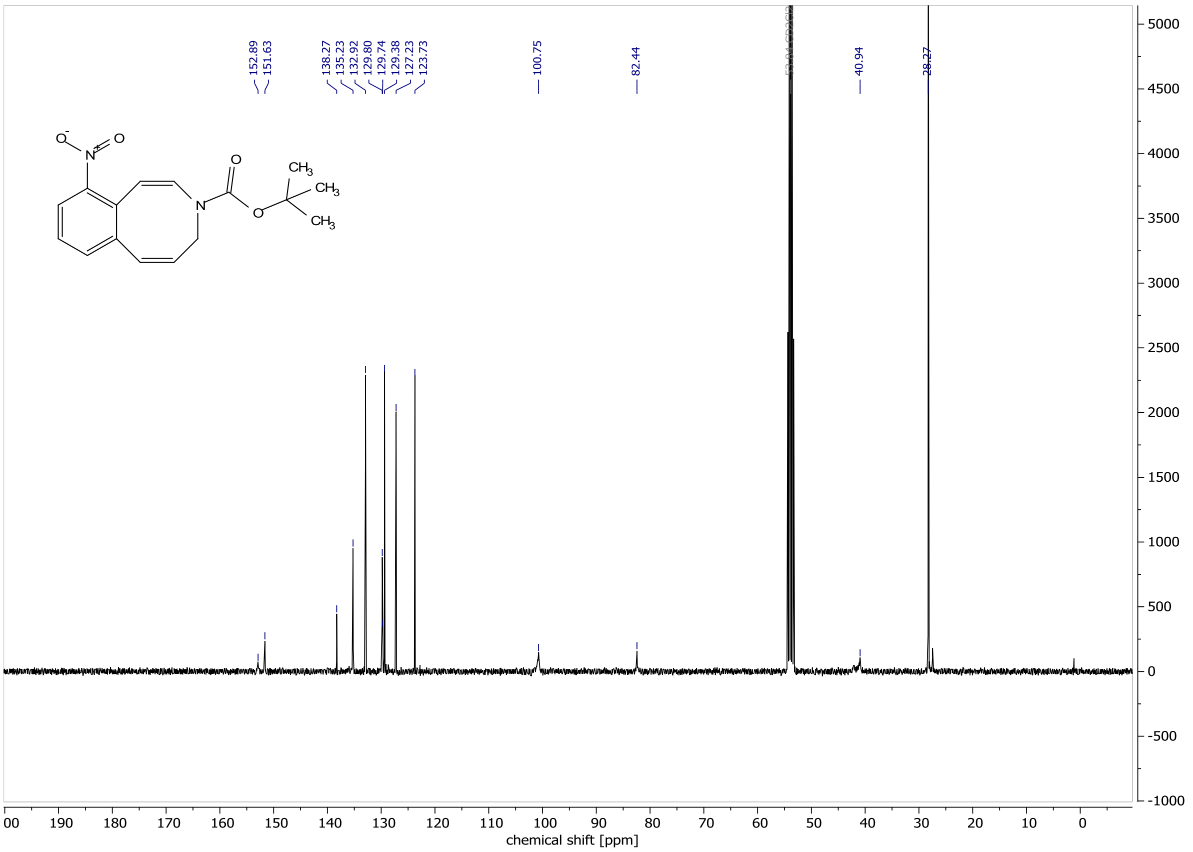

Figure S14. ^{1}H (400 MHz) (top) and $^{13}C\{^{1}H\}$ (101 MHz) (bottom) NMR spectra (CD_2Cl_2) of *tert*-butyl (1*Z*,5*Z*)-10-nitrobenzo[*d*]azocine-3(4*H*)-carboxylate (**5da**).

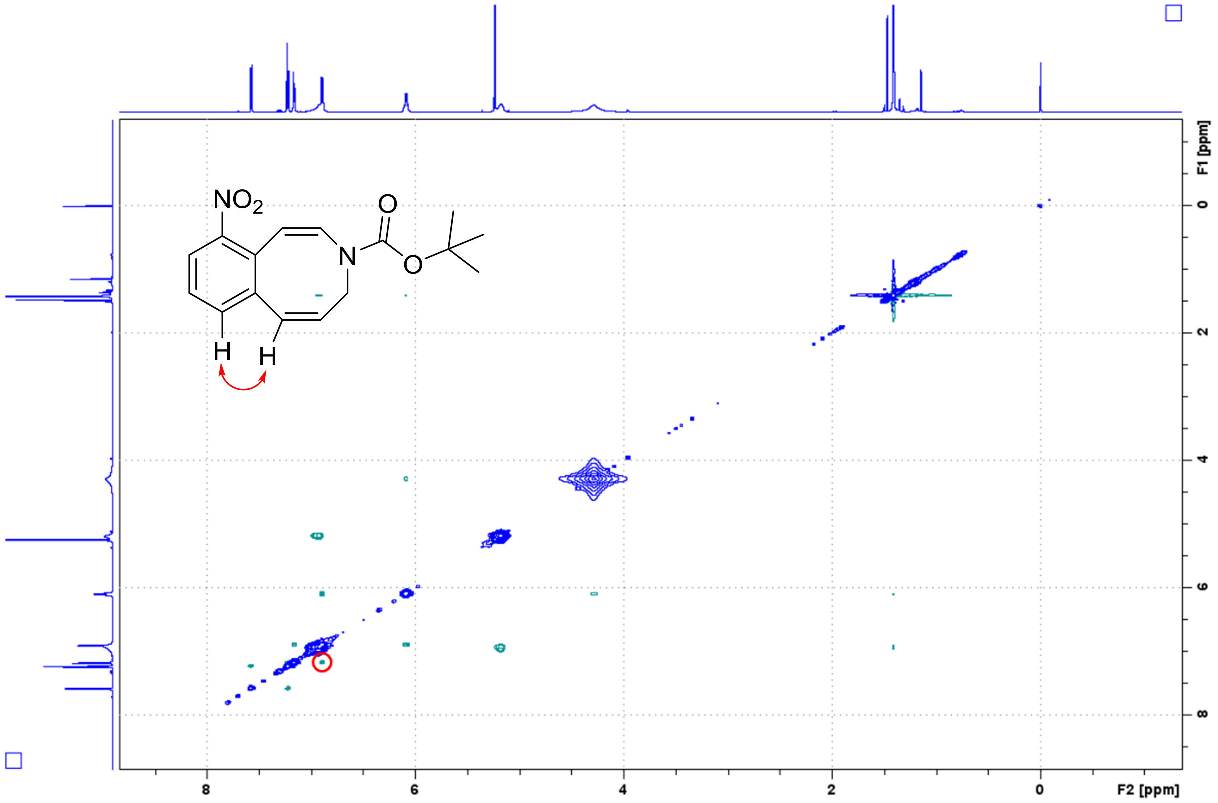

Figure S15. NOESY NMR spectrum (CD_2Cl_2) of *tert*-butyl (1*Z*,5*Z*)-7-nitrobenzo[*d*]azocine-3(4*H*)-carboxylate (**5da**).

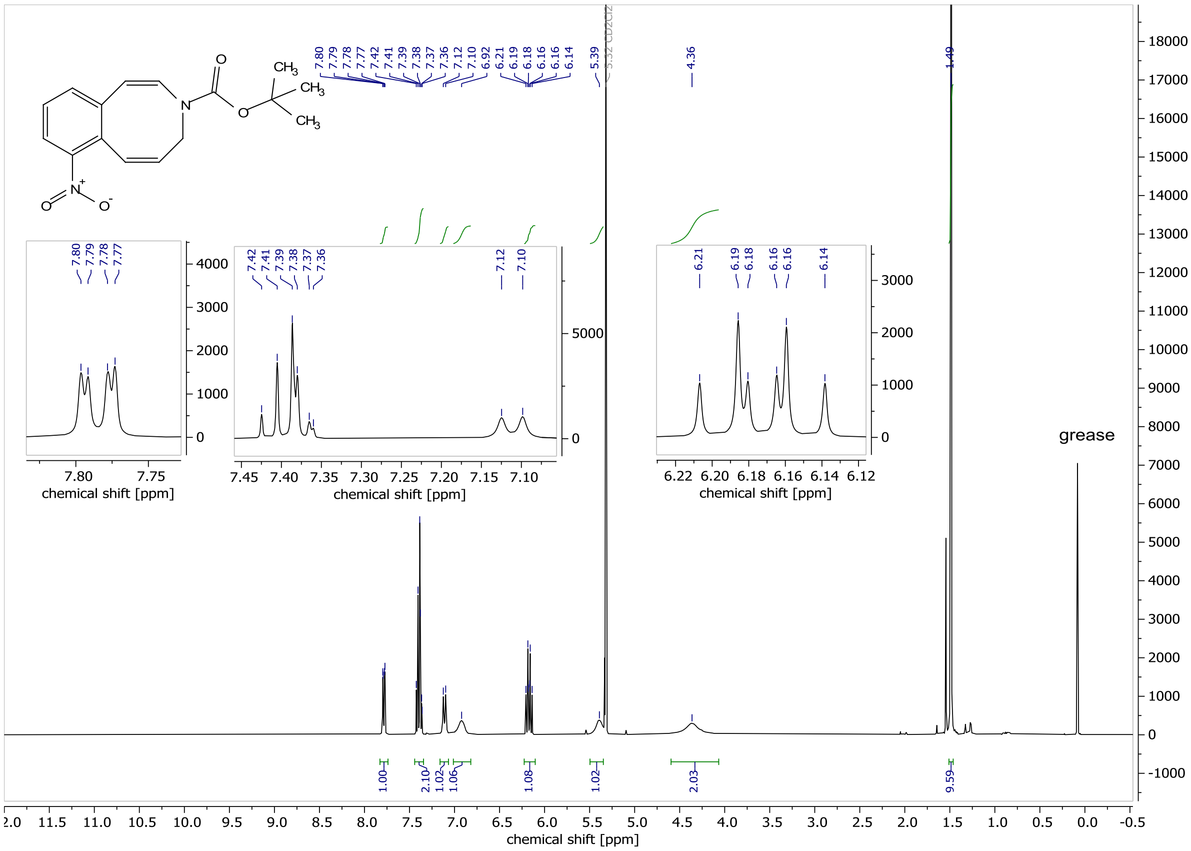

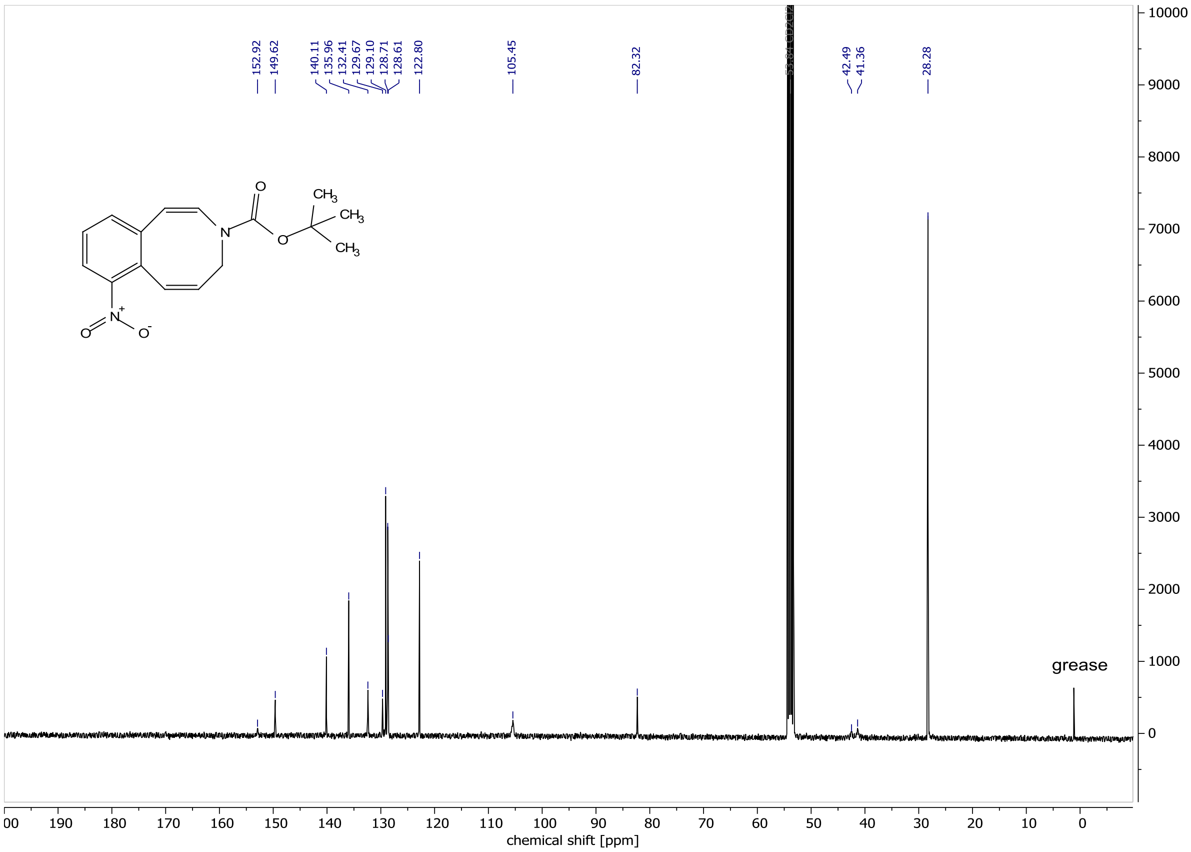

Figure S16. ^{1}H (400 MHz) (top) and $^{13}C\{^{1}H\}$ (101 MHz) (bottom) NMR spectra (CD_2Cl_2) of *tert*-butyl (1*Z*,5*Z*)-7-nitrobenzo[*d*]azocine-3(4*H*)-carboxylate (**5db**).

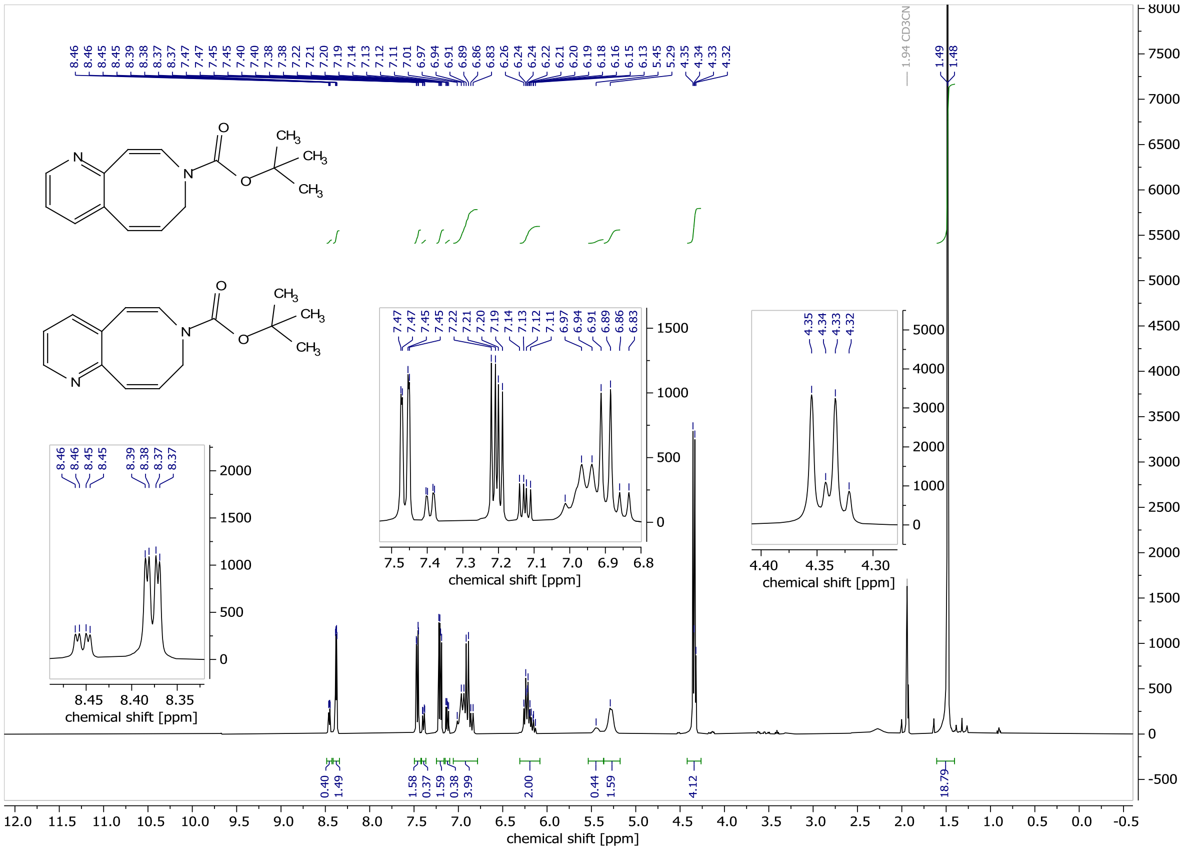

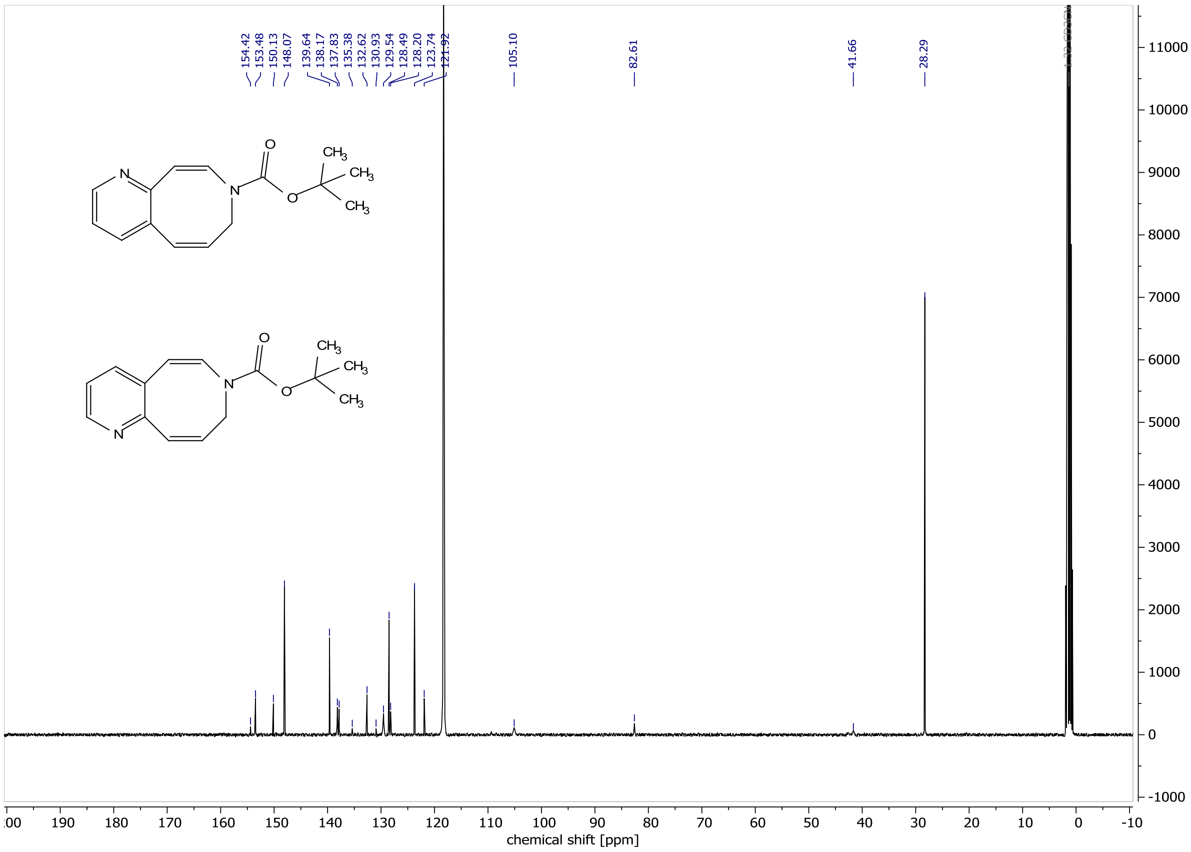

Figure S17. ^{1}H (400 MHz) (top) and $^{13}C\{^{1}H\}$ (101 MHz) (bottom) NMR spectra (CD_3CN) of *tert*-butyl (5*Z*,9*Z*)-pyrido[2,3-*d*]azocine-8(7*H*)-carboxylate/*tert*-butyl (5*Z*,9*Z*)-pyrido[3,2-*d*]azocine-7(8*H*)-carboxylate (**5e**).

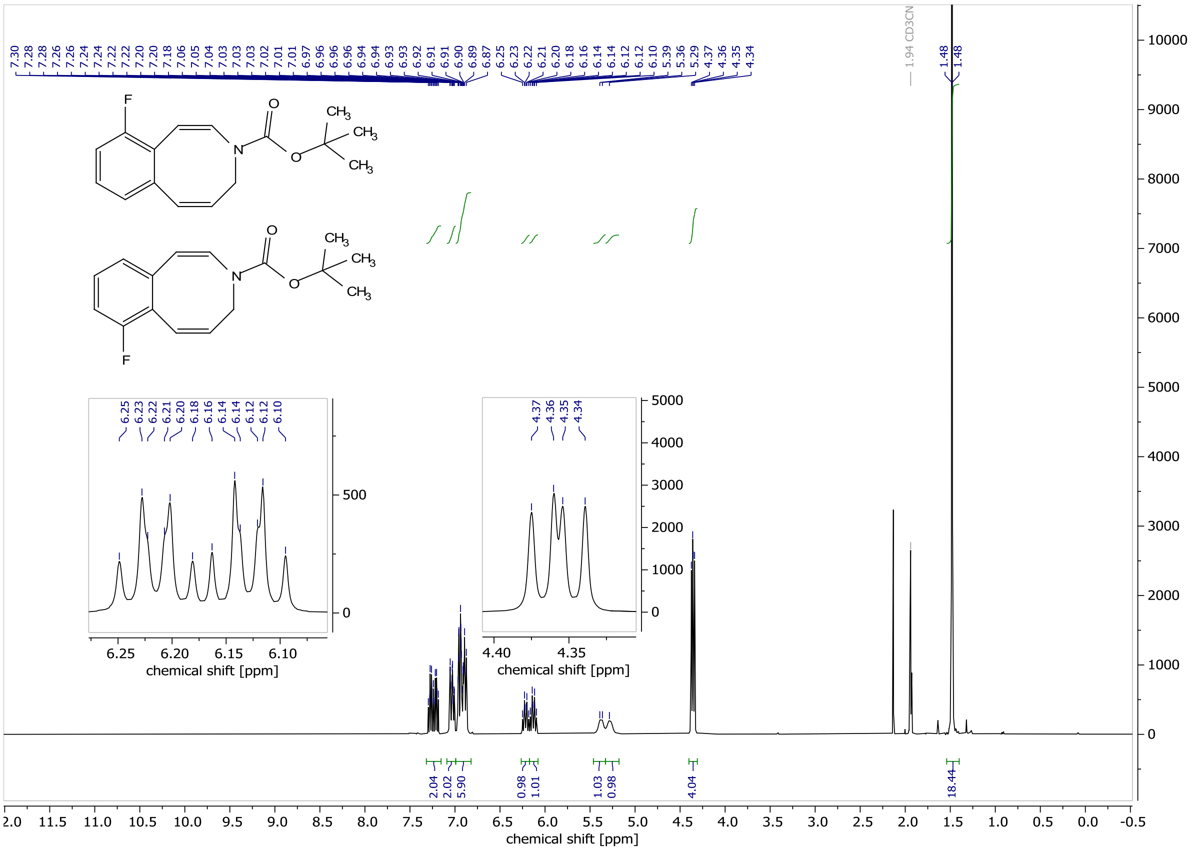

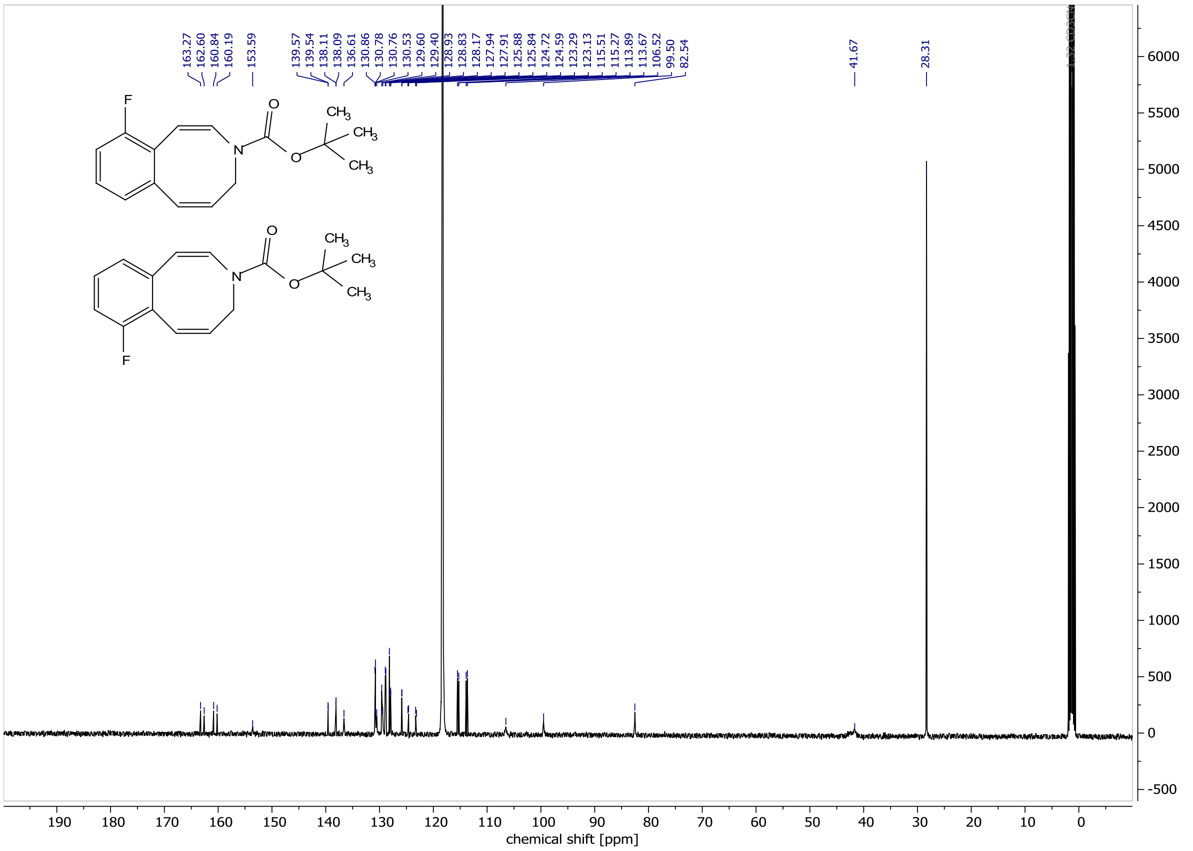

Figure S18. ^{1}H (400 MHz) (top) and $^{13}C\{^{1}H\}$ (101 MHz) (bottom) NMR spectra (CD_3CN) of *tert*-butyl (1*Z*,5*Z*)-7/10-fluorobenzo[*d*]azocine-3(4*H*)-carboxylate (**5f**).

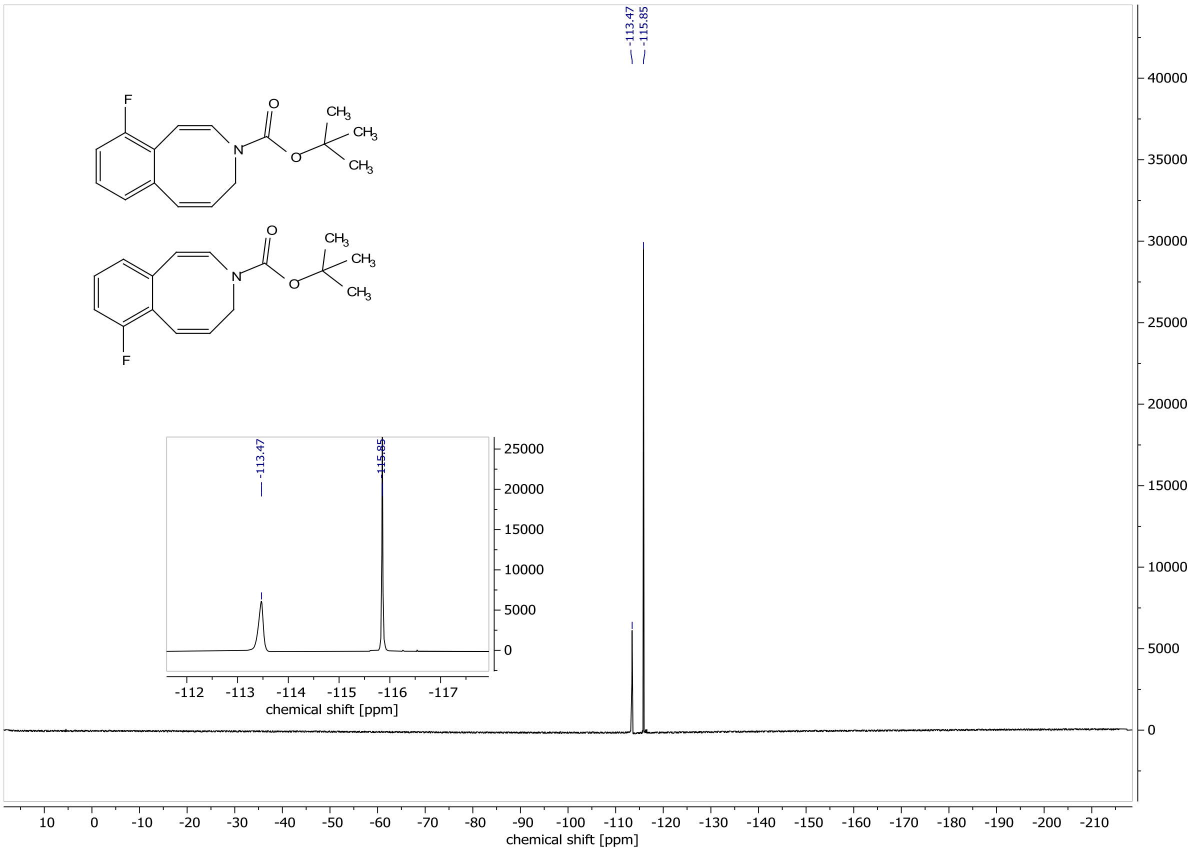

Figure S19. $^{19}F\{^{1}H\}$ (377 MHz) NMR spectrum (CD_3CN) of *tert*-butyl (1*Z*,5*Z*)-7/10-fluorobenzo[*d*]azocine-3(4*H*)-carboxylate (**5f**).

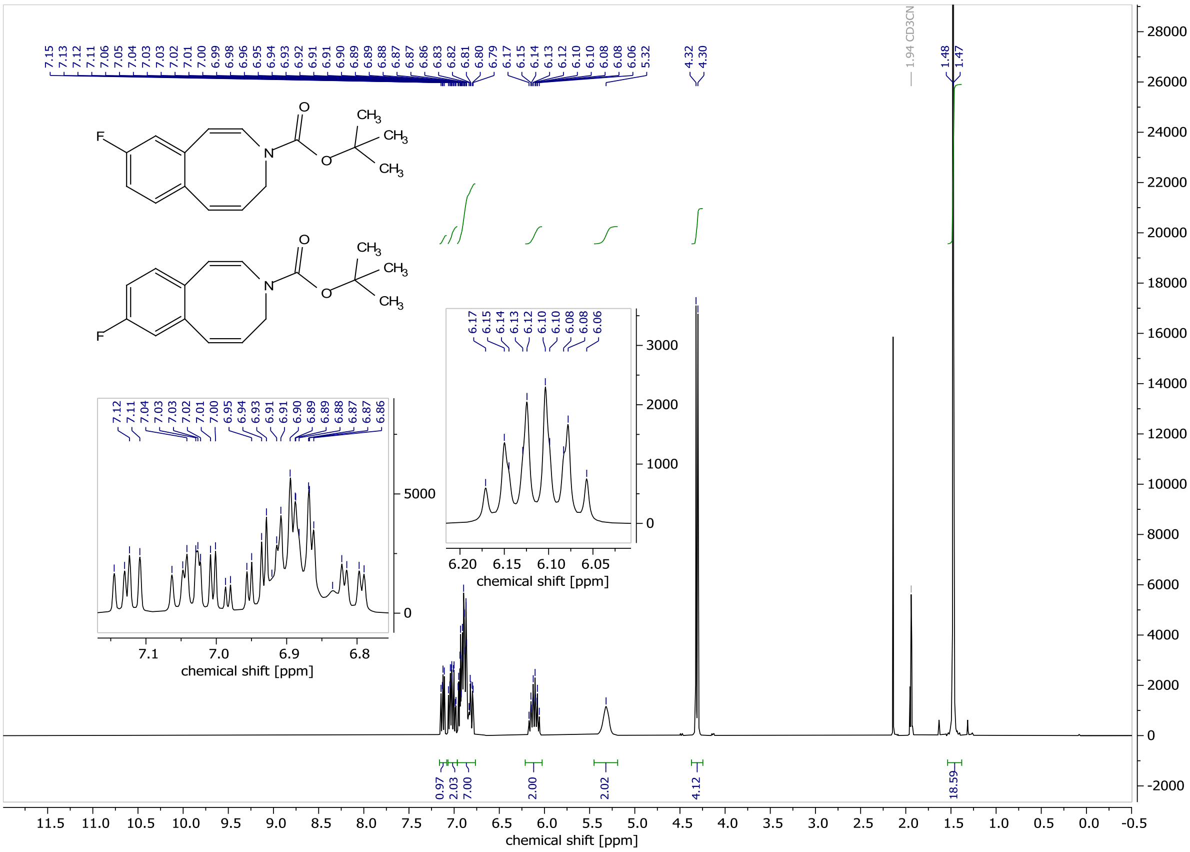

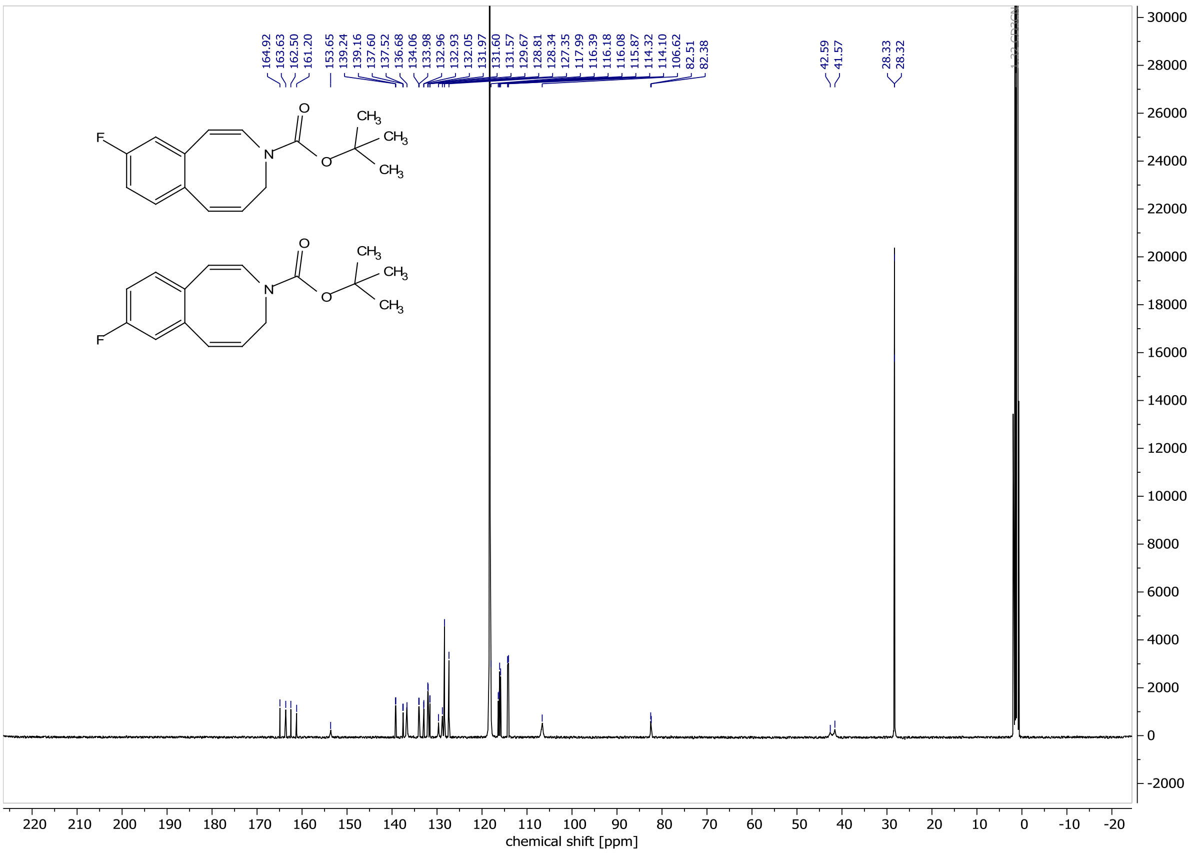

Figure S20. ^{1}H (400 MHz) (top) and ^{13}C{^{1}H} (101 MHz) (bottom) NMR spectra (CD_3CN) of *tert*-butyl (1*Z*,5*Z*)-8/9-fluorobenzo[*d*]azocine-3(4*H*)-carboxylate (**5g**).

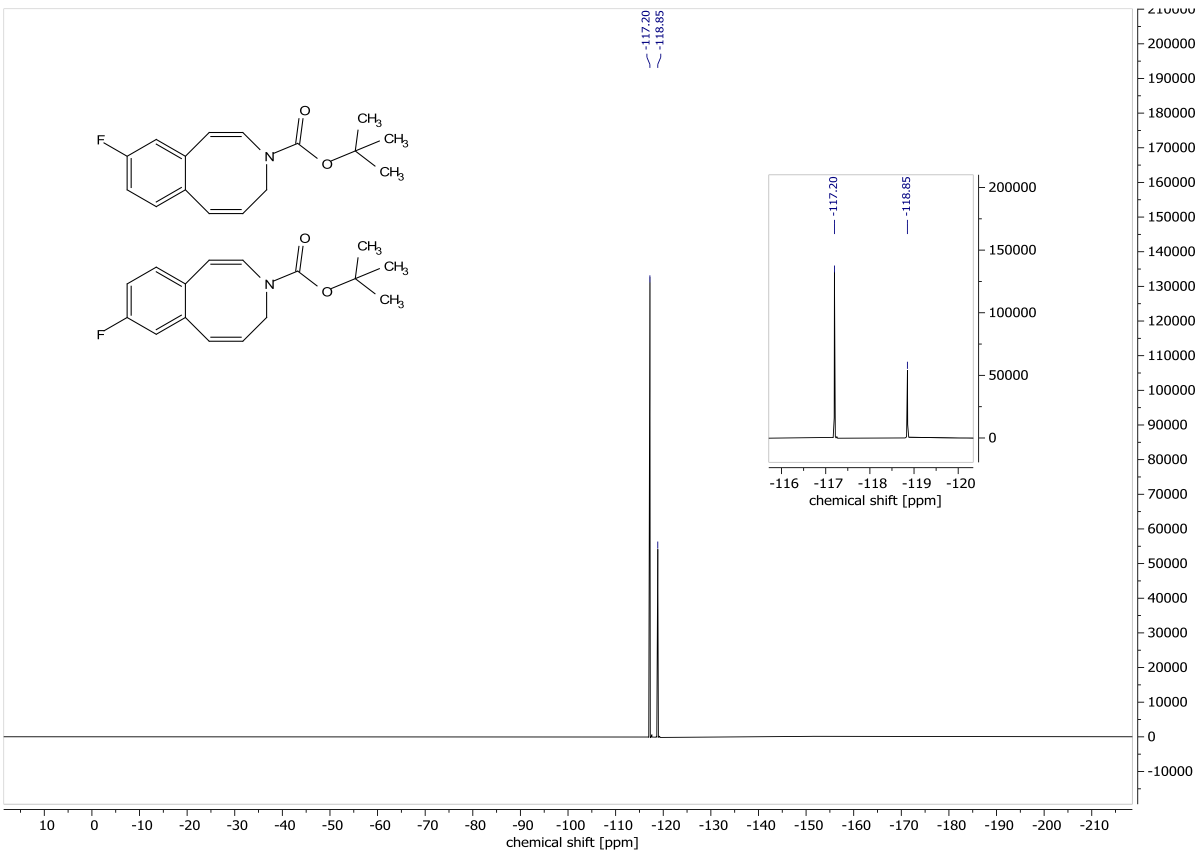

Figure S21. $^{19}F\{^{1}H\}$ (377 MHz) NMR spectrum (CD_3CN) of *tert*-butyl (1*Z*,5*Z*)-8/9-fluorobenzo[*d*]azocine-3(4*H*)-carboxylate (**5g**).

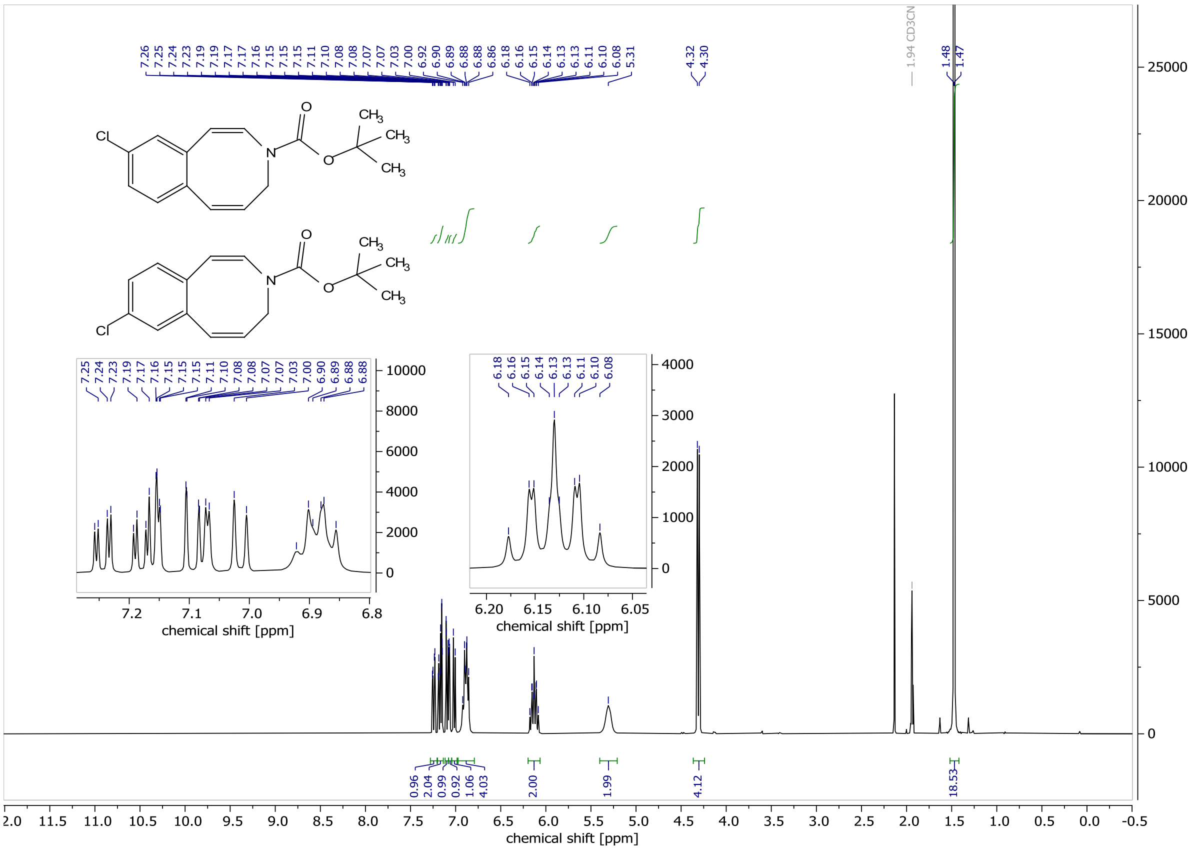

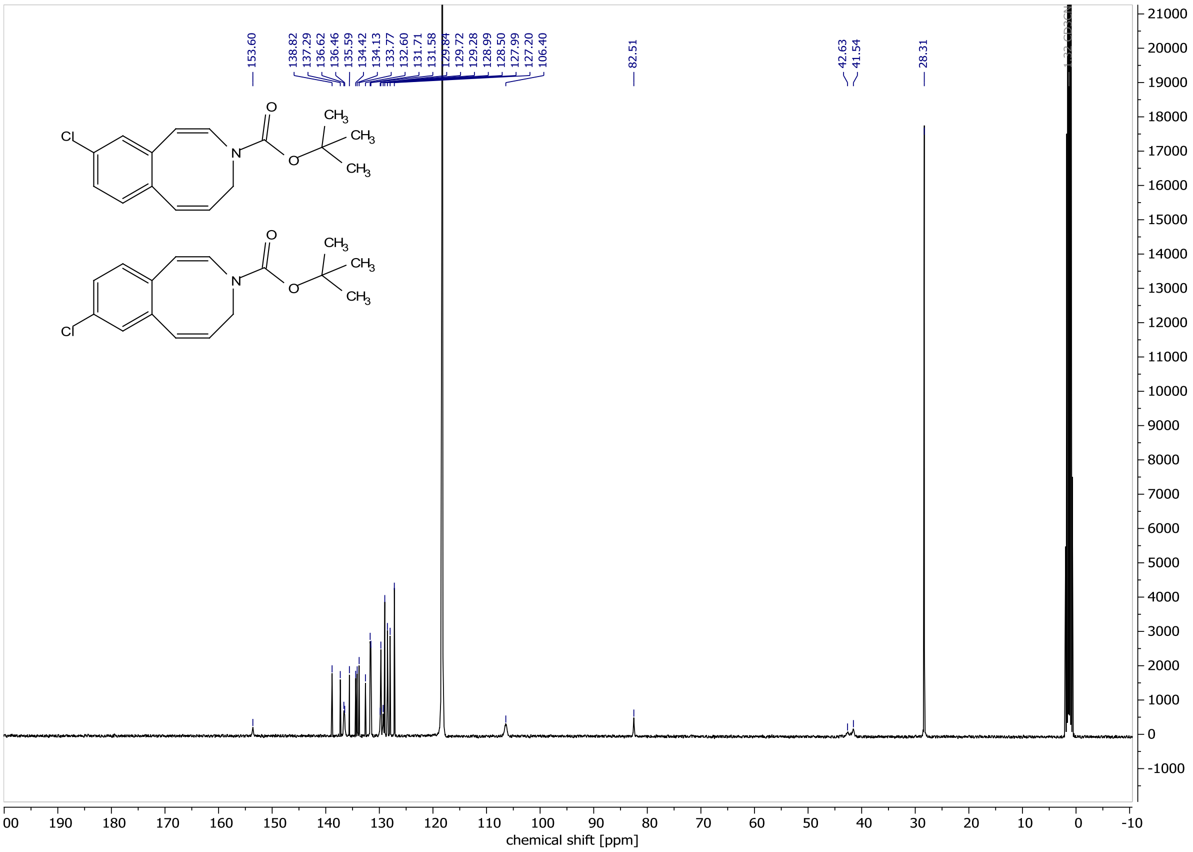

Figure S22. ^{1}H (400 MHz) (top) and $^{13}C\{^{1}H\}$ (101 MHz) (bottom) NMR spectra (CD_3CN) of *tert*-butyl (1*Z*,5*Z*)-8/9-chlorobenzo[*d*]azocine-3(4*H*)-carboxylate (**5h**).

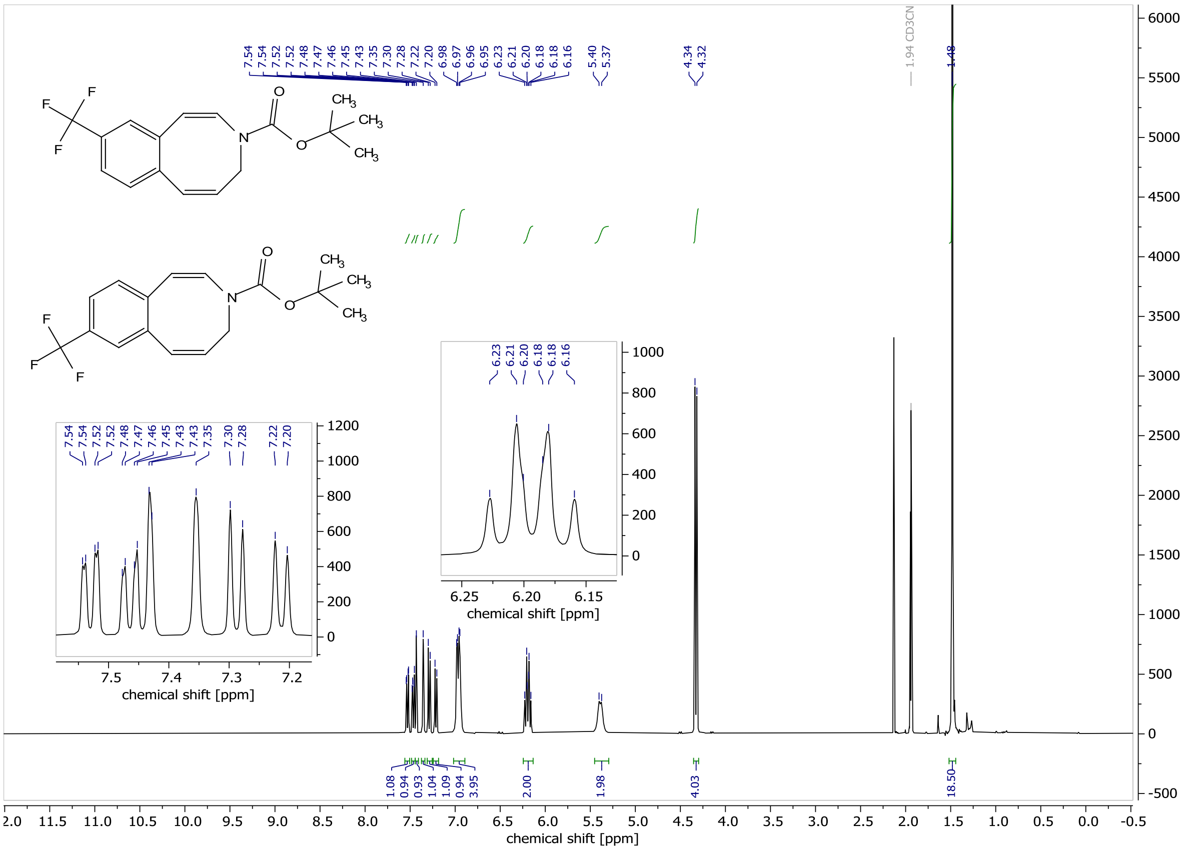

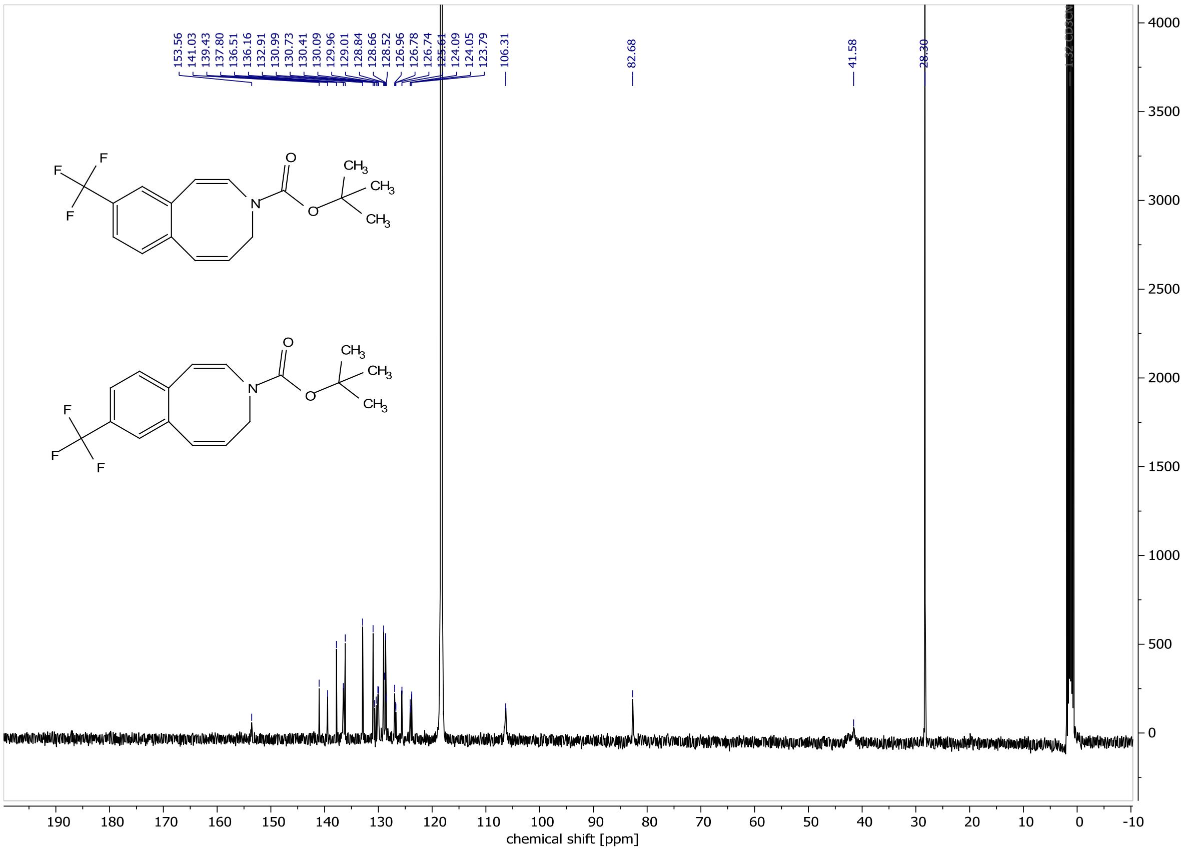

Figure S23. ^{1}H (400 MHz) (top) and $^{13}C\{^{1}H\}$ (101 MHz) (bottom) NMR spectra (CD_3CN) of *tert*-butyl (1*Z*,5*Z*)-8/9-(trifluoromethyl)benzo[*d*]azocine-3(4*H*)-carboxylate (**5i**).

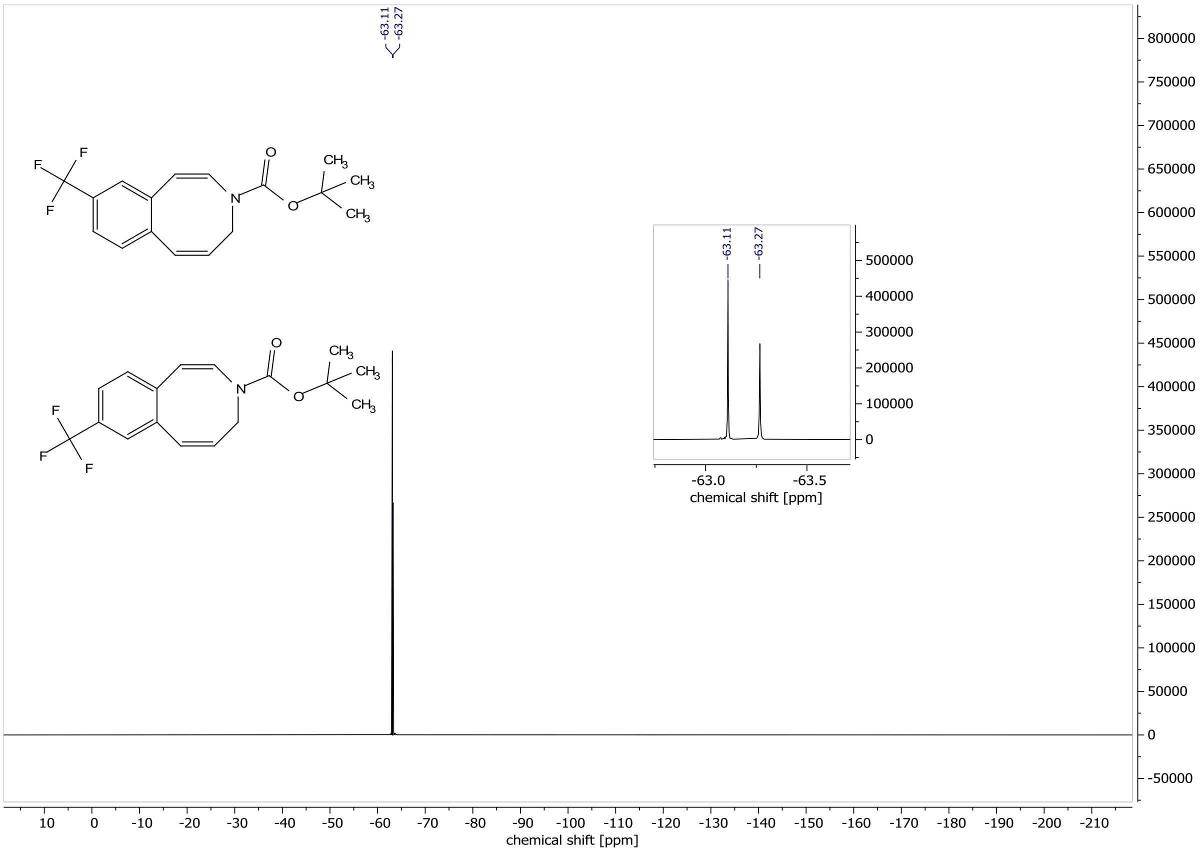

Figure S24. ^{19}F{^{1}H} (377 MHz) NMR spectrum (CD_3CN) of *tert*-butyl (1*Z*,5*Z*)-8/9-(trifluoromethyl)benzo[*d*]azocine-3(4*H*)-carboxylate (**5i**).

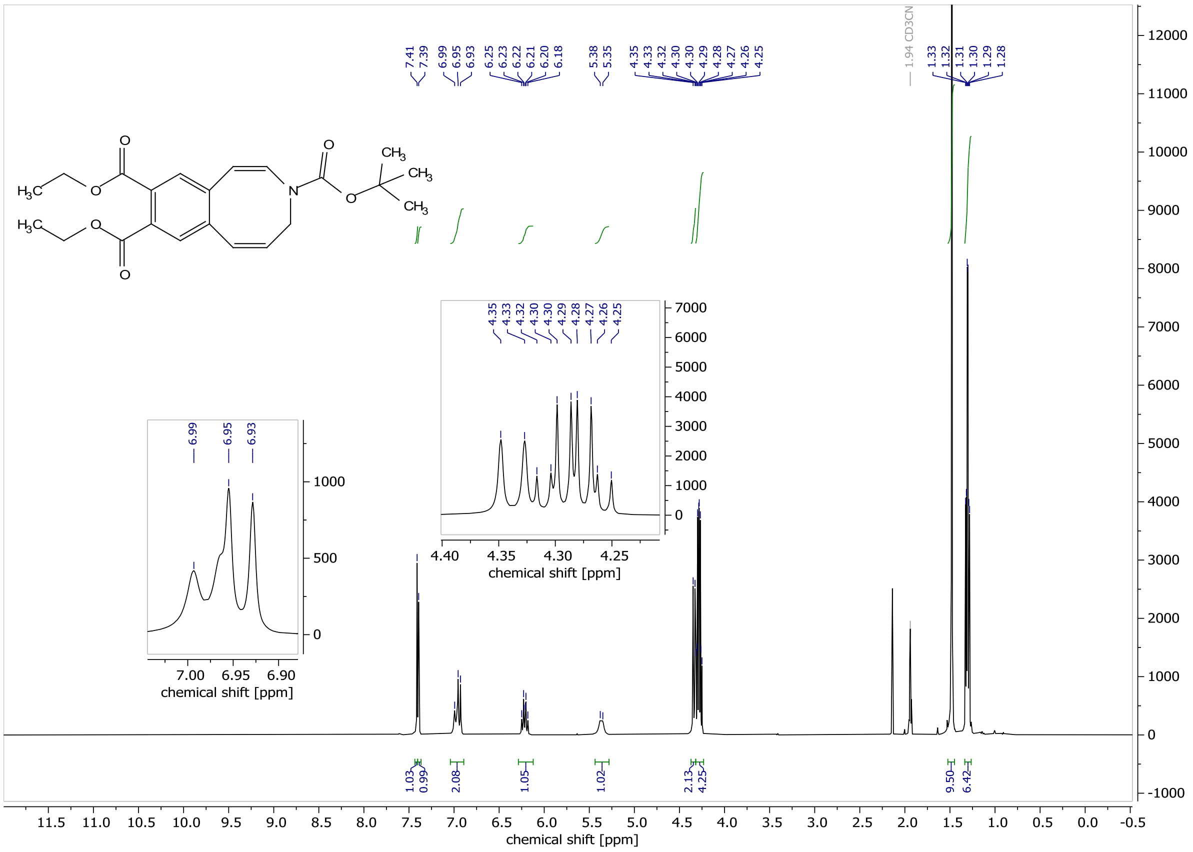

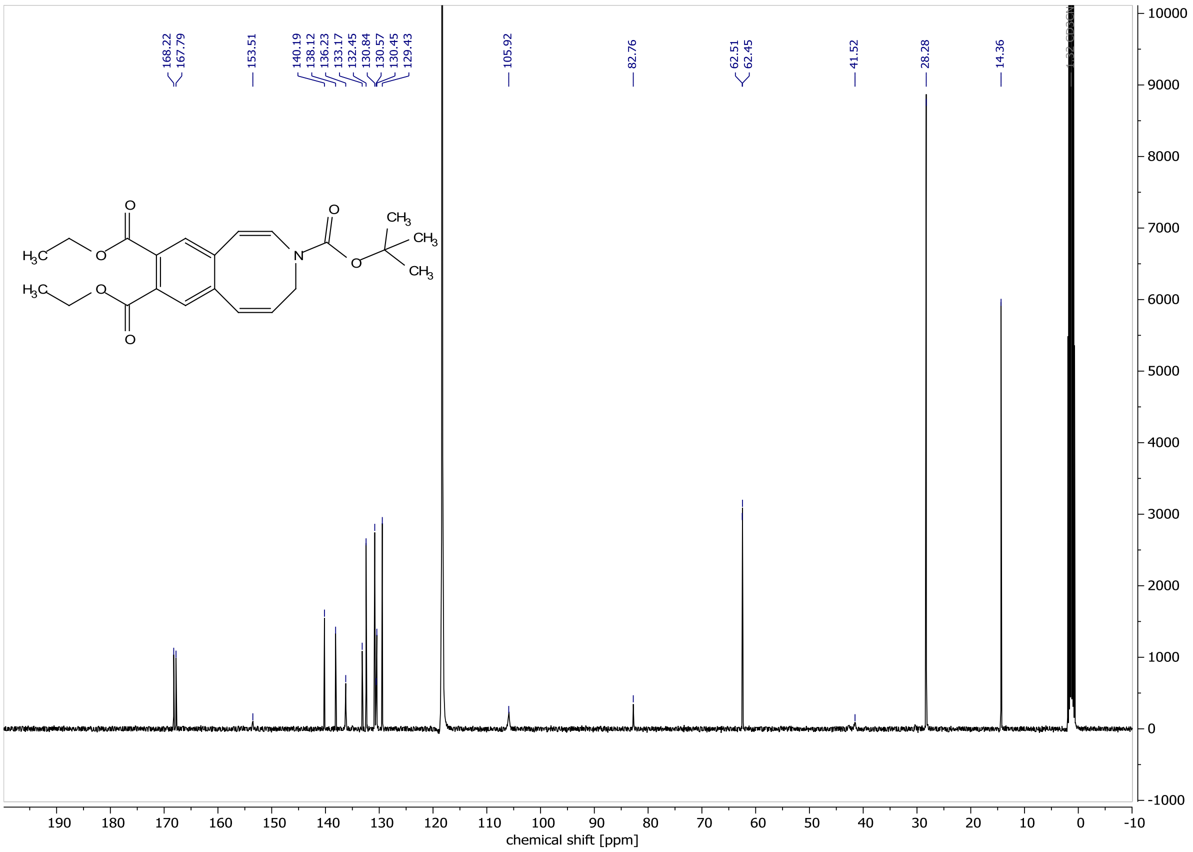

Figure S25. ^{1}H (400 MHz) (top) and $^{13}C\{^{1}H\}$ (101 MHz) (bottom) NMR spectra (CD_3CN) of 3-(*tert*-butyl) 8,9-diethyl (1*Z*,5*Z*)-benzo[*d*]azocine-3,8,9(4*H*)-tricarboxylate (**5j**).

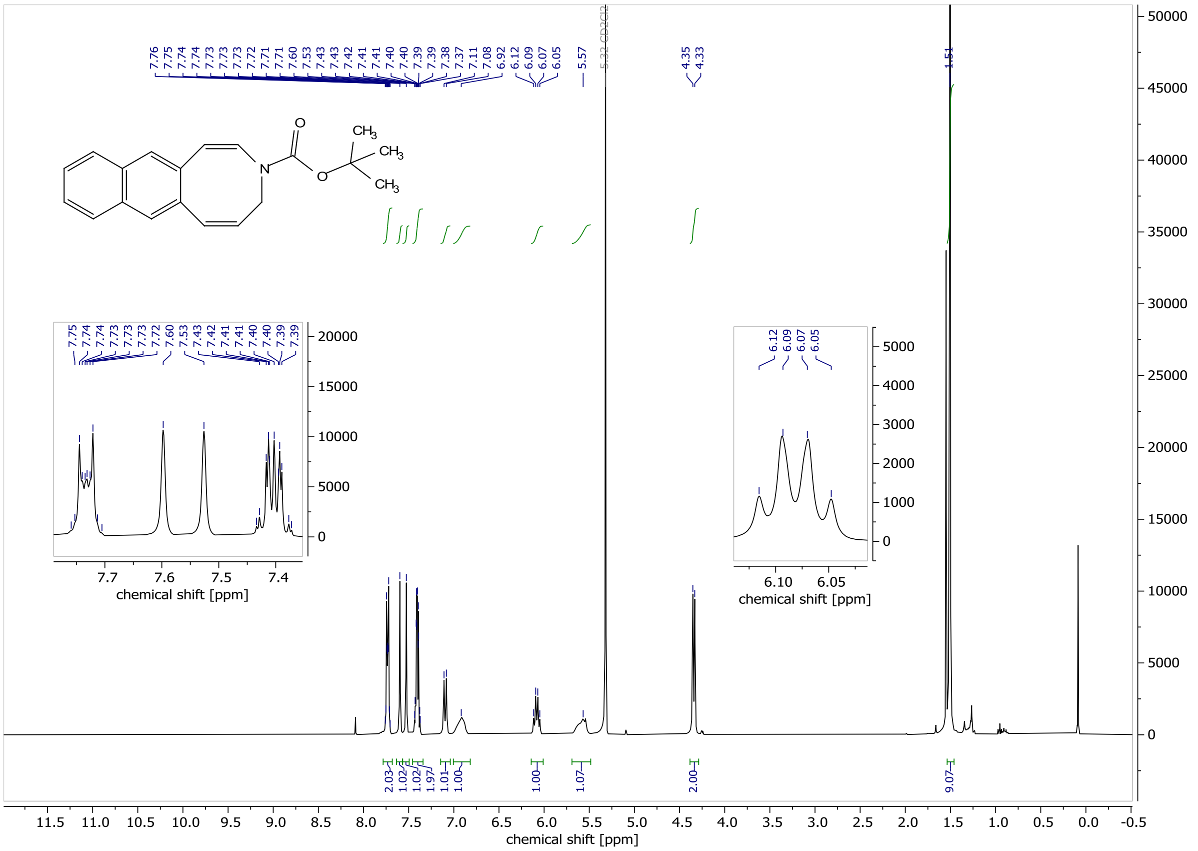

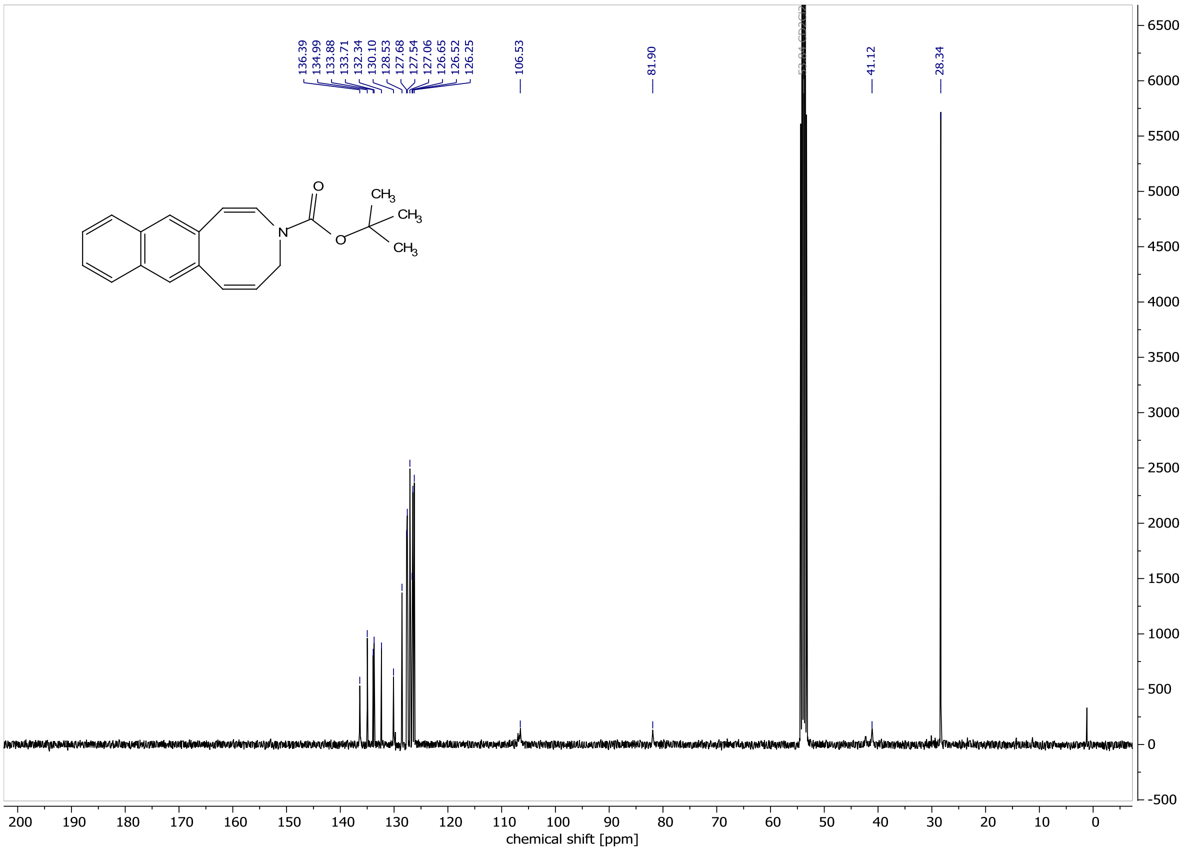

Figure S26. ^{1}H (400 MHz) (top) and $^{13}C\{^{1}H\}$ (101 MHz) (bottom) NMR spectra (CD_3CN) of *tert*-butyl (1*Z*,5*Z*)-naphtho[2,3-*d*]azocine-3(4*H*)-carboxylate (**5k**).

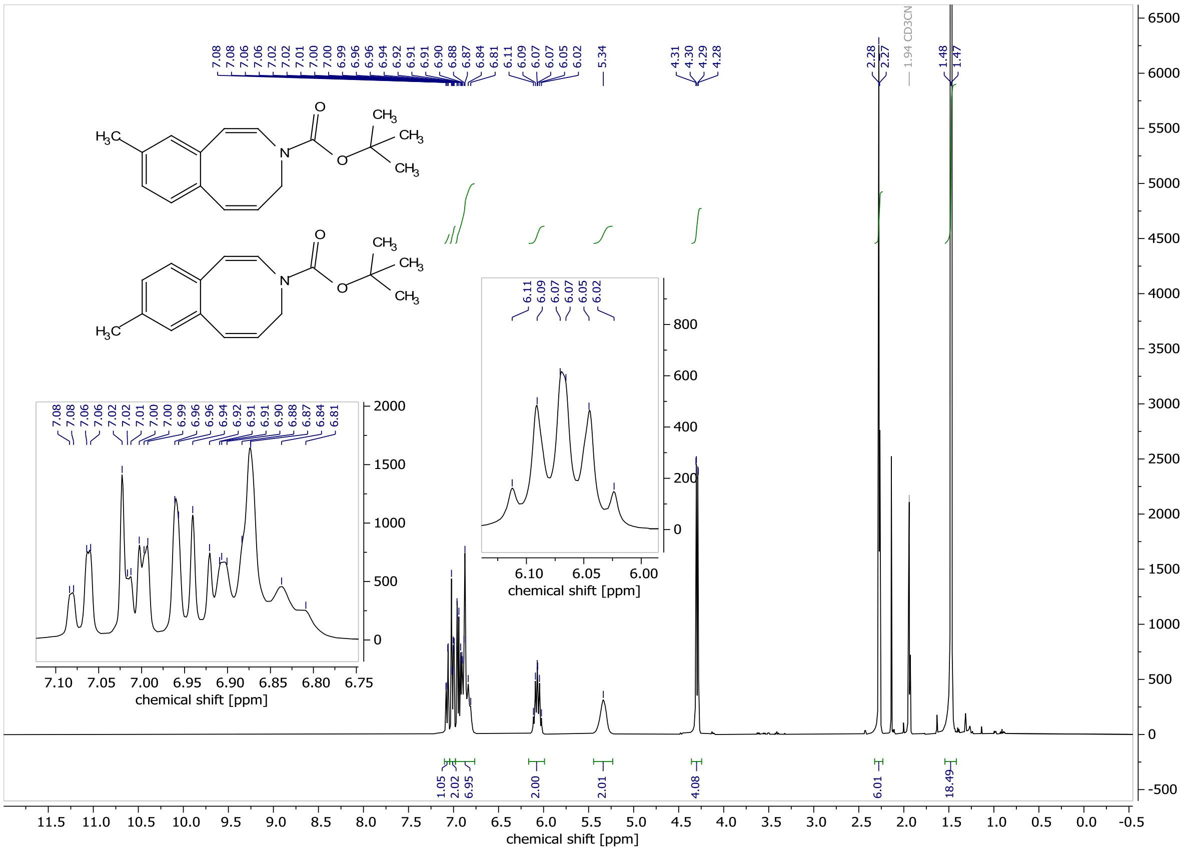

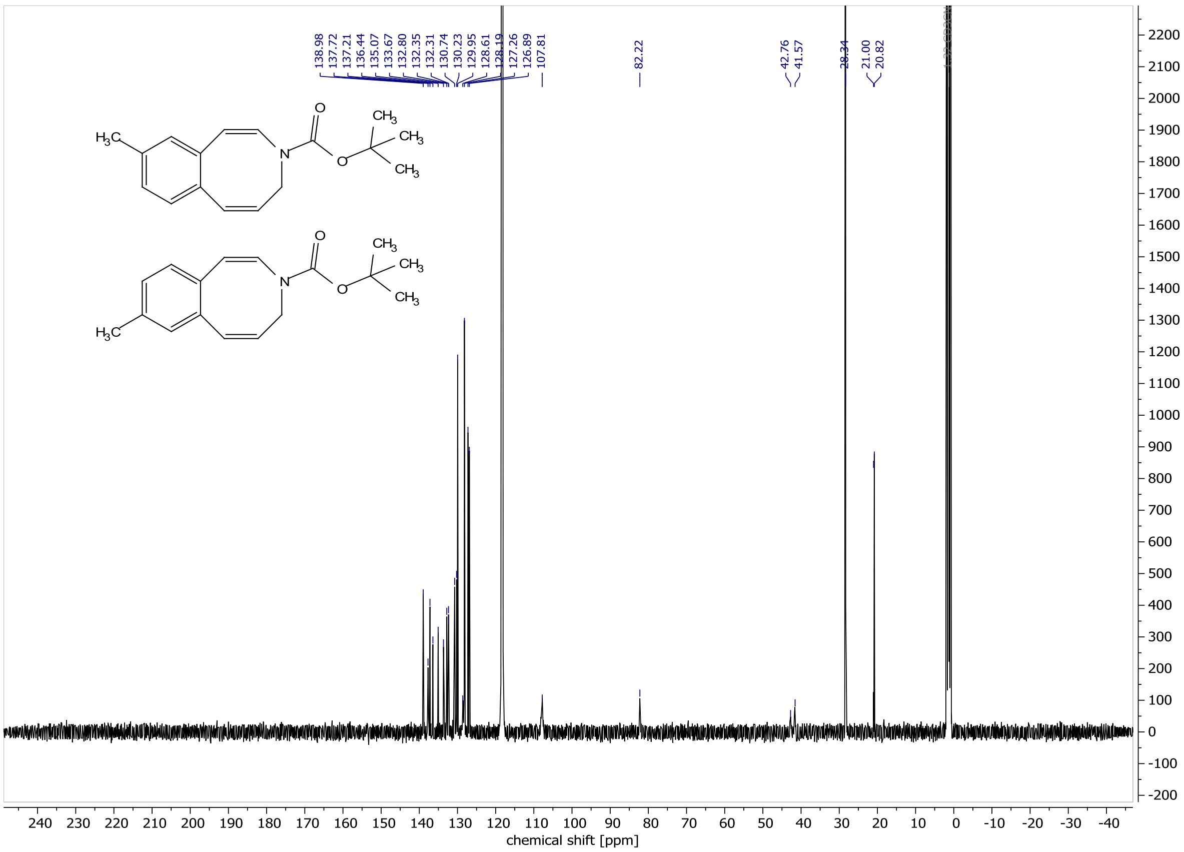

Figure S27. ^{1}H (400 MHz) (top) and ^{13}C{^{1}H} (101 MHz) (bottom) NMR spectra (CD_3CN) of *tert*-butyl (1*Z*,5*Z*)-8/9-methylbenzo[*d*]azocine-3(4*H*)-carboxylate (**5l**).

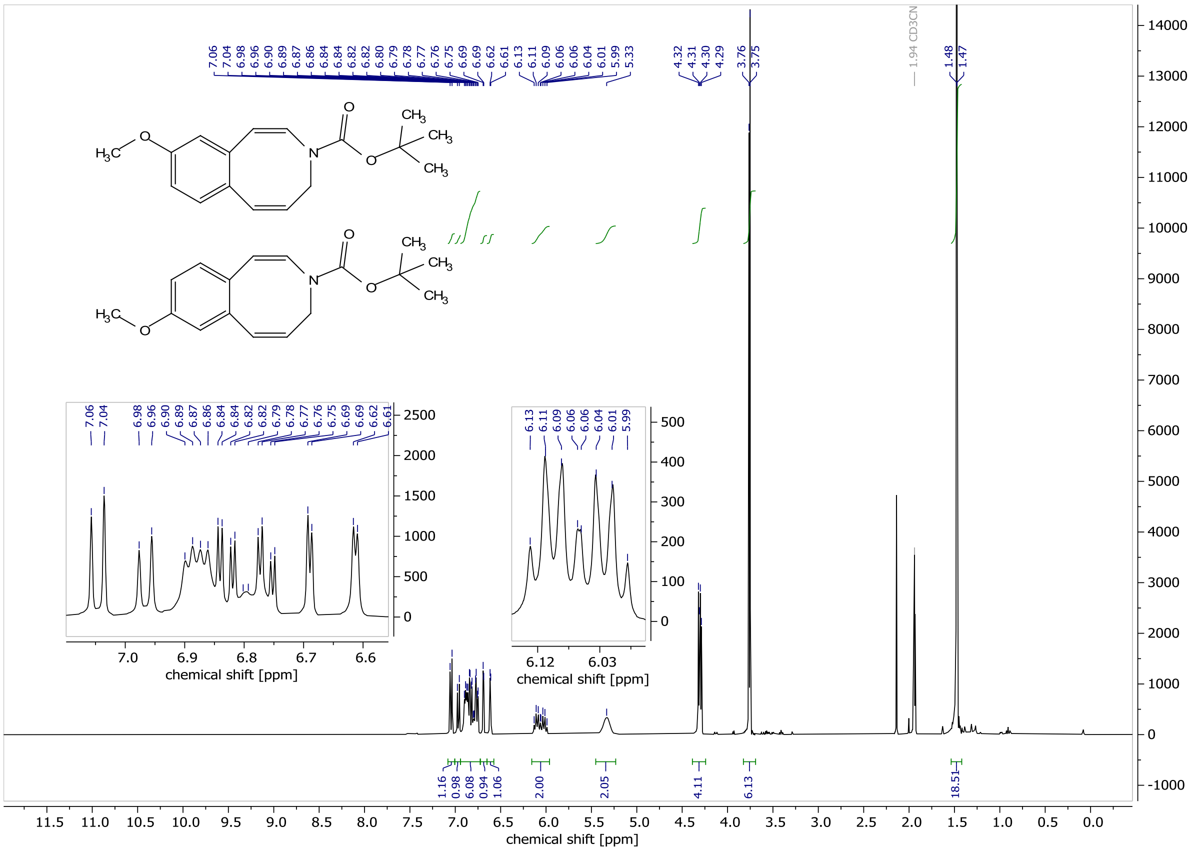

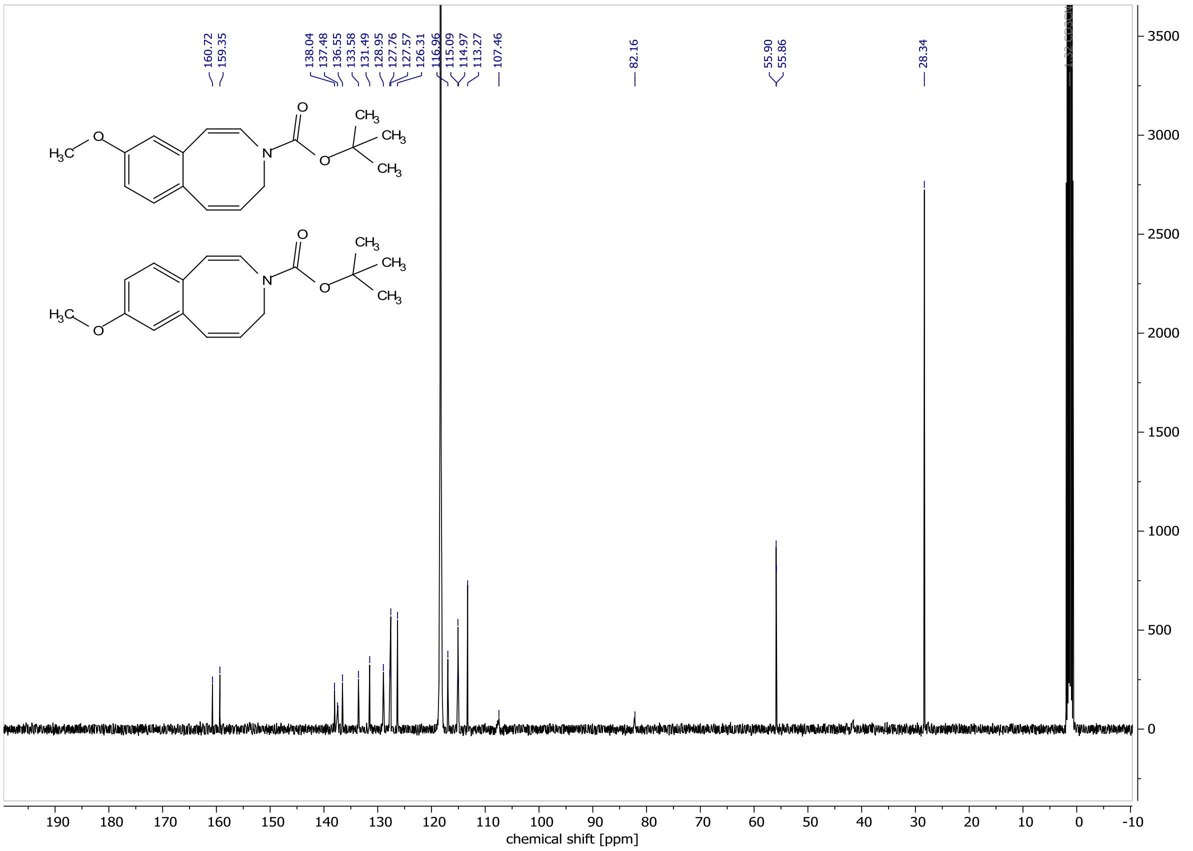

Figure S28. ^{1}H (400 MHz) (top) and $^{13}C\{^{1}H\}$ (101 MHz) (bottom) NMR spectra (CD_3CN) of *tert*-butyl (1*Z*,5*Z*)-8/9-methoxybenzo[*d*]azocine-3(4*H*)-carboxylate (**5m**).

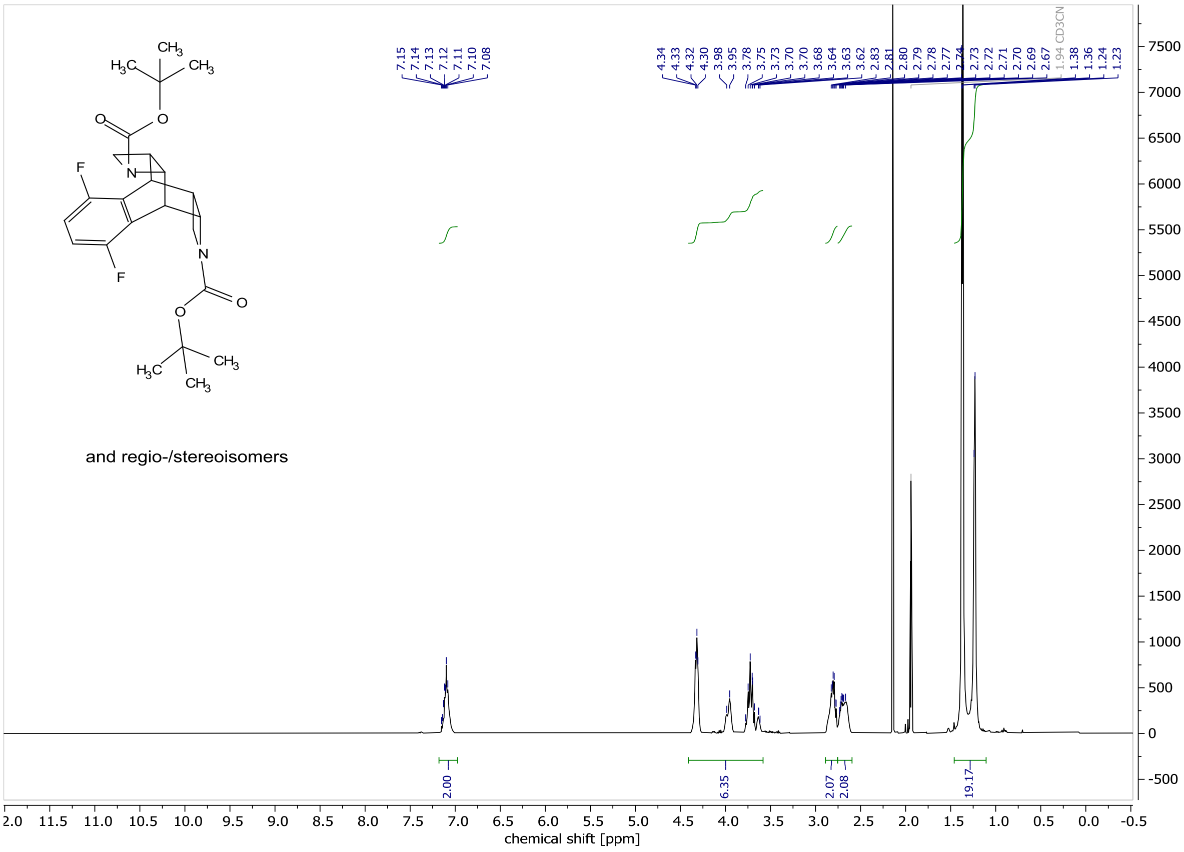

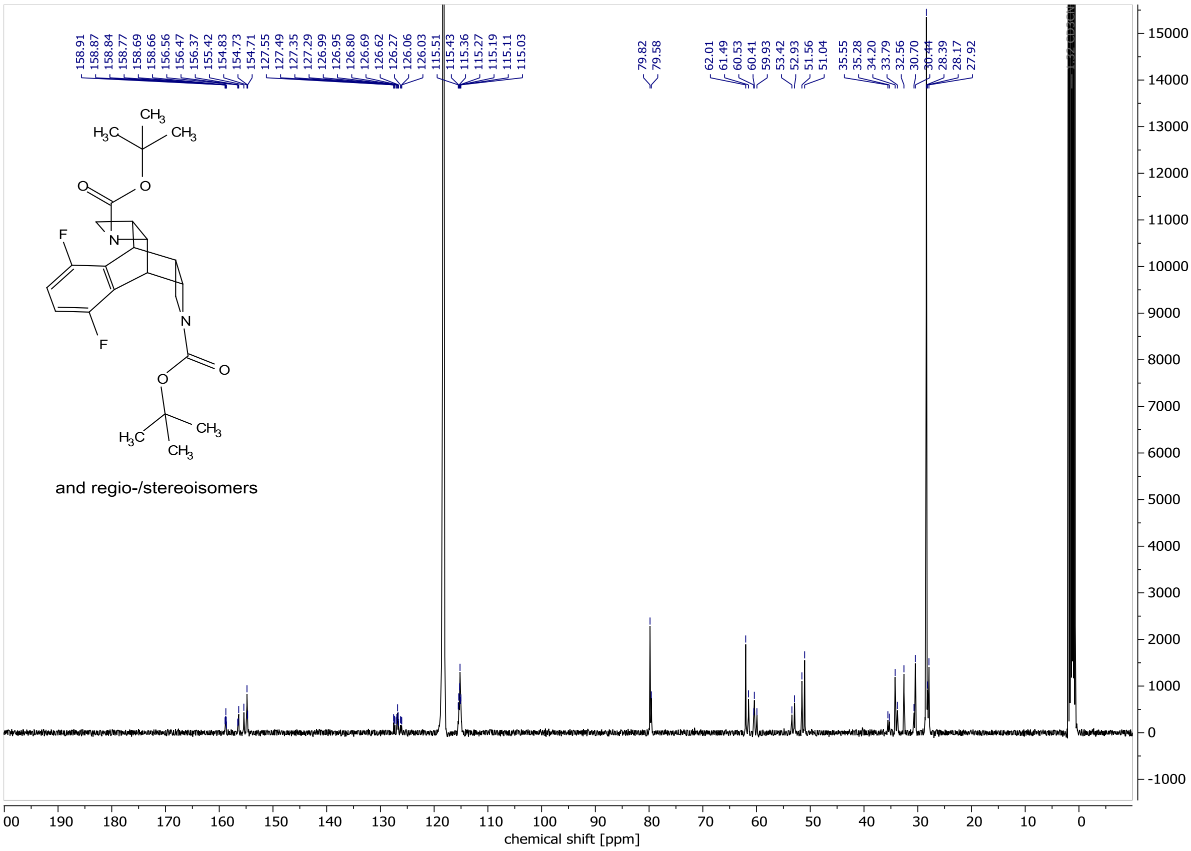

Figure S29. ^{1}H (400 MHz) (top) and ^{13}C{^{1}H} (101 MHz) (bottom) NMR spectra (CD_3CN) of the mixture of double Diels-Alder adducts containing *meso* compound **6**.

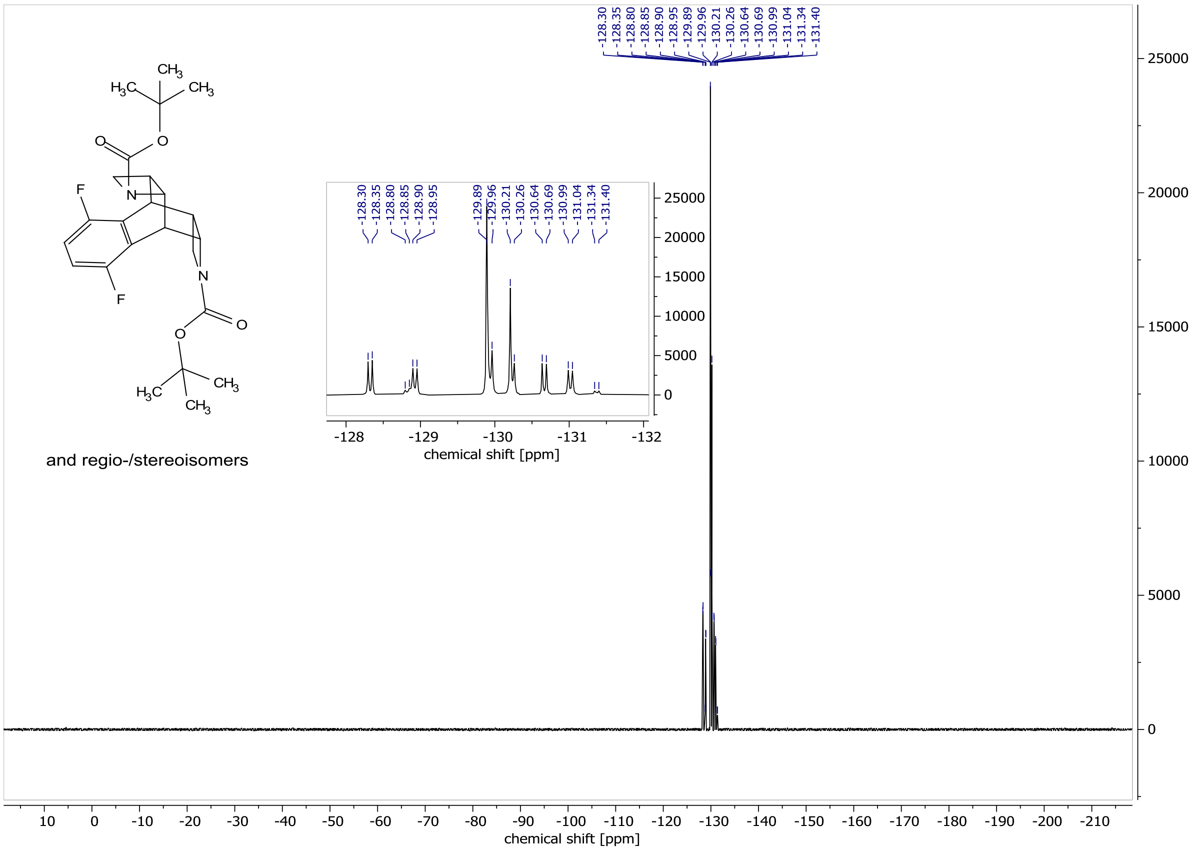

Figure S30. $^{19}F\{^{1}H\}$ (377 MHz) NMR spectrum (CD_3CN) of the mixture of double Diels-Alder adducts containing *meso* compound **6**.

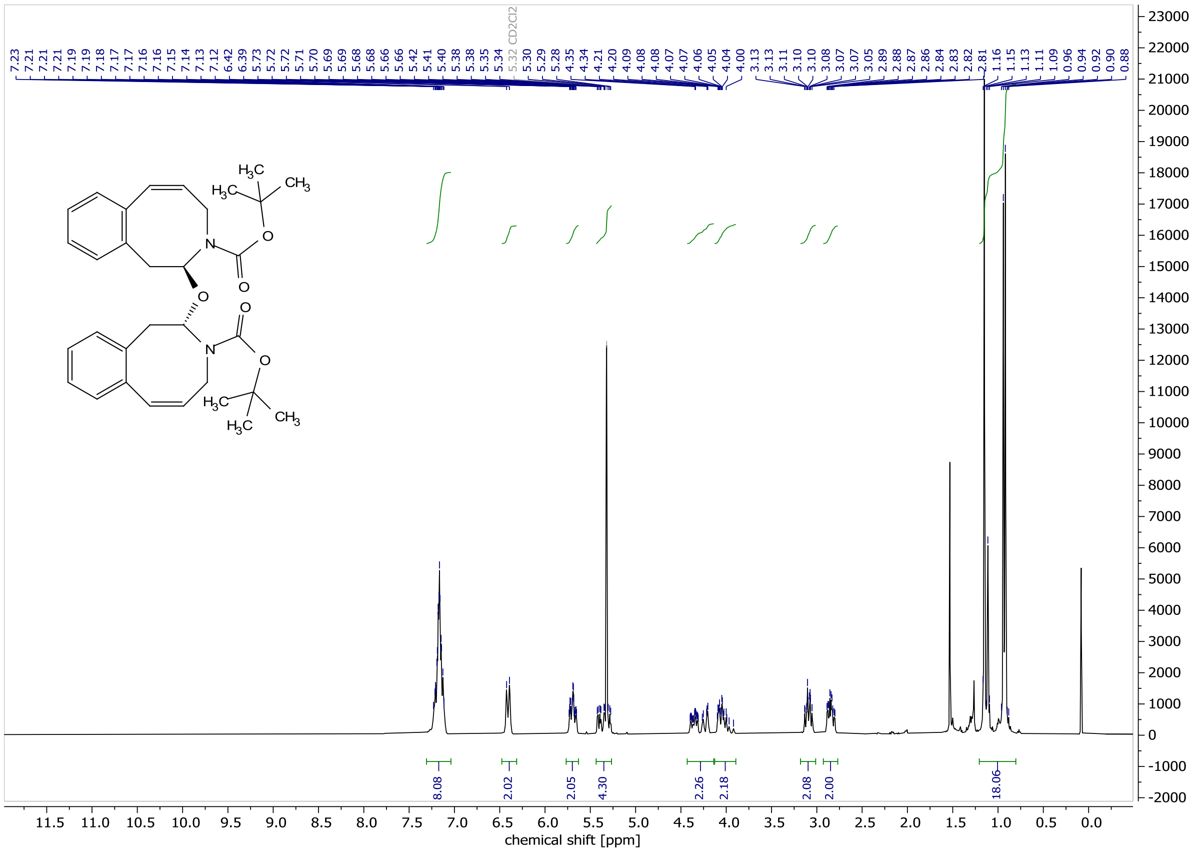

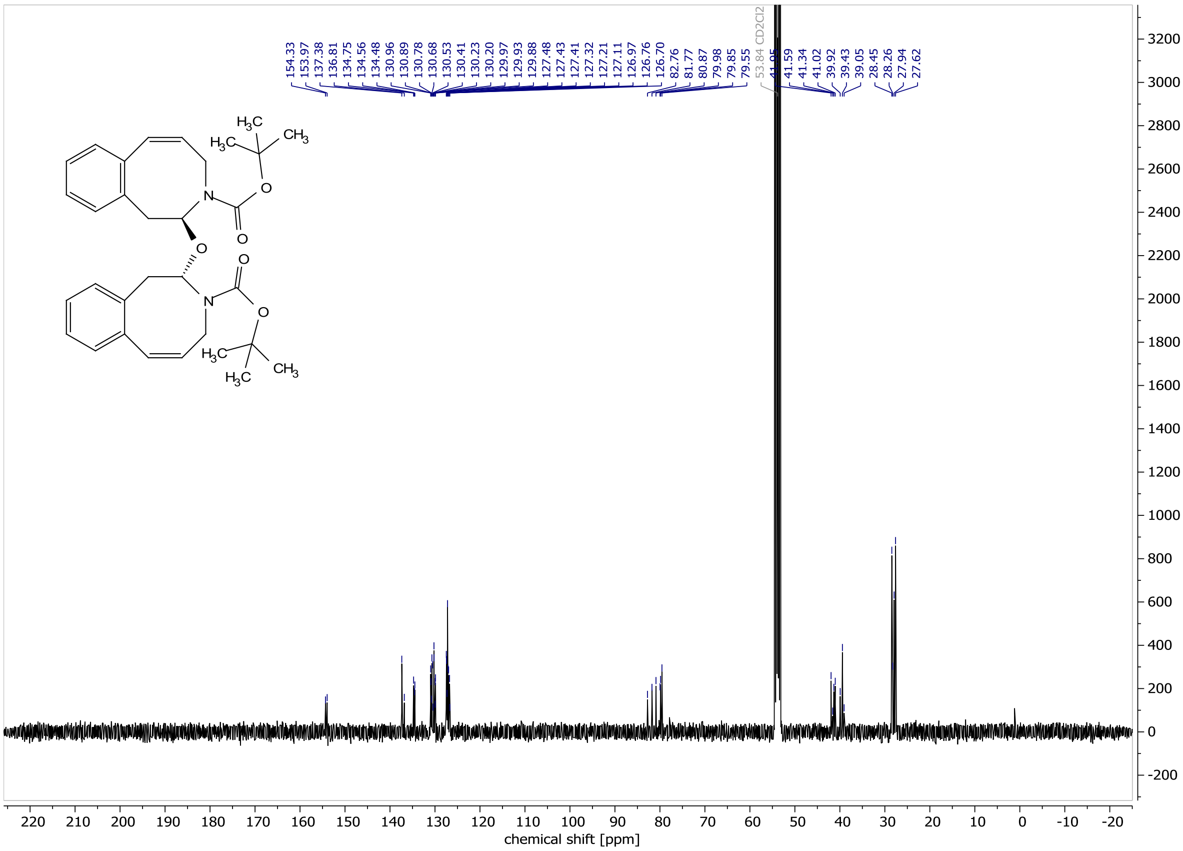

Figure S31. ^{1}H (400 MHz) (top) and $^{13}C\{^{1}H\}$ (101 MHz) (bottom) NMR spectra (CD_2Cl_2) of di-*tert*-butyl 2,2'-oxy(2*S*,2'*S*,5*Z*,5'*Z*)-bis(1,4-dihydrobenzo[*d*]azocine-3(2*H*)-carboxylate) (**7**).

3 XRD Analysis

3.1 General crystallographic experimental details

Suitable single crystals for X-ray structure determination were selected and transferred in protective perfluoropolyether oil on a microscope slide. The selected and mounted crystals were transferred to the cold gas steam on the diffractometer. The diffraction data were obtained at 100 K on a Bruker D8 three circle diffractometer, equipped with a PHOTON 100 CMOS detector and a IµS microfocus sources with Quazar mirror optics (Mo-Kα radiation, λ = 0.71073 Å).

The data obtained were integrated with SAINT and a semi-empirical absorption correction from equivalents with SADABS-2016/2 was applied.[10] The structures were solved by direct methods using SHELXT-2018/2.[11] Structure refinement was done using SHELXT-2018/3.[12] All non-hydrogen atoms were refined anisotropically and C-H hydrogen atoms were positioned at geometrically calculated positions and refined using a riding model. The isotropic displacement parameters of all hydrogen atoms were fixed to 1.2x or 1.5x (CH_3 hydrogens) the U_{eq} value of the atoms they are linked to.

3.2 Crystallographic details of the double Diels-Alder adduct 6

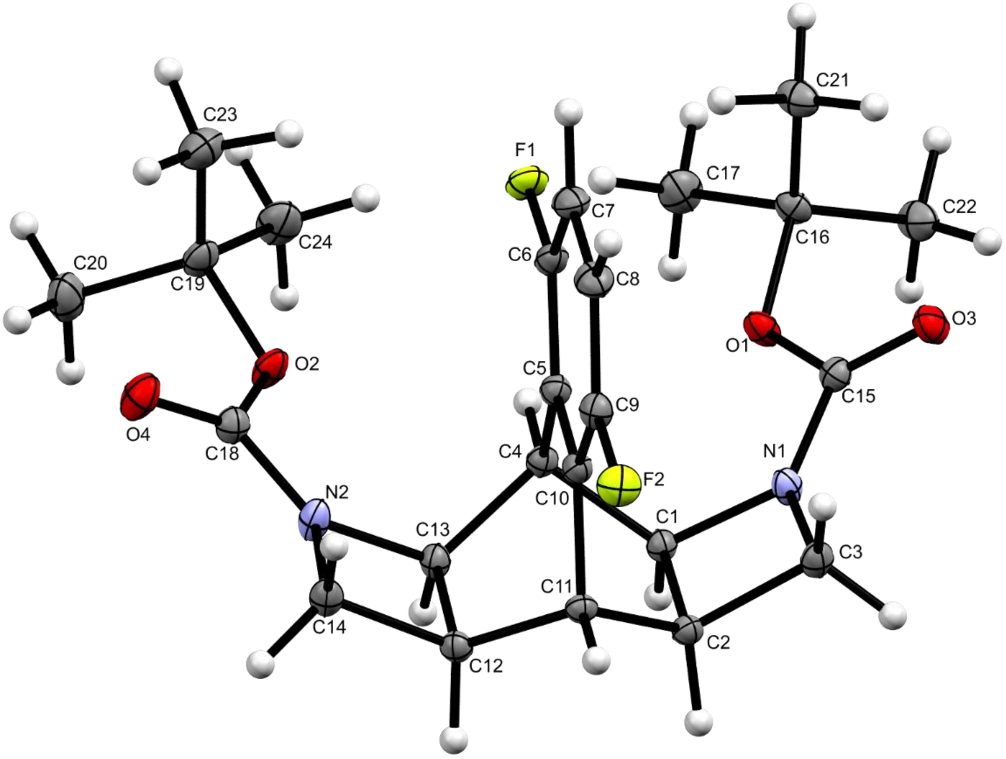

Figure S32. Thermal ellipsoid plot of **6** with the anisotropic displacement parameters drawn at the 50% probability level.

Crystal data and structure refinement for CCDC: 2431433.

Identification code	2431433	
Empirical formula	C24 H30 F2 N2 O4	
Formula weight	448.50	
Temperature	100(2) K	
Wavelength	0.71073 Å	
Crystal system	Monoclinic	
Space group	$P2_1/n$	
Unit cell dimensions	a = 8.1001(4) Å	α= 90°.
	b = 21.6089(10) Å	β= 94.182(2)°.
	c = 12.7007(6) Å	γ = 90°.
Volume	2217.14(18) $Å^3$	
Z	4	
Density (calculated)	1.344 Mg/m^3	
Absorption coefficient	0.102 mm^{-1}	
F(000)	952	
Crystal size	0.464 x 0.325 x 0.190 mm^3	
Theta range for data collection	2.478 to 29.130°.	
Index ranges	-11<=h<=11, -29<=k<=29, -17<=l<=17	
Reflections collected	178212	
Independent reflections	5962 [R(int) = 0.0613]	
Completeness to theta = 25.242°	99.9 %	
Absorption correction	Semi-empirical from equivalents	
Max. and min. transmission	0.7445 and 0.6799	
Refinement method	Full-matrix least-squares on F^2	
Data / restraints / parameters	5962 / 0 / 296	
Goodness-of-fit on F^2	1.054	
Final R indices [I>2sigma(I)]	R1 = 0.0381, wR2 = 0.1003	
R indices (all data)	R1 = 0.0405, wR2 = 0.1030	
Extinction coefficient	0.0243(17)	
Largest diff. peak and hole	0.479 and -0.272 e.$Å^{-3}$	

Atomic coordinates (x 10^4) and equivalent isotropic displacement parameters ($Å^2$x 10^3) for CCDC: 2431433. U(eq) is defined as one third of the trace of the orthogonalized U^{ij} tensor.

	x	y	z	U(eq)
F(1)	1681(1)	3459(1)	3907(1)	18(1)
F(2)	5314(1)	1343(1)	3821(1)	18(1)
O(1)	4044(1)	4009(1)	6052(1)	14(1)
O(2)	3820(1)	4193(1)	1953(1)	16(1)
O(3)	4089(1)	3177(1)	7175(1)	18(1)
O(4)	3840(1)	3491(1)	591(1)	21(1)
N(1)	5976(1)	3300(1)	5955(1)	14(1)
N(2)	5693(1)	3454(1)	2013(1)	16(1)
C(1)	6582(1)	3513(1)	4959(1)	12(1)
C(2)	7592(1)	2897(1)	4925(1)	12(1)
C(3)	6849(1)	2703(1)	5969(1)	14(1)
C(4)	5316(1)	3565(1)	3993(1)	12(1)
C(5)	4309(1)	2981(1)	3913(1)	12(1)
C(6)	2602(1)	2931(1)	3899(1)	14(1)
C(7)	1789(1)	2369(1)	3874(1)	17(1)
C(8)	2716(1)	1828(1)	3849(1)	17(1)
C(9)	4419(1)	1873(1)	3845(1)	13(1)
C(10)	5237(1)	2436(1)	3874(1)	11(1)
C(11)	7078(1)	2536(1)	3905(1)	11(1)
C(12)	7461(1)	2978(1)	3000(1)	12(1)
C(13)	6442(1)	3589(1)	3070(1)	12(1)
C(14)	6598(1)	2875(1)	1881(1)	15(1)
C(15)	4638(1)	3469(1)	6459(1)	12(1)
C(16)	2582(1)	4303(1)	6459(1)	14(1)
C(17)	2304(1)	4846(1)	5707(1)	21(1)
C(18)	4390(1)	3695(1)	1438(1)	14(1)
C(19)	2349(1)	4531(1)	1523(1)	17(1)
C(20)	2704(1)	4842(1)	490(1)	25(1)
C(21)	1096(1)	3870(1)	6372(1)	18(1)
C(22)	2994(1)	4523(1)	7586(1)	19(1)
C(23)	856(1)	4102(1)	1404(1)	24(1)
C(24)	2116(1)	5012(1)	2373(1)	23(1)

Bond lengths [Å] and angles [°] for CCDC: 2431433.

F(1)-C(6)	1.3631(10)	C(12)-C(13)	1.5630(12)
F(2)-C(9)	1.3570(10)	C(12)-H(12)	1.0000
O(1)-C(15)	1.3522(10)	C(13)-H(13)	1.0000
O(1)-C(16)	1.4697(10)	C(14)-H(14A)	0.9900
O(2)-C(18)	1.3575(11)	C(14)-H(14B)	0.9900
O(2)-C(19)	1.4682(10)	C(16)-C(17)	1.5195(13)
O(3)-C(15)	1.2161(11)	C(16)-C(21)	1.5216(12)
O(4)-C(18)	1.2161(11)	C(16)-C(22)	1.5218(12)
N(1)-C(15)	1.3483(11)	C(17)-H(17A)	0.9800
N(1)-C(1)	1.4643(11)	C(17)-H(17B)	0.9800
N(1)-C(3)	1.4710(11)	C(17)-H(17C)	0.9800
N(2)-C(18)	1.3436(11)	C(19)-C(24)	1.5200(13)
N(2)-C(13)	1.4612(11)	C(19)-C(20)	1.5202(14)
N(2)-C(14)	1.4671(11)	C(19)-C(23)	1.5234(14)
C(1)-C(4)	1.5450(12)	C(20)-H(20A)	0.9800
C(1)-C(2)	1.5648(11)	C(20)-H(20B)	0.9800
C(1)-H(1)	1.0000	C(20)-H(20C)	0.9800
C(2)-C(11)	1.5431(11)	C(21)-H(21A)	0.9800
C(2)-C(3)	1.5536(12)	C(21)-H(21B)	0.9800
C(2)-H(2)	1.0000	C(21)-H(21C)	0.9800
C(3)-H(3A)	0.9900	C(22)-H(22A)	0.9800
C(3)-H(3B)	0.9900	C(22)-H(22B)	0.9800
C(4)-C(5)	1.5022(12)	C(22)-H(22C)	0.9800
C(4)-C(13)	1.5385(12)	C(23)-H(23A)	0.9800
C(4)-H(4)	1.0000	C(23)-H(23B)	0.9800
C(5)-C(6)	1.3854(12)	C(23)-H(23C)	0.9800
C(5)-C(10)	1.4005(11)	C(24)-H(24A)	0.9800
C(6)-C(7)	1.3803(13)	C(24)-H(24B)	0.9800
C(7)-C(8)	1.3909(13)	C(24)-H(24C)	0.9800
C(7)-H(7)	0.9500	C(15)-O(1)-C(16)	120.78(7)
C(8)-C(9)	1.3834(12)	C(18)-O(2)-C(19)	120.73(7)
C(8)-H(8)	0.9500	C(15)-N(1)-C(1)	131.08(7)
C(9)-C(10)	1.3837(12)	C(15)-N(1)-C(3)	129.34(7)
C(10)-C(11)	1.5039(11)	C(1)-N(1)-C(3)	95.45(6)
C(11)-C(12)	1.5434(12)	C(18)-N(2)-C(13)	132.81(8)
C(11)-H(11)	1.0000	C(18)-N(2)-C(14)	130.37(8)
C(12)-C(14)	1.5540(12)	C(13)-N(2)-C(14)	95.59(6)

N(1)-C(1)-C(4)	117.62(7)	C(9)-C(10)-C(5)	118.85(8)
N(1)-C(1)-C(2)	88.02(6)	C(9)-C(10)-C(11)	126.76(8)
C(4)-C(1)-C(2)	110.98(7)	C(5)-C(10)-C(11)	114.36(7)
N(1)-C(1)-H(1)	112.6	C(10)-C(11)-C(2)	107.51(7)
C(4)-C(1)-H(1)	112.6	C(10)-C(11)-C(12)	108.79(7)
C(2)-C(1)-H(1)	112.6	C(2)-C(11)-C(12)	104.76(7)
C(11)-C(2)-C(3)	118.82(7)	C(10)-C(11)-H(11)	111.8
C(11)-C(2)-C(1)	110.14(6)	C(2)-C(11)-H(11)	111.8
C(3)-C(2)-C(1)	88.28(6)	C(12)-C(11)-H(11)	111.8
C(11)-C(2)-H(2)	112.4	C(11)-C(12)-C(14)	119.40(7)
C(3)-C(2)-H(2)	112.4	C(11)-C(12)-C(13)	110.21(7)
C(1)-C(2)-H(2)	112.4	C(14)-C(12)-C(13)	88.20(6)
N(1)-C(3)-C(2)	88.21(6)	C(11)-C(12)-H(12)	112.2
N(1)-C(3)-H(3A)	113.9	C(14)-C(12)-H(12)	112.2
C(2)-C(3)-H(3A)	113.9	C(13)-C(12)-H(12)	112.2
N(1)-C(3)-H(3B)	113.9	N(2)-C(13)-C(4)	117.61(7)
C(2)-C(3)-H(3B)	113.9	N(2)-C(13)-C(12)	88.04(6)
H(3A)-C(3)-H(3B)	111.2	C(4)-C(13)-C(12)	111.09(7)
C(5)-C(4)-C(13)	109.01(7)	N(2)-C(13)-H(13)	112.6
C(5)-C(4)-C(1)	108.55(7)	C(4)-C(13)-H(13)	112.6
C(13)-C(4)-C(1)	102.20(7)	C(12)-C(13)-H(13)	112.6
C(5)-C(4)-H(4)	112.2	N(2)-C(14)-C(12)	88.17(6)
C(13)-C(4)-H(4)	112.2	N(2)-C(14)-H(14A)	114.0
C(1)-C(4)-H(4)	112.2	C(12)-C(14)-H(14A)	114.0
C(6)-C(5)-C(10)	118.16(8)	N(2)-C(14)-H(14B)	114.0
C(6)-C(5)-C(4)	127.06(8)	C(12)-C(14)-H(14B)	114.0
C(10)-C(5)-C(4)	114.76(7)	H(14A)-C(14)-H(14B)	111.2
F(1)-C(6)-C(7)	118.38(8)	O(3)-C(15)-N(1)	124.42(8)
F(1)-C(6)-C(5)	118.76(8)	O(3)-C(15)-O(1)	126.41(8)
C(7)-C(6)-C(5)	122.86(8)	N(1)-C(15)-O(1)	109.17(7)
C(6)-C(7)-C(8)	118.86(8)	O(1)-C(16)-C(17)	101.43(7)
C(6)-C(7)-H(7)	120.6	O(1)-C(16)-C(21)	111.26(7)
C(8)-C(7)-H(7)	120.6	C(17)-C(16)-C(21)	110.38(8)
C(9)-C(8)-C(7)	118.73(8)	O(1)-C(16)-C(22)	109.78(7)
C(9)-C(8)-H(8)	120.6	C(17)-C(16)-C(22)	111.24(8)
C(7)-C(8)-H(8)	120.6	C(21)-C(16)-C(22)	112.26(7)
F(2)-C(9)-C(8)	118.35(8)	C(16)-C(17)-H(17A)	109.5
F(2)-C(9)-C(10)	119.12(8)	C(16)-C(17)-H(17B)	109.5
C(8)-C(9)-C(10)	122.52(8)	H(17A)-C(17)-H(17B)	109.5

C(16)-C(17)-H(17C)	109.5	C(16)-C(21)-H(21C)	109.5
H(17A)-C(17)-H(17C)	109.5	H(21A)-C(21)-H(21C)	109.5
H(17B)-C(17)-H(17C)	109.5	H(21B)-C(21)-H(21C)	109.5
O(4)-C(18)-N(2)	124.28(8)	C(16)-C(22)-H(22A)	109.5
O(4)-C(18)-O(2)	126.67(8)	C(16)-C(22)-H(22B)	109.5
N(2)-C(18)-O(2)	109.05(7)	H(22A)-C(22)-H(22B)	109.5
O(2)-C(19)-C(24)	102.49(7)	C(16)-C(22)-H(22C)	109.5
O(2)-C(19)-C(20)	109.94(8)	H(22A)-C(22)-H(22C)	109.5
C(24)-C(19)-C(20)	110.65(8)	H(22B)-C(22)-H(22C)	109.5
O(2)-C(19)-C(23)	110.54(8)	C(19)-C(23)-H(23A)	109.5
C(24)-C(19)-C(23)	110.36(8)	C(19)-C(23)-H(23B)	109.5
C(20)-C(19)-C(23)	112.42(8)	H(23A)-C(23)-H(23B)	109.5
C(19)-C(20)-H(20A)	109.5	C(19)-C(23)-H(23C)	109.5
C(19)-C(20)-H(20B)	109.5	H(23A)-C(23)-H(23C)	109.5
H(20A)-C(20)-H(20B)	109.5	H(23B)-C(23)-H(23C)	109.5
C(19)-C(20)-H(20C)	109.5	C(19)-C(24)-H(24A)	109.5
H(20A)-C(20)-H(20C)	109.5	C(19)-C(24)-H(24B)	109.5
H(20B)-C(20)-H(20C)	109.5	H(24A)-C(24)-H(24B)	109.5
C(16)-C(21)-H(21A)	109.5	C(19)-C(24)-H(24C)	109.5
C(16)-C(21)-H(21B)	109.5	H(24A)-C(24)-H(24C)	109.5
H(21A)-C(21)-H(21B)	109.5	H(24B)-C(24)-H(24C)	109.5

Anisotropic displacement parameters ($Å^2$x 10^3) for CCDC: 2431433. The anisotropic displacement factor exponent takes the form: $-2\pi^2[h^2 a^{*2}U^{11} + ... + 2 h k a^* b^* U^{12}]$

	U^{11}	U^{22}	U^{33}	U^{23}	U^{13}	U^{12}
F(1)	13(1)	20(1)	19(1)	-2(1)	0(1)	7(1)
F(2)	20(1)	11(1)	22(1)	-1(1)	2(1)	2(1)
O(1)	16(1)	12(1)	16(1)	2(1)	6(1)	4(1)
O(2)	17(1)	16(1)	15(1)	-3(1)	-4(1)	7(1)
O(3)	18(1)	18(1)	17(1)	6(1)	5(1)	1(1)
O(4)	25(1)	21(1)	17(1)	-5(1)	-5(1)	4(1)
N(1)	16(1)	12(1)	12(1)	2(1)	3(1)	4(1)
N(2)	21(1)	15(1)	11(1)	-2(1)	-2(1)	7(1)
C(1)	13(1)	12(1)	10(1)	0(1)	1(1)	1(1)
C(2)	11(1)	13(1)	12(1)	0(1)	0(1)	1(1)
C(3)	16(1)	13(1)	12(1)	1(1)	1(1)	4(1)
C(4)	12(1)	11(1)	11(1)	0(1)	0(1)	2(1)
C(5)	11(1)	13(1)	10(1)	-1(1)	0(1)	1(1)
C(6)	12(1)	17(1)	12(1)	-2(1)	0(1)	4(1)
C(7)	11(1)	23(1)	16(1)	-2(1)	0(1)	-1(1)
C(8)	15(1)	18(1)	17(1)	-2(1)	1(1)	-4(1)
C(9)	16(1)	12(1)	12(1)	-1(1)	1(1)	1(1)
C(10)	11(1)	13(1)	10(1)	-1(1)	1(1)	1(1)
C(11)	10(1)	11(1)	12(1)	0(1)	1(1)	2(1)
C(12)	12(1)	12(1)	12(1)	0(1)	2(1)	2(1)
C(13)	15(1)	12(1)	10(1)	1(1)	0(1)	2(1)
C(14)	18(1)	14(1)	12(1)	-1(1)	1(1)	5(1)
C(15)	13(1)	12(1)	12(1)	-1(1)	0(1)	0(1)
C(16)	14(1)	13(1)	15(1)	-2(1)	3(1)	2(1)
C(17)	24(1)	16(1)	24(1)	3(1)	4(1)	7(1)
C(18)	16(1)	13(1)	13(1)	0(1)	1(1)	2(1)
C(19)	16(1)	18(1)	15(1)	1(1)	-1(1)	7(1)
C(20)	29(1)	27(1)	19(1)	7(1)	3(1)	9(1)
C(21)	16(1)	19(1)	20(1)	-6(1)	4(1)	-2(1)
C(22)	18(1)	22(1)	17(1)	-7(1)	2(1)	0(1)
C(23)	19(1)	27(1)	25(1)	2(1)	-4(1)	2(1)
C(24)	24(1)	24(1)	22(1)	-5(1)	1(1)	11(1)

Hydrogen coordinates (x 10^4) and isotropic displacement parameters ($Å^2$x 10^{3}) for CCDC: 2431433.

	x	y	z	U(eq)
H(1)	7309	3886	5051	14
H(2)	8814	2967	5018	14
H(3A)	6105	2339	5896	17
H(3B)	7682	2652	6572	17
H(4)	4607	3943	4025	14
H(7)	618	2352	3874	20
H(8)	2189	1435	3835	20
H(11)	7693	2136	3868	13
H(12)	8674	3058	2980	15
H(13)	7156	3967	3087	15
H(14A)	7362	2887	1307	18
H(14B)	5883	2503	1823	18
H(17A)	2008	4691	4993	32
H(17B)	3320	5092	5707	32
H(17C)	1404	5105	5937	32
H(20A)	2861	4525	-46	38
H(20B)	3710	5093	595	38
H(20C)	1769	5108	253	38
H(21A)	95	4102	6510	27
H(21B)	1264	3536	6890	27
H(21C)	971	3694	5659	27
H(22A)	3922	4815	7600	29
H(22B)	3301	4167	8037	29
H(22C)	2026	4728	7847	29
H(23A)	-148	4347	1238	36
H(23B)	755	3876	2065	36
H(23C)	999	3807	832	36
H(24A)	3125	5261	2486	35
H(24B)	1890	4804	3033	35
H(24C)	1183	5282	2149	35

3.3 Crystallographic details of the hemiaminal ether 7

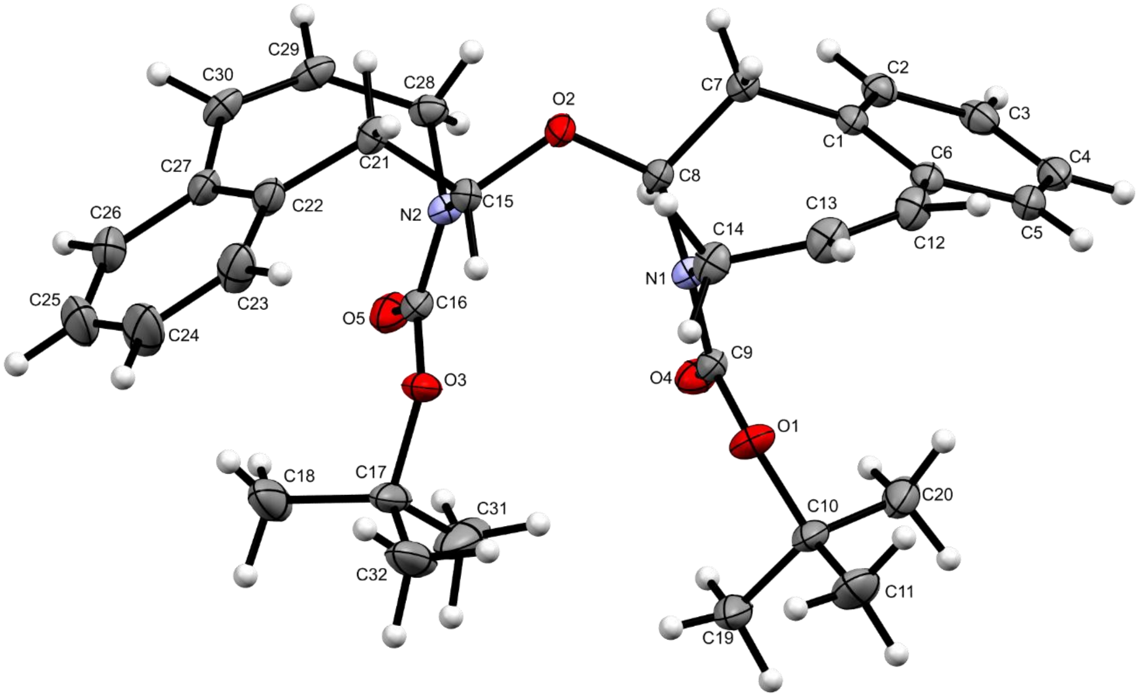

Figure S33. Thermal ellipsoid plot of **7** with the anisotropic displacement parameters drawn at the 50% probability level.

A solvent accessible void at 0.000 -0.021 0.000 with a volume of 166 Å^3 and an equivalent of 40 electrons was identified by SQUEEZE[13] as implemented in PLATON.[14–17] The disordered solvent was modelled by SQUEEZE and the model was included as a .fab file into the refinement. The Flack parameter was determined to be 0.3(2), indicating that the absolute structure is likely correct, although with some degree of uncertainty.

Crystal data and structure refinement for CCDC: 2431435.

Identification code	2431435	
Empirical formula	C32 H40 N2 O5	
Formula weight	532.66	
Temperature	100(2) K	
Wavelength	0.71073 Å	
Crystal system	Monoclinic	
Space group	$P2_1$	
Unit cell dimensions	a = 12.3406(4) Å	α= 90°.
	b = 6.6371(3) Å	β= 90.069(2)°.
	c = 19.2425(8) Å	γ = 90°.
Volume	1576.07(11) $Å^3$	
Z	2	
Density (calculated)	1.122 Mg/m^3	
Absorption coefficient	0.075 mm^{-1}	
F(000)	572	
Crystal size	0.292 x 0.226 x 0.058 mm^3	
Theta range for data collection	1.962 to 27.482°.	
Index ranges	-16<=h<=16, -8<=k<=8, -24<=l<=24	
Reflections collected	64991	
Independent reflections	7223 [R(int) = 0.0599]	
Completeness to theta = 25.242°	99.9 %	
Absorption correction	Semi-empirical from equivalents	
Max. and min. transmission	0.7457 and 0.6489	
Refinement method	Full-matrix least-squares on F^2	
Data / restraints / parameters	7223 / 1 / 359	
Goodness-of-fit on F^2	1.066	
Final R indices [I>2sigma(I)]	R1 = 0.0363, wR2 = 0.0925	
R indices (all data)	R1 = 0.0372, wR2 = 0.0934	
Absolute structure parameter	0.3(2)	
Extinction coefficient	0.052(5)	
Largest diff. peak and hole	0.280 and -0.211 e.$Å^{-3}$	

Atomic coordinates (x 10^4) and equivalent isotropic displacement parameters ($Å^2$x 10^3) for CCDC: 2431435. U(eq) is defined as one third of the trace of the orthogonalized U^{ij} tensor.

	x	y	z	U(eq)
O(1)	1676(1)	6693(2)	2886(1)	26(1)
O(2)	5127(1)	6041(2)	3643(1)	20(1)
O(3)	4435(1)	3409(2)	1927(1)	25(1)
O(4)	2408(1)	3617(2)	3120(1)	24(1)
O(5)	5040(1)	358(2)	2307(1)	27(1)
N(1)	3238(1)	6537(2)	3461(1)	17(1)
N(2)	5393(1)	3225(2)	2909(1)	18(1)
C(1)	2934(1)	5493(2)	4946(1)	18(1)
C(2)	2846(1)	3656(2)	5295(1)	21(1)
C(3)	1880(1)	3077(3)	5608(1)	23(1)
C(4)	992(1)	4347(3)	5585(1)	24(1)
C(5)	1072(1)	6188(3)	5247(1)	22(1)
C(6)	2031(1)	6779(2)	4912(1)	18(1)
C(7)	4010(1)	6058(2)	4628(1)	19(1)
C(8)	4063(1)	5495(2)	3859(1)	17(1)
C(9)	2430(1)	5444(2)	3152(1)	18(1)
C(10)	592(1)	5917(3)	2706(1)	28(1)
C(11)	-20(2)	7852(4)	2541(1)	40(1)
C(12)	2023(1)	8821(2)	4605(1)	24(1)
C(13)	2503(1)	9631(2)	4053(1)	25(1)
C(14)	3210(1)	8730(2)	3497(1)	22(1)
C(15)	5406(1)	5412(2)	2957(1)	18(1)
C(16)	4954(1)	2175(2)	2366(1)	20(1)
C(17)	3935(2)	2618(4)	1288(1)	36(1)
C(18)	4809(2)	1755(5)	813(1)	53(1)
C(19)	645(1)	4565(4)	2069(1)	39(1)
C(20)	104(1)	4840(3)	3327(1)	31(1)
C(21)	6539(1)	6242(2)	2822(1)	21(1)
C(22)	6918(1)	5639(3)	2111(1)	23(1)
C(23)	6758(2)	6986(3)	1563(1)	34(1)
C(24)	7059(2)	6506(4)	892(1)	47(1)
C(25)	7529(2)	4655(4)	756(1)	47(1)
C(26)	7712(2)	3303(3)	1296(1)	35(1)
C(27)	7403(1)	3766(3)	1978(1)	23(1)

C(28)	6110(1)	2124(2)	3375(1)	21(1)
C(29)	7221(1)	1668(2)	3096(1)	24(1)
C(30)	7717(1)	2289(2)	2522(1)	24(1)
C(31)	3066(2)	1094(4)	1462(1)	50(1)
C(32)	3456(2)	4533(4)	982(1)	49(1)

Bond lengths [Å] and angles [°] for CCDC: 2431435.

O(1)-C(9)	1.3465(19)	C(12)-H(12)	0.9500
O(1)-C(10)	1.4745(19)	C(13)-C(14)	1.505(2)
O(2)-C(8)	1.4239(16)	C(13)-H(13)	0.9500
O(2)-C(15)	1.4283(17)	C(14)-H(14A)	0.9900
O(3)-C(16)	1.3397(19)	C(14)-H(14B)	0.9900
O(3)-C(17)	1.4714(19)	C(15)-C(21)	1.5259(19)
O(4)-C(9)	1.214(2)	C(15)-H(15)	1.0000
O(5)-C(16)	1.216(2)	C(17)-C(31)	1.511(3)
N(1)-C(9)	1.3684(19)	C(17)-C(32)	1.520(3)
N(1)-C(8)	1.4495(18)	C(17)-C(18)	1.526(3)
N(1)-C(14)	1.4578(19)	C(18)-H(18A)	0.9800
N(2)-C(16)	1.3667(19)	C(18)-H(18B)	0.9800
N(2)-C(15)	1.454(2)	C(18)-H(18C)	0.9800
N(2)-C(28)	1.4558(18)	C(19)-H(19A)	0.9800
C(1)-C(2)	1.396(2)	C(19)-H(19B)	0.9800
C(1)-C(6)	1.405(2)	C(19)-H(19C)	0.9800
C(1)-C(7)	1.5094(19)	C(20)-H(20A)	0.9800
C(2)-C(3)	1.390(2)	C(20)-H(20B)	0.9800
C(2)-H(2)	0.9500	C(20)-H(20C)	0.9800
C(3)-C(4)	1.383(2)	C(21)-C(22)	1.501(2)
C(3)-H(3)	0.9500	C(21)-H(21A)	0.9900
C(4)-C(5)	1.388(2)	C(21)-H(21B)	0.9900
C(4)-H(4)	0.9500	C(22)-C(23)	1.396(2)
C(5)-C(6)	1.404(2)	C(22)-C(27)	1.403(2)
C(5)-H(5)	0.9500	C(23)-C(24)	1.382(3)
C(6)-C(12)	1.479(2)	C(23)-H(23)	0.9500
C(7)-C(8)	1.5278(19)	C(24)-C(25)	1.384(4)
C(7)-H(7A)	0.9900	C(24)-H(24)	0.9500
C(7)-H(7B)	0.9900	C(25)-C(26)	1.391(3)
C(8)-H(8)	1.0000	C(25)-H(25)	0.9500
C(10)-C(20)	1.517(3)	C(26)-C(27)	1.401(2)
C(10)-C(19)	1.520(2)	C(26)-H(26)	0.9500
C(10)-C(11)	1.522(3)	C(27)-C(30)	1.486(2)
C(11)-H(11A)	0.9800	C(28)-C(29)	1.503(2)
C(11)-H(11B)	0.9800	C(28)-H(28A)	0.9900
C(11)-H(11C)	0.9800	C(28)-H(28B)	0.9900
C(12)-C(13)	1.330(2)	C(29)-C(30)	1.329(2)

C(29)-H(29)	0.9500	C(8)-C(7)-H(7B)	109.3
C(30)-H(30)	0.9500	H(7A)-C(7)-H(7B)	107.9
C(31)-H(31A)	0.9800	O(2)-C(8)-N(1)	111.84(12)
C(31)-H(31B)	0.9800	O(2)-C(8)-C(7)	105.14(11)
C(31)-H(31C)	0.9800	N(1)-C(8)-C(7)	111.34(12)
C(32)-H(32A)	0.9800	O(2)-C(8)-H(8)	109.5
C(32)-H(32B)	0.9800	N(1)-C(8)-H(8)	109.5
C(32)-H(32C)	0.9800	C(7)-C(8)-H(8)	109.5
C(9)-O(1)-C(10)	120.08(13)	O(4)-C(9)-O(1)	125.48(14)
C(8)-O(2)-C(15)	114.70(11)	O(4)-C(9)-N(1)	124.55(14)
C(16)-O(3)-C(17)	120.61(14)	O(1)-C(9)-N(1)	109.97(13)
C(9)-N(1)-C(8)	119.21(12)	O(1)-C(10)-C(20)	109.83(13)
C(9)-N(1)-C(14)	122.18(13)	O(1)-C(10)-C(19)	110.84(14)
C(8)-N(1)-C(14)	117.90(12)	C(20)-C(10)-C(19)	111.96(17)
C(16)-N(2)-C(15)	124.15(13)	O(1)-C(10)-C(11)	101.79(15)
C(16)-N(2)-C(28)	117.01(12)	C(20)-C(10)-C(11)	111.36(15)
C(15)-N(2)-C(28)	117.12(12)	C(19)-C(10)-C(11)	110.62(15)
C(2)-C(1)-C(6)	119.36(13)	C(10)-C(11)-H(11A)	109.5
C(2)-C(1)-C(7)	118.76(13)	C(10)-C(11)-H(11B)	109.5
C(6)-C(1)-C(7)	121.86(14)	H(11A)-C(11)-H(11B)	109.5
C(3)-C(2)-C(1)	121.17(14)	C(10)-C(11)-H(11C)	109.5
C(3)-C(2)-H(2)	119.4	H(11A)-C(11)-H(11C)	109.5
C(1)-C(2)-H(2)	119.4	H(11B)-C(11)-H(11C)	109.5
C(4)-C(3)-C(2)	119.76(15)	C(13)-C(12)-C(6)	133.39(14)
C(4)-C(3)-H(3)	120.1	C(13)-C(12)-H(12)	113.3
C(2)-C(3)-H(3)	120.1	C(6)-C(12)-H(12)	113.3
C(3)-C(4)-C(5)	119.69(14)	C(12)-C(13)-C(14)	131.73(15)
C(3)-C(4)-H(4)	120.2	C(12)-C(13)-H(13)	114.1
C(5)-C(4)-H(4)	120.2	C(14)-C(13)-H(13)	114.1
C(4)-C(5)-C(6)	121.42(15)	N(1)-C(14)-C(13)	116.45(13)
C(4)-C(5)-H(5)	119.3	N(1)-C(14)-H(14A)	108.2
C(6)-C(5)-H(5)	119.3	C(13)-C(14)-H(14A)	108.2
C(5)-C(6)-C(1)	118.55(14)	N(1)-C(14)-H(14B)	108.2
C(5)-C(6)-C(12)	115.74(13)	C(13)-C(14)-H(14B)	108.2
C(1)-C(6)-C(12)	125.47(13)	H(14A)-C(14)-H(14B)	107.3
C(1)-C(7)-C(8)	111.82(11)	O(2)-C(15)-N(2)	110.34(12)
C(1)-C(7)-H(7A)	109.3	O(2)-C(15)-C(21)	105.84(12)
C(8)-C(7)-H(7A)	109.3	N(2)-C(15)-C(21)	111.07(12)
C(1)-C(7)-H(7B)	109.3	O(2)-C(15)-H(15)	109.8

N(2)-C(15)-H(15)	109.8
C(21)-C(15)-H(15)	109.8
O(5)-C(16)-O(3)	126.03(15)
O(5)-C(16)-N(2)	122.92(15)
O(3)-C(16)-N(2)	111.05(13)
O(3)-C(17)-C(31)	110.56(17)
O(3)-C(17)-C(32)	100.83(16)
C(31)-C(17)-C(32)	111.72(17)
O(3)-C(17)-C(18)	109.74(14)
C(31)-C(17)-C(18)	112.5(2)
C(32)-C(17)-C(18)	110.88(19)
C(17)-C(18)-H(18A)	109.5
C(17)-C(18)-H(18B)	109.5
H(18A)-C(18)-H(18B)	109.5
C(17)-C(18)-H(18C)	109.5
H(18A)-C(18)-H(18C)	109.5
H(18B)-C(18)-H(18C)	109.5
C(10)-C(19)-H(19A)	109.5
C(10)-C(19)-H(19B)	109.5
H(19A)-C(19)-H(19B)	109.5
C(10)-C(19)-H(19C)	109.5
H(19A)-C(19)-H(19C)	109.5
H(19B)-C(19)-H(19C)	109.5
C(10)-C(20)-H(20A)	109.5
C(10)-C(20)-H(20B)	109.5
H(20A)-C(20)-H(20B)	109.5
C(10)-C(20)-H(20C)	109.5
H(20A)-C(20)-H(20C)	109.5
H(20B)-C(20)-H(20C)	109.5
C(22)-C(21)-C(15)	110.19(12)
C(22)-C(21)-H(21A)	109.6
C(15)-C(21)-H(21A)	109.6
C(22)-C(21)-H(21B)	109.6
C(15)-C(21)-H(21B)	109.6
H(21A)-C(21)-H(21B)	108.1
C(23)-C(22)-C(27)	119.33(16)
C(23)-C(22)-C(21)	118.24(15)
C(27)-C(22)-C(21)	122.42(14)
C(24)-C(23)-C(22)	121.34(19)
C(24)-C(23)-H(23)	119.3
C(22)-C(23)-H(23)	119.3
C(23)-C(24)-C(25)	119.58(19)
C(23)-C(24)-H(24)	120.2
C(25)-C(24)-H(24)	120.2
C(24)-C(25)-C(26)	120.02(18)
C(24)-C(25)-H(25)	120.0
C(26)-C(25)-H(25)	120.0
C(25)-C(26)-C(27)	120.91(19)
C(25)-C(26)-H(26)	119.5
C(27)-C(26)-H(26)	119.5
C(26)-C(27)-C(22)	118.80(16)
C(26)-C(27)-C(30)	116.39(16)
C(22)-C(27)-C(30)	124.53(15)
N(2)-C(28)-C(29)	115.82(13)
N(2)-C(28)-H(28A)	108.3
C(29)-C(28)-H(28A)	108.3
N(2)-C(28)-H(28B)	108.3
C(29)-C(28)-H(28B)	108.3
H(28A)-C(28)-H(28B)	107.4
C(30)-C(29)-C(28)	130.97(14)
C(30)-C(29)-H(29)	114.5
C(28)-C(29)-H(29)	114.5
C(29)-C(30)-C(27)	132.08(14)
C(29)-C(30)-H(30)	114.0
C(27)-C(30)-H(30)	114.0
C(17)-C(31)-H(31A)	109.5
C(17)-C(31)-H(31B)	109.5
H(31A)-C(31)-H(31B)	109.5
C(17)-C(31)-H(31C)	109.5
H(31A)-C(31)-H(31C)	109.5
H(31B)-C(31)-H(31C)	109.5
C(17)-C(32)-H(32A)	109.5
C(17)-C(32)-H(32B)	109.5
H(32A)-C(32)-H(32B)	109.5
C(17)-C(32)-H(32C)	109.5
H(32A)-C(32)-H(32C)	109.5
H(32B)-C(32)-H(32C)	109.

Anisotropic displacement parameters (Å^{2}x 10^3) for CCDC: 2431435. The anisotropic displacement factor exponent takes the form: $-2\pi^2$[h^2 $a^{*2}U^{11}$ + ... + 2 h k a* b* U^{12}]

	U^{11}	U^{22}	U^{33}	U^{23}	U^{13}	U^{12}
O(1)	18(1)	26(1)	32(1)	3(1)	-8(1)	1(1)
O(2)	14(1)	26(1)	20(1)	-4(1)	2(1)	-3(1)
O(3)	23(1)	35(1)	18(1)	-6(1)	-7(1)	5(1)
O(4)	22(1)	21(1)	28(1)	-3(1)	-4(1)	-3(1)
O(5)	22(1)	27(1)	33(1)	-8(1)	-1(1)	-1(1)
N(1)	15(1)	17(1)	20(1)	1(1)	-1(1)	-1(1)
N(2)	15(1)	21(1)	18(1)	0(1)	-2(1)	0(1)
C(1)	17(1)	22(1)	15(1)	-4(1)	0(1)	-1(1)
C(2)	22(1)	22(1)	18(1)	-3(1)	-2(1)	2(1)
C(3)	28(1)	24(1)	16(1)	1(1)	-1(1)	-6(1)
C(4)	21(1)	32(1)	18(1)	-2(1)	1(1)	-6(1)
C(5)	18(1)	27(1)	20(1)	-3(1)	0(1)	1(1)
C(6)	18(1)	19(1)	18(1)	-4(1)	0(1)	-1(1)
C(7)	15(1)	25(1)	18(1)	-2(1)	0(1)	-1(1)
C(8)	14(1)	20(1)	17(1)	-1(1)	1(1)	0(1)
C(9)	15(1)	22(1)	18(1)	1(1)	0(1)	-1(1)
C(10)	15(1)	42(1)	27(1)	-9(1)	-5(1)	2(1)
C(11)	28(1)	54(1)	39(1)	0(1)	-9(1)	15(1)
C(12)	23(1)	16(1)	31(1)	-5(1)	3(1)	1(1)
C(13)	27(1)	14(1)	35(1)	0(1)	1(1)	0(1)
C(14)	22(1)	17(1)	29(1)	3(1)	2(1)	-4(1)
C(15)	16(1)	21(1)	17(1)	0(1)	1(1)	-1(1)
C(16)	14(1)	27(1)	20(1)	-3(1)	0(1)	0(1)
C(17)	26(1)	59(1)	24(1)	-19(1)	-11(1)	15(1)
C(18)	44(1)	90(2)	26(1)	-18(1)	-6(1)	35(1)
C(19)	20(1)	65(1)	30(1)	-19(1)	-5(1)	5(1)
C(20)	20(1)	40(1)	34(1)	-10(1)	2(1)	-2(1)
C(21)	16(1)	21(1)	25(1)	2(1)	3(1)	-2(1)
C(22)	16(1)	29(1)	24(1)	5(1)	3(1)	2(1)
C(23)	29(1)	40(1)	34(1)	15(1)	7(1)	8(1)
C(24)	40(1)	73(2)	29(1)	23(1)	9(1)	15(1)
C(25)	39(1)	80(2)	23(1)	5(1)	10(1)	14(1)
C(26)	24(1)	53(1)	28(1)	-4(1)	6(1)	9(1)
C(27)	14(1)	31(1)	24(1)	2(1)	2(1)	3(1)

C(28)	20(1)	22(1)	20(1)	4(1)	-3(1)	-2(1)
C(29)	18(1)	22(1)	32(1)	5(1)	-7(1)	0(1)
C(30)	14(1)	23(1)	33(1)	1(1)	-2(1)	3(1)
C(31)	27(1)	63(1)	61(1)	-34(1)	-18(1)	6(1)
C(32)	45(1)	76(2)	26(1)	-14(1)	-15(1)	30(1)

Hydrogen coordinates (x 10^4) and isotropic displacement parameters ($Å^2$x 10^{3}) for CCDC: 2431435.

	x	y	z	U(eq)
H(2)	3456	2787	5319	25
H(3)	1830	1813	5838	27
H(4)	331	3960	5799	28
H(5)	465	7067	5242	26
H(7A)	4599	5360	4881	23
H(7B)	4124	7527	4678	23
H(8)	3966	4007	3807	20
H(11A)	-766	7524	2406	60
H(11B)	340	8556	2158	60
H(11C)	-28	8720	2953	60
H(12)	1583	9747	4854	28
H(13)	2377	11035	4000	30
H(14A)	2961	9249	3042	27
H(14B)	3959	9220	3569	27
H(15)	4882	5997	2615	21
H(18A)	4505	1529	349	80
H(18B)	5069	474	1004	80
H(18C)	5413	2708	782	80
H(19A)	-91	4270	1907	58
H(19B)	1012	3304	2190	58
H(19C)	1048	5250	1699	58
H(20A)	-664	4567	3237	47
H(20B)	175	5691	3740	47
H(20C)	488	3566	3403	47
H(21A)	6528	7730	2859	25
H(21B)	7047	5715	3177	25
H(23)	6436	8259	1654	41
H(24)	6944	7442	525	57
H(25)	7727	4307	295	57
H(26)	8052	2049	1201	42
H(28A)	5757	834	3499	25
H(28B)	6193	2913	3808	25
H(29)	7639	786	3378	29
H(30)	8402	1673	2446	28

H(31A)	2636	808	1045	75
H(31B)	2595	1638	1825	75
H(31C)	3406	-152	1627	75
H(32A)	3079	4213	548	73
H(32B)	4039	5499	889	73
H(32C)	2943	5122	1313	73

4 References

(1) Kessler, S. N.; Neuburger, M.; Wegner, H. A. Bidentate Lewis Acids for the Activation of 1,2-Diazines – A New Mode of Catalysis. *Eur. J. Org. Chem.* **2011**, *2011* (17), 3238–3245. DOI: 10.1002/ejoc.201100335.

(2) Kessler, S. N.; Wegner, H. A. One-pot synthesis of phthalazines and pyridazino-aromatics: a novel strategy for substituted naphthalenes. *Org. Lett.* **2012**, *14* (13), 3268–3271. DOI: 10.1021/ol301167q.

(3) Kessler, S. N.; Neuburger, M.; Wegner, H. A. Domino inverse electron-demand Diels-Alder/cyclopropanation reaction of diazines catalyzed by a bidentate Lewis acid. *J. Am. Chem. Soc.* **2012**, *134* (43), 17885–17888. DOI: 10.1021/ja308858y.

(4) Große, M.; Wegner, H. A. Bidentate Lewis Acid-Catalyzed Inverse Electron-Demand Diels–Alder Reaction of Phthalazines and Cyclooctynes. *Synlett* **2024**, *35* (09), 1019–1022. DOI: 10.1055/a-2204-9522.

(5) Synthesis of 6,7-Dichloro-5,8-phthalazinedione and Its Derivatives. *Bull. Korean Chem. Soc.* **2002**, *23* (10), 1425–1446. DOI: 10.5012/bkcs.2002.23.10.1425.

(6) Berthold, H.; Schotten, T.; Hoffmann, F.; Thiem, J. A Highly Versatile Octasubstituted Phthalocyanine Scaffold for ex post Chemical Diversification. *Synthesis* **2010**, *2010* (05), 741–748. DOI: 10.1055/s-0029-1218602.

(7) Lutz, J. P.; Davydovich, O.; Hannigan, M. D.; Moore, J. S.; Zimmerman, P. M.; McNeil, A. J. Functionalized and Degradable Polyphthalaldehyde Derivatives. *J. Am. Chem. Soc.* **2019**, *141* (37), 14544–14548. DOI: 10.1021/jacs.9b07508.

(8) Hodgson, D. M.; Pearson, C. I.; Kazmi, M. Generation and electrophile trapping of N-Boc-2-lithio-2-azetine: synthesis of 2-substituted 2-azetines. *Org. Lett.* **2014**, *16* (3), 856–859. DOI: 10.1021/ol403626k.

(9) Su, C.; Dallaston, M. A.; Watson, R. D.; Fahrenhorst-Jones, T.; Cameron, J. P.; Pierens, G. K.; Bernhardt, P. V.; Savage, G. P.; Williams, C. M. The (±)-5-Aza1.0triblattane Skeleton via Azetine Cycloaddition. *Org. Lett.* **2024**, *26* (14), 2827–2831. DOI: 10.1021/acs.orglett.3c03655.

(10) Krause, L.; Herbst-Irmer, R.; Sheldrick, G. M.; Stalke, D. Comparison of silver and molybdenum microfocus X-ray sources for single-crystal structure determination. *J. Appl. Crystallogr.* **2015**, *48* (Pt 1), 3–10. DOI: 10.1107/S1600576714022985.

(11) Sheldrick, G. M. SHELXT - integrated space-group and crystal-structure determination. *Acta Crystallogr. A* **2015**, *71* (Pt 1), 3–8. DOI: 10.1107/S2053273314026370.

(12) Sheldrick, G. M. Crystal structure refinement with SHELXL. *Acta Crystallogr. C* **2015**, *71* (Pt 1), 3–8. DOI: 10.1107/S2053229614024218.

(13) Spek, A. L. PLATON SQUEEZE: a tool for the calculation of the disordered solvent contribution to the calculated structure factors. *Acta Crystallogr. C* **2015**, *71* (Pt 1), 9–18. DOI: 10.1107/S2053229614024929.
(14) Spek, A. L. Single-crystal structure validation with the program PLATON. *J. Appl. Crystallogr.* **2003**, *36* (1), 7–13. DOI: 10.1107/S0021889802022112.
(15) Spek, A. L. Structure validation in chemical crystallography. *Acta Crystallogr. D* **2009**, *65* (Pt 2), 148–155. DOI: 10.1107/S090744490804362X.
(16) Spek, A. L. What makes a crystal structure report valid? *Inorg. Chim. Acta* **2018**, *470*, 232–237. DOI: 10.1016/j.ica.2017.04.036.
(17) Spek, A. L. checkCIF validation ALERTS: what they mean and how to respond. *Acta Crystallogr. E* **2020**, *76* (Pt 1), 1–11. DOI: 10.1107/S2056989019016244.
